# Integrated metagenomic and metabonomic mechanisms for the therapeutic effects of Duhuo Jisheng decoction on intervertebral disc degeneration

**DOI:** 10.1371/journal.pone.0310014

**Published:** 2024-10-17

**Authors:** Chao Song, Fei Liu, Yongliang Mei, Weiye Cai, Kang Cheng, Daru Guo, Yong Liu, Houyin Shi, Dayue Darrel Duan, Zongchao Liu

**Affiliations:** 1 Department of Orthopedics and Traumatology (Trauma and Bone-Setting), The Affiliated Traditional Chinese Medicine Hospital, Southwest Medical University, Luzhou, Sichuan Province, China; 2 Center for Phenomics of Traditional Chinese Medicine, The Affiliated Traditional Chinese Medicine Hospital, Southwest Medical University, Luzhou, Sichuan Province, China; 3 Luzhou Longmatan District People’s Hospital, Luzhou, Sichuan Province, China; University of Jeddah, SAUDI ARABIA

## Abstract

Intervertebral disc degeneration (IVDD) is a prevalent orthopedic condition with lower back pain as the predominant clinical presentation that challenges clinical treatment with few therapeutic options. Duhuo Jisheng Decoction (DHJSD) has been proven effective in the therapy of IVDD, but the precise underlying mechanisms remain not fully elucidated. The current study was designed to test our hypothesis that DHJSD may systematically correct the phenotypic disruption of the gut microbiota and changes in the serum metabolome linked to IVDD. Analysis of the active ingredients of DHJSD by ultra high performance liquid chromatography. An integrated metagenomic and metabonomic approach was used to analyze feces and blood samples from normal and IVDD rats. Compared to the control group, fiber ring pinning on the caudal 3 to caudal 5 segments of the rats caused IVDD and significantly altered the compositions of the intestinal microbiota and serum metabolites. Integrated analysis revealed commonly-altered metabolic pathways shared by both intestinal microbiota and serum metabolome of the IVDD rats. DHJSD inhibited the degenerative process and restored the compositions of the perturbed gut microbiota, particularly the relative abundance of commensal microbes of the *Prevotellaceae* family. DHJSD also corrected the altered metabolic pathways involved in the metabolism of glycine, serine, threonine, valine, the citric acid cycle, and biosynthesis of leucine and isoleucine. DHJSD inhibited the disc degeneration process by an integrated metagenomic and metabonomic mechanism to restore the microbiome profile and normalize the metabonomic pathways.

## Introduction

Intervertebral disc degeneration (IVDD) is a chronic degenerative illness characterized by low back pain (LBP) due to herniated nucleus pulposus-caused nerve root compression and/or intervertebral disc structural degeneration-caused inflammation [1]. LBP affects around 40% of the population worldwide and has a significant negative impact on life quality of patients [2,3]. There are currently few therapeutic options available for IVDD because the pathophysiology of the disease is largely unknown. Surgery is often reserved for individuals with significant lumbar disc herniation, LBP and leg pain, while conservative therapies, including anti-inflammatory medications, hormones, and physical manipulation, remain the mainstay of clinical management of IVDD [4]. Although conventional therapeutic modalities can provide temporary relief of LBP, they are hampered by high recurrence, large financial burden, and serious side effects [5]. Thus, there is an urgent need to discover new therapeutic targets and medications based on new mechanistic insights to address IVDD.

Traditional Chinese medicine (TCM) has been clinically proven effective in treating and preventing degenerative diseases with precise, safe, and dependable efficacy [6]. Thus, TCM has been serving as a crucial complementary and alternative medicine in the treatment of orthopedic disorders [6]. It has also been demonstrated to alleviate pain and postpone disc degeneration [7]. Among the TCM treatments for orthopedic illnesses, Duhuo Jisheng Decoction (DHJSD) has demonstrated good efficacy in the management of osteoporosis, osteoarthritis of the knee, rheumatoid arthritis, lumbar disc herniation, etc [8,9]. DHJSD is from Sun Simiao’s *<Tousand Golden-Prescriptions>* in the Tang Dynasty, a formula that was, at the earliest, used to treat lumbar back pain. Low back pain is very similar to the cause and mechanism of what we consider "paralysis ", so this formula is also commonly used to treat paralysis in clinical practice [10,11]. Previous research from us and others has revealed that DHJSD has effects of anti-inflammation, analgesic, vasodilation, inhibition of platelet aggregation, and modulation of immune function [9,12,13]. Currently, we found that its mechanism of action mainly involves the regulation of inflammatory factors, reduction of apoptosis and pyroptosis of nucleus pulpous cells (NPCs), inhibition of extracellular matrix degradation, and improvement of bacteria in the gut [14]. DHJSD has also been widely used in the treatment of IVDD [9,15], but the precise mechanisms for its actions remain obscure owing to its complex compositions with numerous potential bioactive components and drug targets.

A growing body of evidence shows a strong correlation between gut microbiota and bone metabolism [16–18]. Gut microorganisms may alter bone disease by controlling dynamic bone metabolism and homeostasis [17]. The processes largely involve the control of food absorption and intestinal permeability of metabolites such as short-chain amino acids (SCFAs) by gut microorganisms [18], and immunological response by hormones or neurotransmitters such as 5-hydroxytryptamine [19]. However, it is still unknown whether TCM therapy of orthopedic illnesses is through an integrated metagenomic and metabolomic mechanism. The current study was designed to test our hypothesis that DHJSD may systematically correct the phenotypic disruption of the gut microbiota and changes in the serum metabolome linked to the pathogenesis of IVDD. An integrated metagenomic and metabonomic approach was used to analyze feces and blood samples from normal and IVDD rats. We found that in the IVDD rats both the gut microbiota and the serum metabonomic pathways related to inflammation and immune responses were altered. DHJSD inhibited the disc degeneration process by an integrated restoration of the microbiome profile and the metabonomic pathways.

## Materials and methods

### 1 Preparation of DHJSD pellet samples and analysis of active ingredients

Beijing Tong Ren Tang Co. supplied the DHJSD herbs. Prescriptions: Aralia fargesii Franch., (Du Huo), No. 201201, Origin: Sichuan, Weight 9g. Taxillus chinensis (DC.) Danser, (Sang Jisheng), Batch No. 201100601, Origin: Guangxi, Weight 6g. Eucommia ulmoides Oliv., (Du Zhong), Batch No. 201105–1, Origin: Sichuan, Weight 6g. Achyranthes bidentata Blume (Niu Xi), Batch No. 201101, Origin: Henan, Weight 6g. Ginkgo biloba L., (Xi Xin), Batch No. 201001, Origin: Liaoning, Weight 6g. Zanthoxylum schinifolium Siebold & Zucc., (Qin Jiao), Batch No.: 200301, Origin: Sichuan, Weight 6g. Smilax glabra Roxb., (Fu Ling), Batch No.: 20122901, Origin: Anhui, Weight 6g. Cinnamomum verum J.Presl, (Rou Gui), Batch No.: 201101071, Origin: Guangxi, Weight 6g. Carum carvi L., (Fang Feng), Batch No.: 201101, Origin: Inner Mongolia, Weight 6g. Conioselinum anthriscoides (H.Boissieu) Pimenov & Kljuykov, (Chuan Xiong), Batch No.: 201202, Origin: Sichuan, Weight 6g. Angelica sinensis (Oliv.) Diels, (Dang Shen) Batch No.: 201201, Origin: Gansu. Glycyrrhiza glabra L., (Gan Cao), Batch No.: 20110101, Origin: Gansu, Weight 6g. Levisticum officinale W.D.J.Koch, (Dang Gui), Batch No.: 20110101, Origin: Gansu. Aloe vera (L.) Burm.f., (Sao Yao) Batch No.: 20210401, Origin: Anhui. Digitalis purpurea L., (Di Huang), Batch No.: 20120101, Origin: Henan, Weight 6g.

Ultracentrifugation and ultrafiltration were used to eliminate contaminants from the herbs before preparing them into DHJSD concentrated granules. The plant name was checked with http://mpns.kew.org, access date of February 21, 2023. Five grams of DHJSD concentrated pellet powder, 8 times as much 100% ethanol, 250 W of ultrasonic power at 40 kHz, cooling to ambient temperature, 10 min of centrifuging at 12000 rpm, and collection of the supernatant A. Then, after adding 8 times as much water to the precipitate and extracting it using ultrasonic energy at a frequency of 40 kHz for 1 hour the supernatant B was collected. Centrifuge for 10 minutes at 12000 rpm after allowing the sample to settle to ambient temperature, then collect the supernatant B. To qualitatively analyze the chemical makeup of DHJSD pellet extracts and DHJSD-containing serum, UHPLC spectra were obtained in both positive and negative ion modes. They were used to obtain quasi-molecular ion peaks to determine the precise molecular weight of the substance under test, calculate the potential elemental composition of the compound represented by this mass-to-core ratio, and then compare the theoretical isotopic distribution of this molecular formula with the actual measured values. Excimer ions with UHPLC responses greater than 10,000 were analysed with an allowed mass deviation of ±5 ppm. Positive ion searches included excimer ion peaks for [M+H]+, [M+NH4]+, [M+Na]+ and [M+K]+, and negative ion searches included excimer ion peaks for [M-H]-, [M+Cl]- and [M+HCOO]-. Accurate characterisation was obtained for all components with unique quantiles in the database; while for isomers, the compounds were characterised in combination with differences in retention behaviour on reversed-phase liquid chromatography and information on fragment ions generated in UHPLC spectra.

### 2 Animals

Thirty SD rats, male, 6–8 weeks old, weighing 250± 50 g. The rats were reared under standard conditions (temperature 25 ± 2°C, relative humidity 40 ± 10%, light–dark cycle of 12/12 h, clean bedding, and water and food ad libitum). After one week of adaptation, the rats were randomly divide into 6 groups (n = 5 for each group): The control group received no intervention (Control); The IVDD model was established by percutaneous annular fiber puncture on the trailing 3rd to 5th vertebral segments as described previously [20]. After the IVDD was successfully induced, the animals were further randomly divided into non-treatment (Model) and DHJSD or celecoxib treatment groups. The DHJSD treatment groups received DHJSD through intragastric administration at different doses of 0.16g/100g (Low), 0.32g/100g (Medium), and 0.64g/100g (High) per day, respectively, for one month; the celecoxib group (Celecoxib) received celecoxib 40 mg per day by intragastric administration for one month [9]. The animal study was reviewed and approved by Southwest Medical University’s Animal Experiment Center, Certificate of Conformity: 44005800012441, and the experimental procedure was authorized by the Ethics Committee of Southwest Medical University’s Affiliated Hospital of Traditional Chinese Medicine, TCMF-2021003.

### 3 Raw data and specimen collection

Twelve hours after the last intragastric administration, the rats were fasted overnight. And 30 mg/kg pentobarbital was given intraperitoneally before specimen collection. The rat’s tail was secured to the cardboard once the anesthetic took effect to make sure it was on a straight line. The DR instrument was used to gather images under the settings of 80 kV, 120 ms for the anterior position, and 100 kV, 160 ms for the lateral parameters. Blood samples were taken from the abdominal aorta shortly after the photographs were taken. The upper serum samples were collected in cryopreservation tubes and then kept in an ultra-low temperature refrigerator at -80°C after the blood samples were centrifuged at a low temperature and a high speed. Rat serum specimens for LC-MS Non-Targeted Metabolomics Detection [21]. After collecting blood, 1–2 pieces of intestinal excrement were placed in sterile cryopreservation tubes. Finally, the samples were placed in an ultra-low temperature refrigerator set to -80°C. Stool samples for metagenomic analysis [22]. The rats’ undamaged discs from S3 to S5 were then fixed in a 10% formalin solution, and disc histopathology sections were later taken.

### 4 Evaluation of therapeutic efficacy on IVDD

The disc height index (DHI), hematoxylin-eosin staining(HE), and Safranin O-Fast Green staining(SOFG) were used to determine the degree of disc degeneration. The DHI was determined as follows: [23]

$$\text{DHI}=2\times \left(\text{D}+\text{E}+\text{F}\right)/\left(\text{A}+\text{B}+\text{C}+\text{G}+\text{H}+\text{I}\right)$$

*Note: A, B, C,G,H and I represent the length of the vertebrae measured in different positions, D, E,F represent the length of the vertebral space measured in different positions*.

HE staining, the rat caudal vertebrae were fixed using 4% paraformaldehyde for 24 h after sampling, and then rinsed overnight with running water. The fixed tissues were transferred to EDTA decalcification solutio for 30 days. After the completion of decalcification, the tissues were embedded and tissue sections were prepared. The sections were sequentially placed in dewaxing transparent solution I for 20 min, dewaxing transparent solution II for 20 min, anhydrous ethanol for 5 min, 75% alcohol for 5 min, and washed with running water. Then, the sections were stained with hematoxylin for 1–5 minutes, rinsed under running water until the cartilage was colorless, and then soaked for 10 seconds in 1% hydrochloric acid alcohol. Then, four cylinders of anhydrous ethanol were quickly dehydrated for 5 seconds, 2 seconds, and 10 seconds, respectively, before the sections were immersed in an eosin staining solution and stained for 1 to 5 seconds. Clean xylene was transparent for 5 min, neutral resin was used to seal the slices, microscopic examination, image acquisition and analysis, and scoring using HE pathological staining.

SOFG: Make paraffin sections according to the above steps (same steps as HE staining). Then, slices should be immersed in xylene I for 20 minutes, xylene II for 20 minutes, anhydrous ethanol I for 5 minutes, anhydrous ethanol II for 5 minutes, and 75% alcohol for 5 minutes before being washed with tap water. Slices should also be immersed in solid green staining solution for 5 to 10 minutes, washed with water to remove excess staining solution until the cartilage is colorless, slightly soaked in differentiation solution, and then washed. The collagen content specific gravity score was used to undertake microscopic examination, image collection, and analysis.

### 5 Metagenomic analysis of fecal bacteria

Sample genomic DNA was extracted from the colon contents using the CTAB method, and DNA concentration was quantified using Qubit. The libraries were constructed using the NEBNext^®^ Ultra DNA Library Prep Kit for Illumina (NEB, USA) according to the standard protocol provided by Shanghai Biomedical Technology Co. After passing the library test, the different libraries were pooled according to the effective concentration and the target downstream data volume required for Illumina PE150 sequencing. The original data were spliced and filtered, followed by operational taxonomic unit (OTU) clustering and species classification analysis to obtain information on species richness and uniformity within the samples, as well as common and unique operational taxonomic unit information among different samples or groups. Based on the gene catalogue (Unigenes) that will eventually be used for subsequent analysis [24], Principal coordinate analysis (PCoA), principal component analysis (PCA) and other dimensionality reduction methods were used to analyze the differences in community structure among the samples or groups. The Student’s t test, LEfSe and other statistical analysis methods were conducted to test the significance of species composition and community structure.

### 6 Western blot

Each group’s disc tissues were homogenized and lysed with 1 ml of ice lysis buffer after being rinsed twice with cold phosphate-buffered saline (PBS). The supernatant was collected by centrifugation at 12,000 rpm for 20 min at 4°C following a 20-minute incubation on ice. A bis-chondroitin acid (BCA) protein assay kit was used to measure the amount of total protein present. SDS-PAGE was used to extract identical quantities of proteins from each sample; the separated proteins were then transferred to polyvinylidene difluoride (PVDF) membranes. The membranes were incubated with the primary antibody for a whole night at 4°C after being sealed with 5% skim milk for 2 hours at room temperature. The PVDF membrane was then treated with the secondary antibody for 2 hours at room temperature. As the internal standard, -actin was chosen as the reference protein. ImageJ software version 1.8 was used to perform densitometric analysis on the pictures.

### 7 qPCR analysis

A total RNA extraction kit was used to extract total RNA from tissue samples. Then, using the iScriptcDNA synthesis kit, 1 g of total RNA was reverse-transcribed into cDNA. Relative mRNA levels were computed, GAPDH normalization was carried out using the 2−ΔΔCt technique, and real-time quantitative PCR (qPCR) analysis was carried out using the Bio-RadCFX96 equipment [25]. The genes encoding CASP8, TNF-α, and IL-3’s final list of primer sequences was acquired (S12 Table in S4 File).

### 8 Detection of serum metabolites

One hundred microliters of sample were pipetted into an EP tube, 400 μL was extraction solution was added, vortexed and mixed for 30 seconds. This was sonicated for 10 minutes, allowed to stand at -40 oC for 15 minutes at 12000 rpm. The supernatant was put into the injection bottle and detected on the machine, and all samples were mixed into QC samples with equal amounts of supernatant. The target compounds were separated using a Vanquish (Thermo Fisher Scientific) ultra performance liquid chromatograph on a Waters ACQUITY UPLC BEH Amide (2.1 mm100 mm, 1.7 m) column. Aqueous was the A phase of liquid chromatography, and acetonitrile was the B phase. Temperature of the sample pan was 4°C, and the injection volume was 2 L. Under the control of software, the Orbitrap Exploris 120 mass spectrometer may acquire primary and secondary mass spectral data (Xcalibur, version: 4.4, Thermo). After ProteoWizard software converted the original data to mzXML format, the self-written R package (the kernel is XCMS) was used to process peak identification, peak extraction, peak alignment, and integration, and then combined with BiotreeDB (V2.1) to build a self-built secondary mass spectrometer. For substance annotation, database matching was used, and the Cutoff value for algorithm scoring was set to 0.3. The result analysis is carried out using the legitimate data that was received after data processing. Two categories—basic data analysis and advanced data analysis—dominately made up the outcome analysis. Basic data analysis used univariate statistical analysis and multivariate statistical analysis of the qualitative and quantitative results of metabolomes to identify metabolites that appear significantly different; advanced data analysis uses bioinformatics analysis based on basic data analysis to visualize and mine the biofunctions of different metabolites.

### 9 Statistical analysis

Graphpad Prism 8.0.2 software was used for statistical analysis and graphing. If the samples fell under the normal distribution, the t-test was used to compare the two groups, and the nonparametric test was used if they did not. One-way ANOVA was used to compare samples from different groups. Tukey’s method test was used to compare any two groups, with P<0.05 denoting a statistically significant difference and P<0.01 denoting a statistically significant difference.

## Results

### 1 DHJSD pellets active ingredients

**Fig 1:**
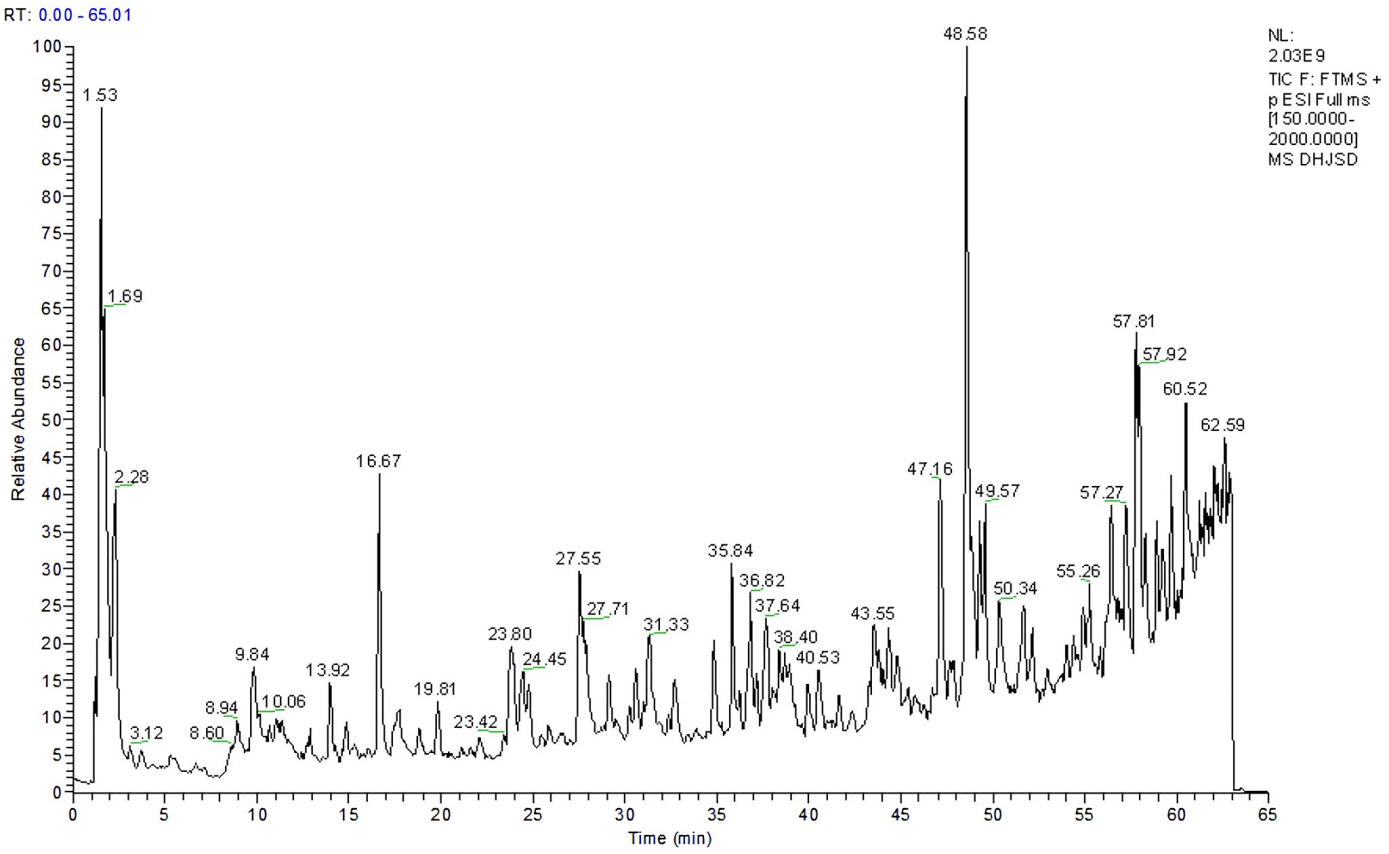
Mass spectral peak profile of DHJSD. The chromatographic peaks in the DHJSD sample solution indicated that the main compounds were bitter amygdalin, glycyrrhiza chalcone B, 2-acetoxy-3-geranyl-1,4-dihydroxybenzene, (Z)-15-oxo-11-eicosanoic acid, tetrahydrofolic acid, etc.

Two hundred and ninety-two (292) compounds were identified based on spectral data of retention time, MS, and MS/MS positive and negative modes. These compounds include 35 flavonoids, 19 alkaloids, 18 fatty acids, 18 triterpenoids, 17 fatty acyl groups, 15 sugars, 15 glycerophospholipids, 14 isoflavones, 12 steroids, 12 phenylpropanoids, and 117 other types of compounds. The chromatographic peaks in the DHJSD sample solution indicated that the main compounds were bitter amygdalin, glycyrrhiza chalcone B, 2-acetoxy-3-geranyl-1,4-dihydroxybenzene, (Z)-15-oxo-11-eicosanoic acid, tetrahydrofolic acid, etc. (Fig 1, S14 Table in S4 File).

### 2 DR and pathological evaluation of rat degenerative intervertebral discs

**Fig 2:**
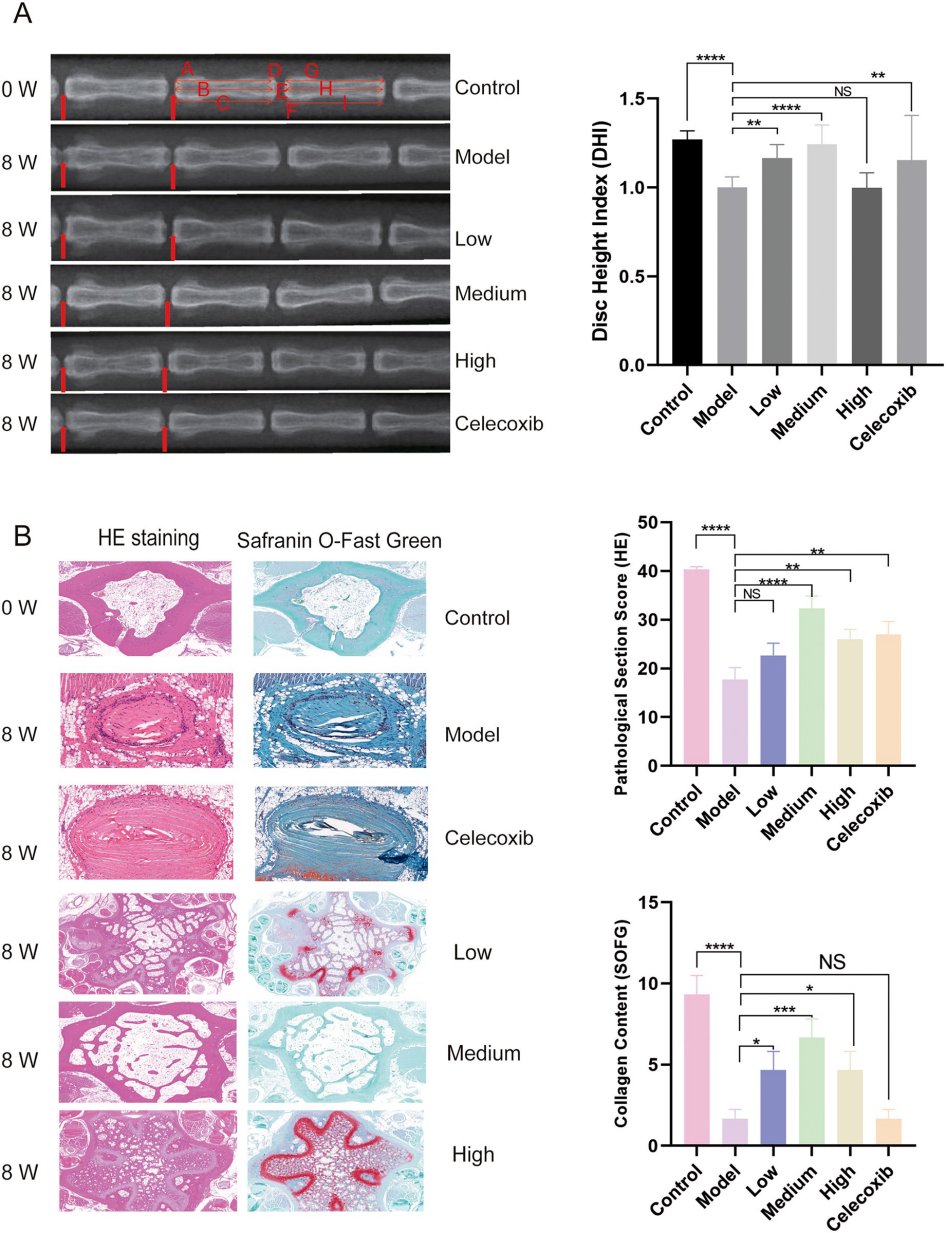
Imaging and pathological examination of deteriorated discs in rats before and after treatment with DHJSD. A. The vertebral body height of normal rats was uniform, and there was no significant difference in vertebral space; compared to the model group, the DHI indices of rats in the treatment group each recovered; compared to the high-dose group and the western medicine group, the low-dose and medium-dose groups had a significant advantage (Low< P 0.001, Medium< P 0.0001). B. Tissue staining revealed that the intervertebral disc tissue in the pathological sections of the model group was severely damaged compared to the normal group, and the nucleus pulposus showed severe degeneration and lost its normal shape; compared to the model group, the treated group had different degrees of restoration of the above structures, rich collagen content, and more neat arrangement of the fibrous rings than the control group. (HE: Medium p<0.0001,SOFG:Dose p<0.0002).

In normal rats, DR data revealed uniform vertebral body height and no discernible variation in vertebral space (Fig 2A). The rats in the Model group had notably smaller vertebral space and DHI score (dropped by 27.0%) than those in the Control group. DHI scores of rats in the DHJSD Low and Medium groups and the Celecoxib group recovered to 16.6%, 24.3%, and 15.3% in comparison to the Model group, respectively; while DHI scores of rats in the DHJSD High group experienced a 0.2% drop. It is evident that all groups had positive therapeutic effects, with the exception of the DHJSD High group, which had the poorest results. The DHJSD Medium group had the best results (S1 Table in S4 File). HE analysis of the structural alterations of the intervertebral disc, and saffron solid green staining of the collagen content of the nucleus pulposus cells (S2 Table in S4 File) [26] revealed that the longitudinal and transverse histological sections of the disc in the Model group were substantially injured and structurally disordered in comparison to the Control group. Compared with the Control group, the Model group had a 1.28-fold lower HE score, disordered fibrous rings, mucus-like degeneration, and partially fissured or split. The nucleus pulposus displayed substantial loss, losing its typical shape and being partially replaced by fibrous tissue (SOFG score was 4.6-fold lower in the Model group compared with the Control group) (Fig 2B). The nucleus pulposus appeared mildly wrinkled, the collagen content was restored, and the fibrous rings were more neatly arranged in concentric circles with smaller fissures than the Model group. However, the DHJSD group and the Celecoxib group all had varying degrees of recovery of the aforementioned structures compared to the model group. The DHJSD Medium group showed the best recovery trend, with a rise in the HE score of 83.0%. The results of the histological staining revealed that the treatment group scored much higher than the Model group, with the DHJSD Medium group showing the best recovery. The aforementioned findings show that DHJSD has the greatest effect at medium doses and can successfully postpone disc degeneration as well as reverse histopathological structural alterations that occur during the disc degeneration process.

### 3 Fecal bacteria metagenomic analysis

### 3.1 Abundance analysis of colonic microflora based on gene catalogue

**Fig 3:**
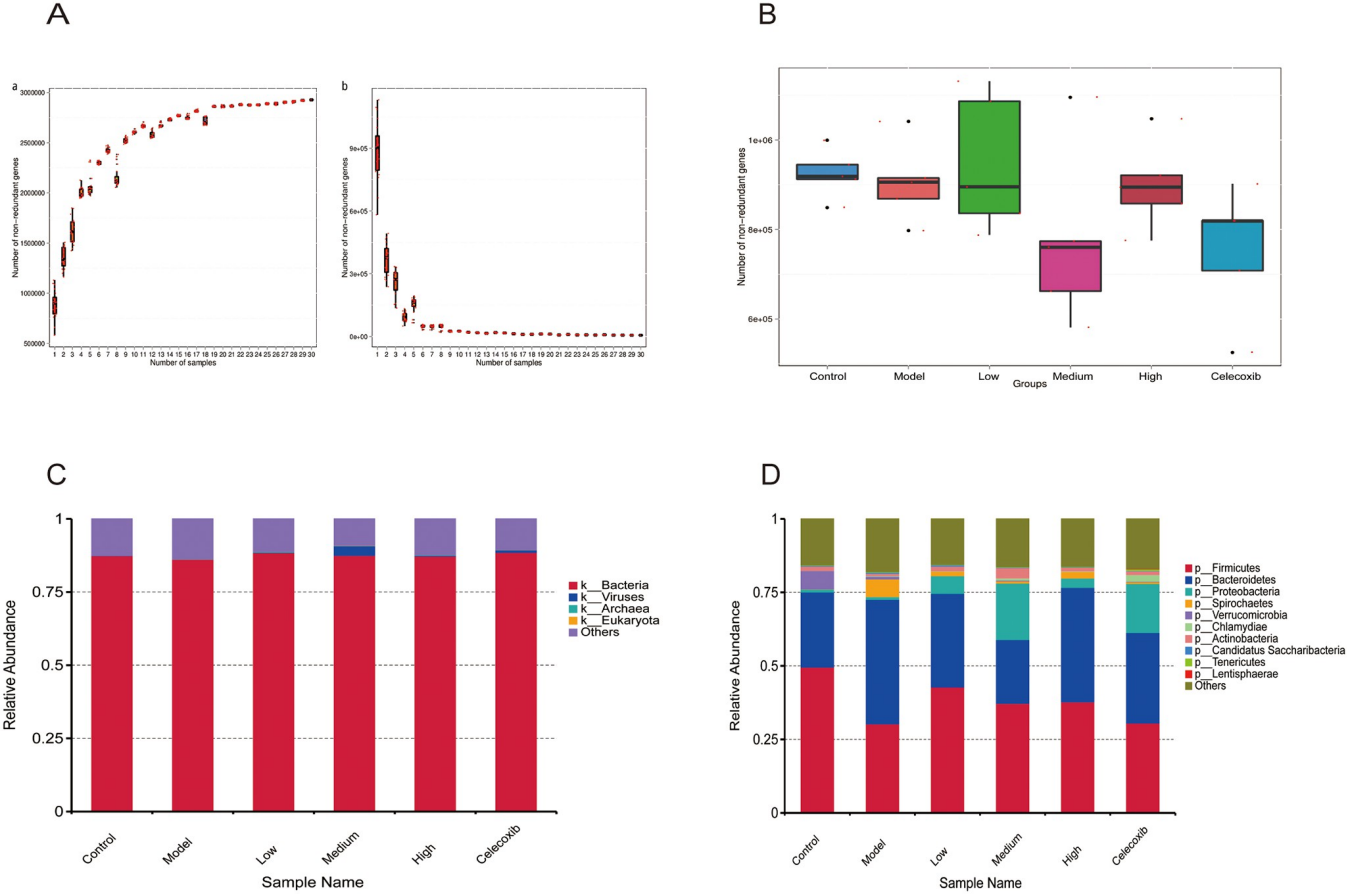
Preliminary analysis of intestinal flora in degenerated disc rats and after DHJSD treatment. A. The core-pan gene dilution curve is gradually smoothed, the sample sequenced in this experiment is sufficient, and this study is sufficient to reflect the sample population’s features. B. Box plots of gene number differences across groups when p<0.1 is regarded statistically significant, that is, gene abundance is considered statistically distinct, Medium p = 0.062; C. Annotated rat intestinal flora results at the boundary level, the largest weight of bacteria, approximately 0.85; D. Firmicutes, bacteroidetes, and proteobacteria exhibited major biological effects, according to bar graphs indicating relative abundance of species at the phylum level.

As shown in Fig 3A, the Core and Pan gene dilution curves produced from the gene catalogue information (Table 1) eventually flattened out, indicating that the samples sequenced in this experiment were sufficient to reflect the colony characteristics. The numbers of the genes in the gut microbiota decreased during disc degeneration (Fig 3B). DHJSD treatment lowered the numbers of genes in the gut microbiota even further, with a considerable and statistically significant decrease in the DHJSD Medium group (S3 Table in S1 File). The results of the kingdom-level annotation of the rat intestinal flora revealed that bacteria, which account for approximately 87% of the total species, might have a significant biological effect (Fig 3C), and that the regulation of the microbiota primarily involves bacteria, eukaryotes, archaea, and viruses (Table 2). The results showed that *Firmicutes*, *Bacteroidetes*, and *Proteobacteria* were the main dominant groups of intestinal microbes, playing a significant biological role based on the differential abundance statistics of phylum-level flora (S4 Table in S4 File, Fig 3D). The information demonstrated that *Firmicutes* and *Bacteroidetes* courses in the intestinal microorganisms of rats changed in a downward and upward direction, respectively, during the degeneration of the intervertebral disc, while *Proteobacteria* did not significantly change. The proportion of Firmicutes and *Proteobacteria* could be increased and the fraction of *Bacteroidetes* decreased, respectively, in response to DHJSD treatment.

**Table 1:** Gene catalogue basic information statistics table.

ORFs NO.	3,239,203
integrity:end	646,909(19.97%)
integrity:all	1,528,602(47.19%)
integrity:none	314,950(9.72%)
integrity:start	748,742(23.12%)
Total Len.(Mbp)	2,203
Average Len.(bp)	680
GC percent	49

**Table 2:** Kingdom-level species abundance statistics.

kingdom	k__Bacteria	k__Viruses	k__Archaea	k__Eukaryota	Others
normal	0.874328	0.000537	0.000603	0.00012	0.124414
model	0.861824	0.000412	0.000585	0.000382	0.136798
low	0.884281	0.001056	0.002104	0.000178	0.112383
Dose	0.874886	0.032484	0.001083	0.000523	0.091025
High	0.872783	0.002141	0.001653	0.000176	0.123249
Western	0.884963	0.008845	0.000325	0.000469	0.1054

#### 3.2 Sample clustering and non-metric multi-dimensional scaling analysis

**Fig 4:**
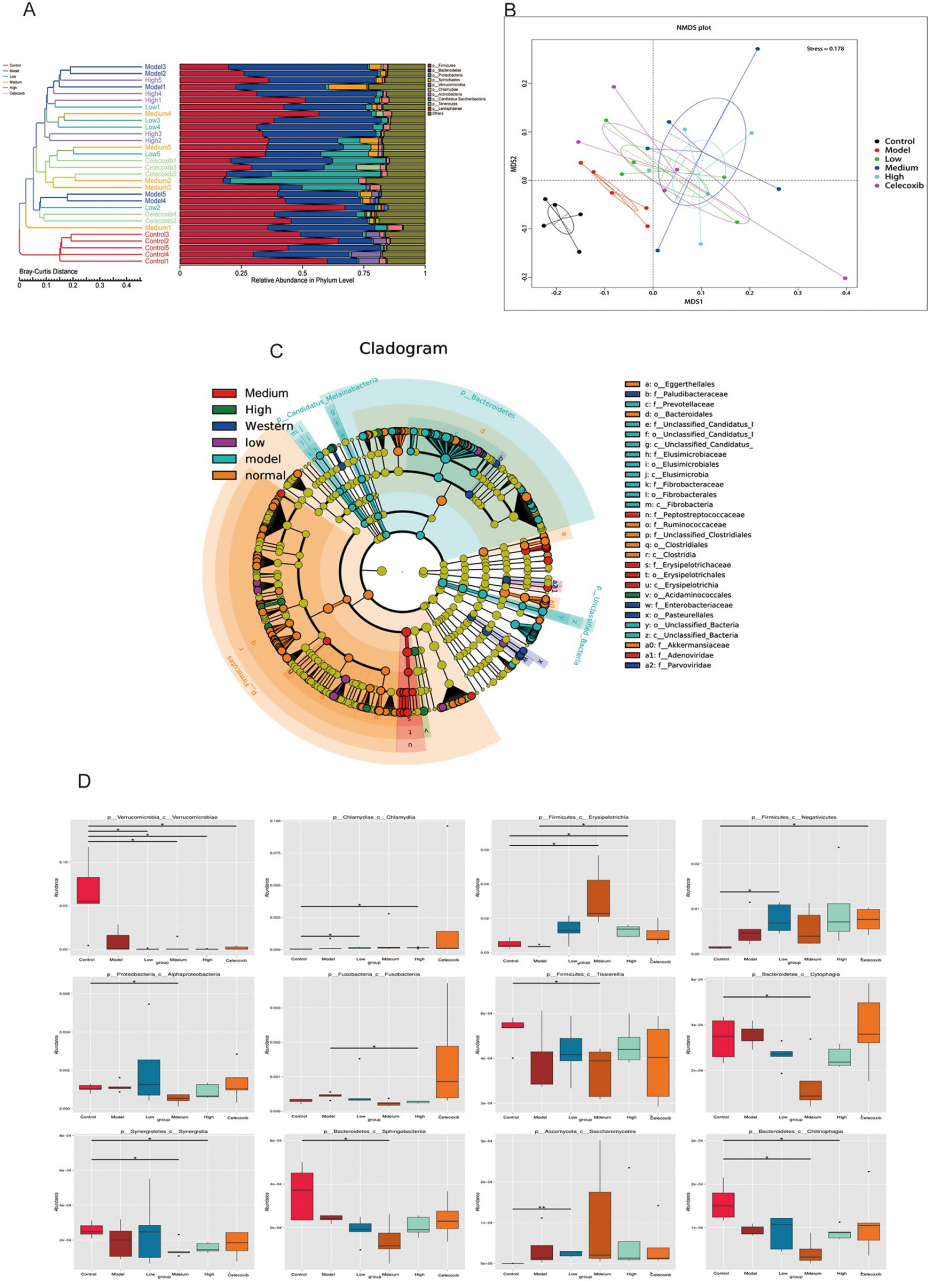
In-depth analysis of intestinal flora in degenerated disc rats and after DHJSD treatment. A. Bray-Curtis distance clustering tree structure with the distribution of relative species abundance at the phylum level for each sample, a distinct separation of the normal group from the hierarchical cluster tree of the other groups, and distinctions between the normal group and the model and drug groups of the bacteriophage. B. Demonstration of NMDS results for species based on phylum levels, with Stress less than 0.2, indicating some reliability of NMDS analysis. C. Evolutionary branching diagram based on the results of LEfSe analysis of species differing between groups at the class, order and family levels. D. Box plots of the abundance distribution of screened differential species between class level groups.

Inter-sample cluster analysis and integration of relative species abundance at the gate level revealed that there were substantial variations in the flora species among the Control group, the Model group, and the drug-treated group (Fig 4A). Further Phylum level species NMDS analysis revealed that there were significant differences in the type of *Bacterial flora* species among these groups (Fig 4B). The coordinates constructed between the Control, Model, and drug-treated groups (containing DHJSD and Celecoxib groups) were clustered in different dimensional spaces, indicating significant differences in flora species among these groups. The DHJSD medium dose and western medication groups’ built coordinates were widely scattered, indicating that these two groups’ intra-group flora species were more dissimilar. The data showed that the Control group, Model group, DHJSD Low group, and Celecoxib group created ellipses in the same plane, indicating that the pattern of change in the number of flora in these groups was the same. After IVDD modeling, the intestinal flora species of rats were considerably different from the Control group, although the pattern of change in flora number was small. Following DHJSD and Celecoxib therapy, differences in intestinal flora species decreased as compared to the Model group, with the differences between the DHJSD Low group and the Celecoxib group being the smallest (S5 Table in S4 File).

#### 3.3 Significantly different species analysis

The evolutionary branching map revealed that the species that varied between groups were mostly clustered in the Control, Model, DHJSD Medium groups (Fig 4C). The intergroup analysis showed that the marker flora of the Control group at the class, order, family level were mainly the two major groups of *Bacillus* and *Clostridium*. And at the level of order began to appear the *Eggerthella; Ruminococca*, *Akkermansiac* appeared at the level of family. In the Model group than the Control group, the difference species at the level of class, order, family were mainly *fibrobacteria*, and at the level of order, family began to appear the *Elusimicrobia*; *Prevotella* appeared at the level of family; in the DHJSD Medium group than the Model group, the difference species at the level of class, order, family were mainly *Eyrsipelotricha*, and at the level of family appeared *Peptostreptococca* and adenovirus. The results of the evolutionary branching plots were comparable to the box plots of abundance distribution of different species within class level groupings based on the Metastats method analysis (Fig 4D). By altering the ecology of Clostridium fibrillarum, Clostridium perfringens, Prevotella, and other bacteria, IVDD induced dysregulation of the colonic microbiota in rats, according to the current gut microbial macrogenetic study (S6 Table in S4 File). These flora were modulated by DHJSD for the treatment of IVDD.

#### 3.4 KEGG annotated gene analysis of biometabolic pathways

**Fig 5:**
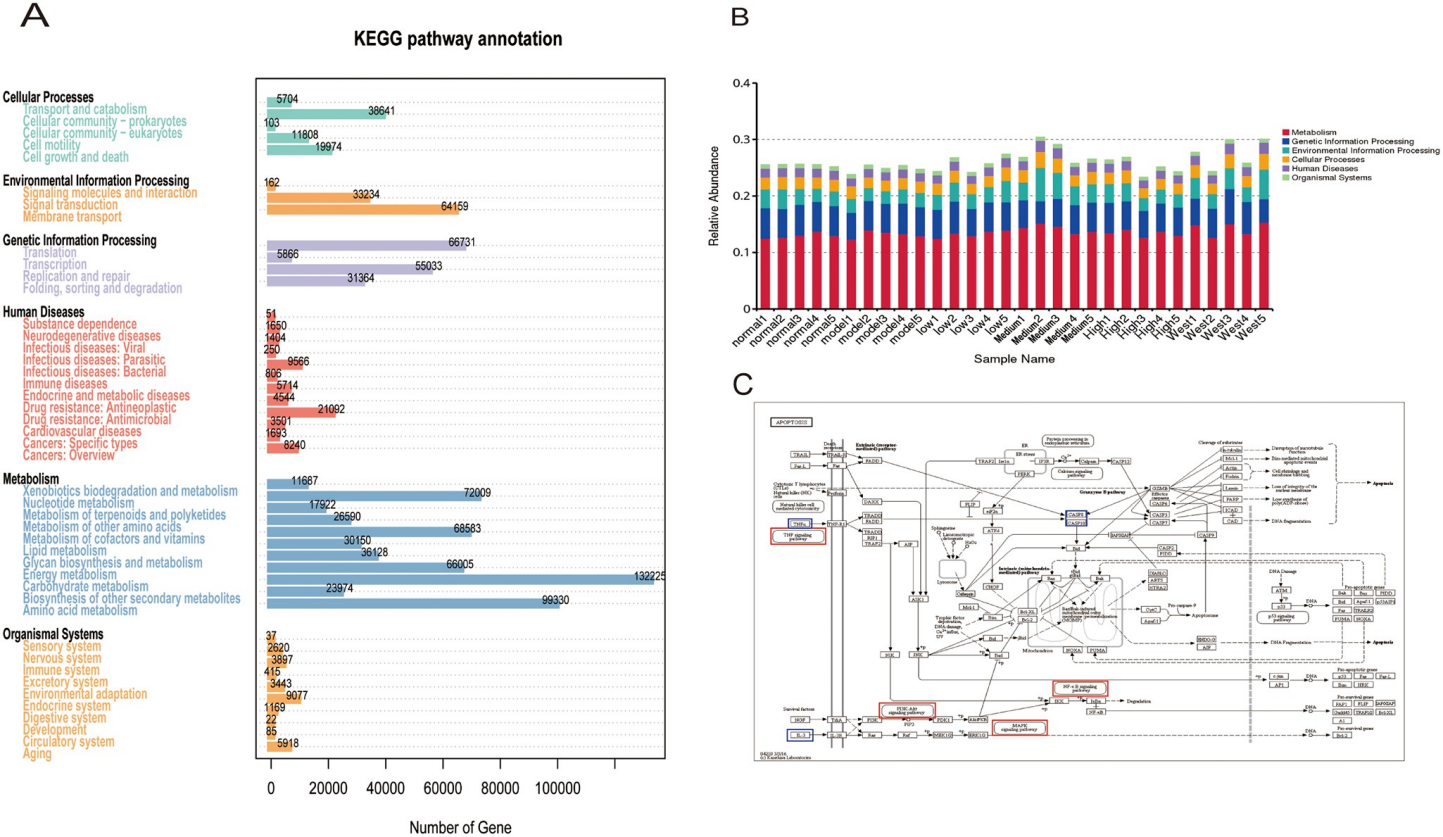
Relevant metabolic pathways involved in intestinal flora in degenerated disc rats and after DHJSD treatment. A. Statistical plot of the number of annotated genes in the KEGG database; B. Bar chart of the relative abundance of functional annotations on level1; C. Pathways related to apoptotic pathways, map04210, the pathway map can reveal that the TNF-a,IL-3,casp8 protein abnormal expression may play an important role in the process of intervertebral disc apoptosis.

Analysis of biometabolic pathway was discovered that the gut flora genes contained the largest relative abundance of gene functions ascribed to metabolism, at roughly 58.31%, showing that the gut microbiota is primarily intimately tied to material metabolism (Fig 5A). The most prevalent component of the gut microbial functions, accounting for 13.18% and 9.90%, respectively, of the total number of genes, was for the metabolism of carbohydrates and amino acids (Fig 5B, S7 Table in S4 File). We discovered that it is primarily closely related to glycine, serine, and threonine metabolism, the citrate cycle, arginine and lysine biosynthesis, methane metabolism, coenzyme B biosynthesis, valine, leucine, and isoleucine biosynthesis, and thioglucoside biosynthesis when it comes to metabolism. The key metabolic pathways engaged in the third level of metabolism, according to further investigation of metabolism and cellular activities, include 2-oxocarboxylic acid metabolism (map01210), amino acid biosynthesis (map01230), and antibiotic biosynthesis (map01130). Apoptotic pathways, which are primarily involved in the third level of cellular activities, include map04210 (Fig 5C), map04214, map04215, and map04210 (Table 3). The major proteins were CASP8, TNF, and IL-3, and the apoptotic signaling pathway annotation mainly involved the expression of TNF signaling pathway, NF-kappa B signaling pathway, PI3K-Akt signaling network, and MAPK signaling pathway (Supplementary material pathway maps. Report in S1 File).

**Table 3:** The annotated pathways for mPATH analysis.

Pathway ID	Pathway Level1	Pathway Level2	Pathway Level3
map01210	Metabolism	Overview	2-Oxocarboxylic acid metabolism
map01230	Metabolism	Overview	Biosynthesis of amino acids
map01130	Metabolism	Overview	Biosynthesis of antibiotics
map04210	Cellular Processes	Cell growth and death	Apoptosis
map04214	Cellular Processes	Cell growth and death	Apoptosis
map04215	Cellular Processes	Cell growth and death	Apoptosis
map04210	Cellular Processes	Cell growth and death	Apoptosis- multiple species

#### 3.5 Related protein validation

**Fig 6:**
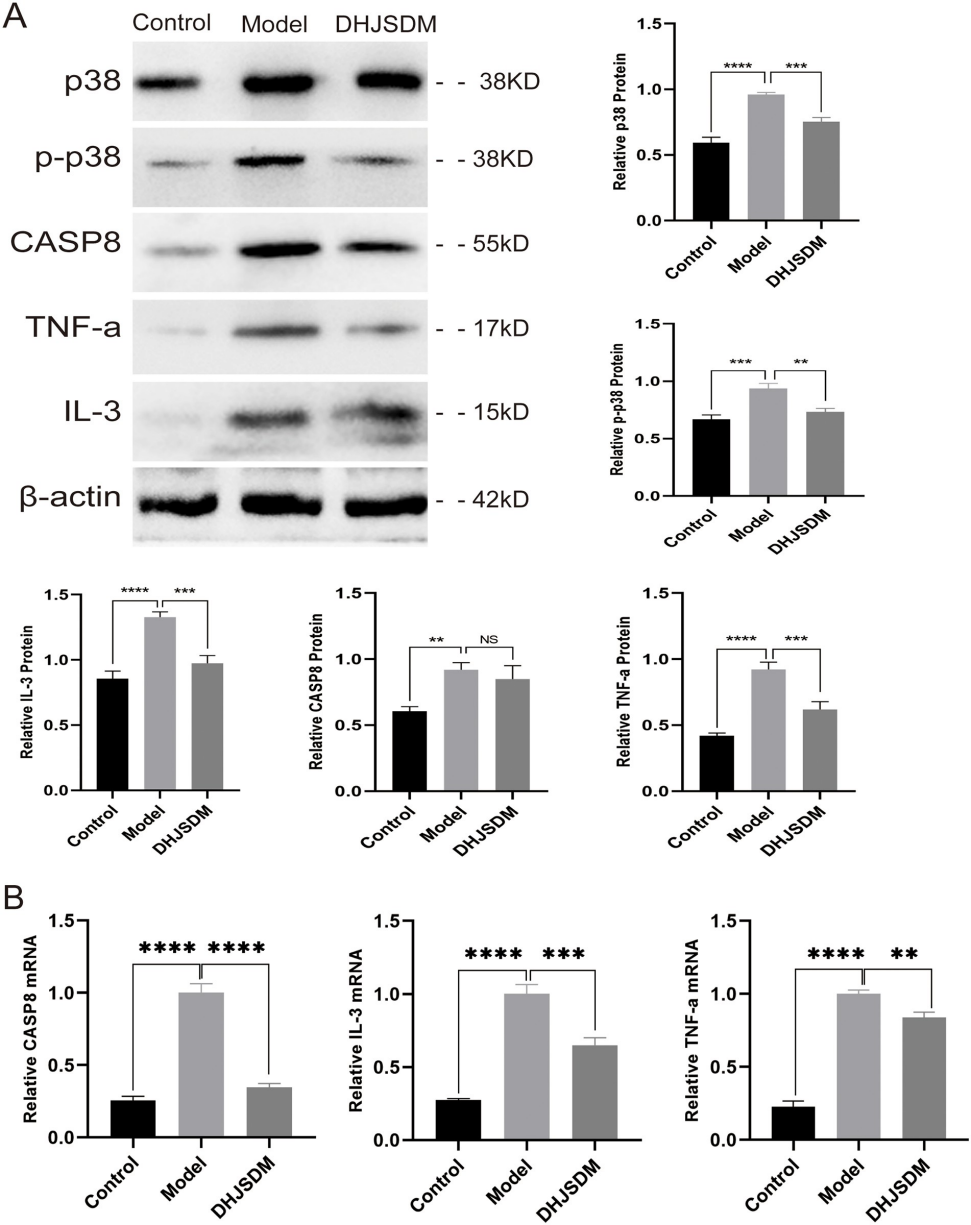
Validation of key proteins of apoptosis pathway based on intestinal flora screening. A. Expression analysis of CASP8, TNF-α and IL-3 proteins in model group and DHJSD medium dose group after intervention based on important proteins screened by apoptotic pathway; B. Expression analysis of CASP8, TNF-α and IL-3 mRAN content in model group and DHJSD medium dose group after intervention based on important proteins screened by apoptotic pathway.

The analysis revealed through macrogenetic research of intestinal flora that DHJSD may modulate TNF, NF-kappa B, PI3K-Akt, MAPK signaling pathways through CASP8, TNF-α, IL-3, and other crucial proteins to inhibit apoptosis in the nucleus pulposus and treat rat intervertebral disc degeneration. Western blot and RT-qPCR assays were used to analyze the tissue samples from the normal group, model group, and DHJSD medium dose group (S12 Table in S4 File). In the intervertebral disc tissues of the rats in the model group, as compared to those in the normal group, the expression levels of CASP8, TNF-α, and IL-3 proteins were significantly upregulated. This was accompanied by the activation of key MAPK signaling pathway proteins P38, MAPK, and P-P38. Following DHJSD treatment, the expression of MAPK signaling pathway was downregulated while the expression levels of CASP8, TNF-α, and IL-3 proteins were downregulated (Fig 6).

### 4 Analysis of rat serum metabolic profiles

#### 4.1 Rat serum metabolite differential analysis

**Fig 7:**
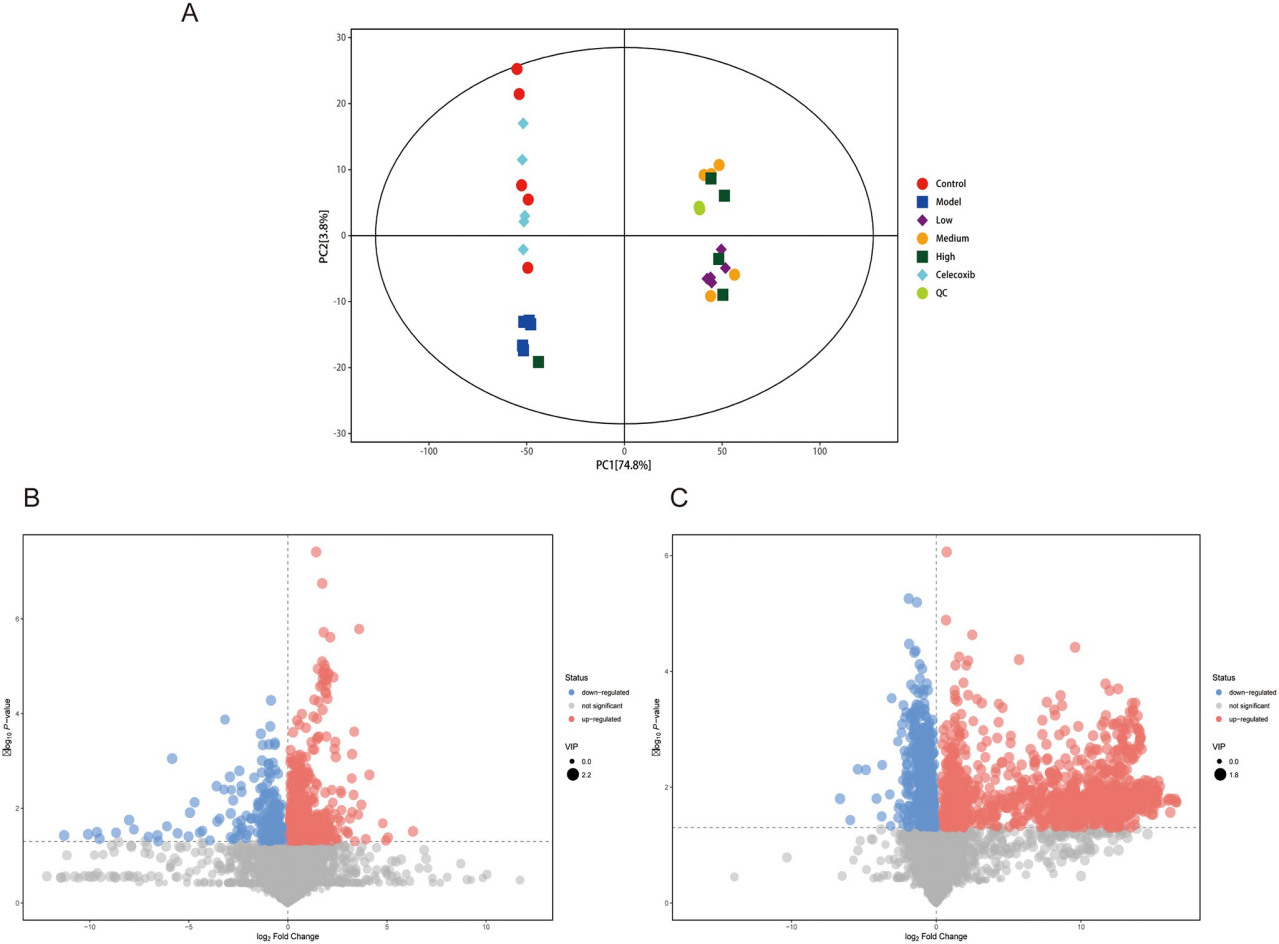
Serum metabolite analysis in degenerated disc rats and after DHJSD treatment. A. Scatter plot of PCA scores for all samples (including QC samples); B. Volcano plot of differential metabolite screening in the model group versus the normal group; D. Volcano plot of differential metabolite screening in the mid-dose group versus the model group; C. Volcano plot of differential metabolite screening in the mid-dose group versus the normal group.

**Fig 8:**
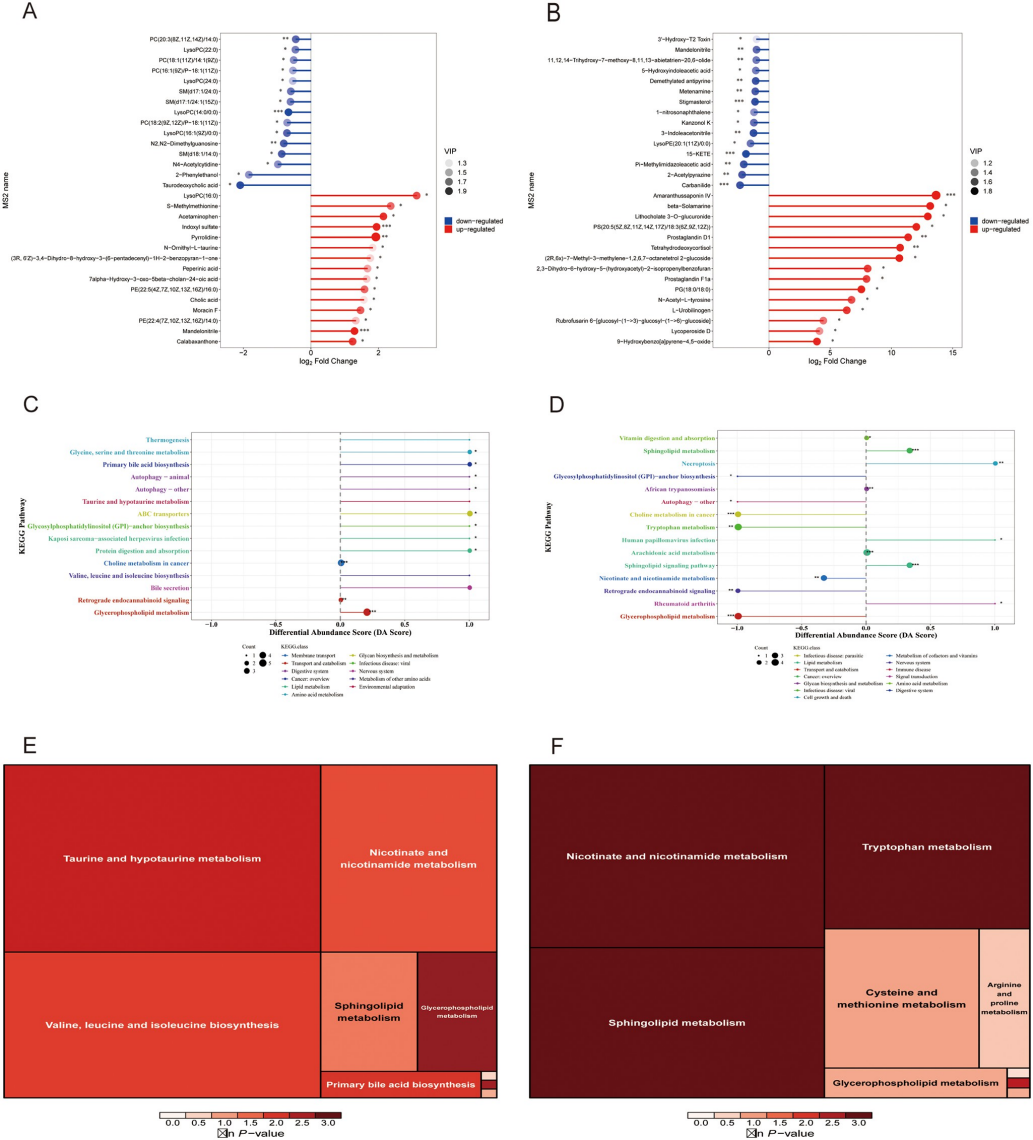
Analysis of metabolic pathways related to serum metabolites in degenerated disc rats and after DHJSD treatment. Matchstick plot of model group vs. normal group; B. Matchstick plot of medium-dose group vs. model group; C. Differential abundance score plot of model group vs. normal group; D. Differential abundance score plot of medium-dose group vs. model group; E. Rectangular tree plot of pathway analysis of model group vs. normal group; F. Rectangular tree plot of pathway analysis of medium-dose group vs. model group. Each square in the rectangular tree represents a metabolic pathway, and the size of the square indicates the influence factor of the pathway in the topological analysis, the larger the square the larger the influence factor; the color of the square indicates the P value of the enrichment analysis, the darker the color the smaller the P value, the more significant the enrichment.

The liquid chromatography-mass spectrometry (LC-MS) technology was employed to detect the serum metabolic profiles of each group of rats, and 304 compounds (negative ion mode) and 560 substances (positive ion mode) were discovered, with the positive ion mode being more valuable for analysis (Table 4). The Total Ion Current (TIC) overlay plot reveals that the mass spectrometry performance is stable and meets the analytical requirements of metabolomics (S1 Fig in S1 File). The aggregation of QC samples in the 2D PCA score plot is good, showing high technique stability (S2 Fig in S1 File). The type and content of the metabolites within each group were quite comparable, and the intra-group differences were minimal, according to multivariate statistical principal component analysis (Fig 7A) [27]. All serum metabolic profiles were different between the groups, and the model and DHJSD groups had a substantial difference in the kinds and amounts of metabolites compared to the normal group, whereas the differences in the western medication group were less pronounced. The normal, model, and DHJSD mid-dose groups were chosen for serum metabolite analysis based on the DR, HE, and intestinal flora data. There were substantial variations in the serum metabolic profiles between the normal, model, and DHJSD mid-dose groups, as shown by the volcano plots of differential metabolites (Fig 7B and 7C), and a total of 2557 differential metabolites were found in the three groups. 847 substantially changed blood metabolic indicators were found in the Model group when compared to the normal group, while 1710 were found in the DHJSD Medium group when compared to the model group (S8 Table in S4 File). For each set of comparisons, selected the top 15 up- and down-regulation multiples of the change for each for matchstick plot presentation. The results showed that the metabolites upregulated in the Model group compared to the Control group were *Mandelonitrile*, *PE (22*:*4(7Z*,*10Z*,*13Z*,*16Z)/14*:*0)*, *Moracin F*, *Cholic acid and Indoxyl sulfate*, significantly up-regulated (***p<0.001) metabolites were *Mandelonitrile*, *Indoxyl sulfate*, and down-regulated products were *Taurodeoxycholic acid*, *N2*,*N2-Dimethylguanosine*, *LysoPC(16*:*1(9Z)/0*:*0)*, and significantly down-regulated (***p<0.001) metabolites were *LysoPC(14*:*0/0*:*0)* (S9 Table in S4 File) (Fig 8A). In comparison with the Model group, the metabolites upregulated after treatment with medium-dose DHJSD were *Lycoperoside D*, *Tetrahydrodeoxycortisol*, *Prostaglandin D1*, *beta-Solamarine*, *Amaranthussaponin IV*, significantly up-regulated metabolites were *Amaranthussaponin IV*, and down-regulated products were *Carbanilide*, *2-Acetylpyrazine*, *Pi- Methylimidazoleacetic acid*, *15-KETE*, *3-Indoleacetonitrile*, *Stigmasterol*, *Metenamine*, *Demethylated antipyrine*, *Mandelonitrile*, and significantly down-regulated metabolites were *15-KET*, *stigmasterol*, and *Carbanilide* (Fig 8B). We examined the major metabolites during disc degeneration as *Mandelonitrile*, *Indoxyl sulfate*, *and LysoPC(14*:*0/0*:*0)*, and the key metabolites that changed after DHJSD treatment as *Amaranthussaponin IV*, *15-KET*, *Stigmasterol*, and *Carbanilide*.

**Table 4:** Total ion counts and identification results of metabolomics samples.

model	total number of ions	MS2
Positive ion mode	8887	560
Negative ion mode	10514	304

#### 4.2 Analysis of metabolic pathways of rat serum metabolic profile differentials

Based on KEGG enrichment pathway analysis, the DA Score demonstrated a trend of multiple upregulation of distinct metabolites of relevant metabolic pathways in the model group compared to normal, and the metabolic pathways with statistical differences were glycine, serine, and threonine metabolism, primary bile acid biosynthesis, autophagy-animal, glycosylphosphatidylinositol (GPI)-anchor biosynthesis, Protein digestion and absorption, Choline metabolism in cancer, Glycerophospholipid metabolism. Choline metabolism in cancer and glycerophospholipid metabolism are metabolic processes that differ significantly (Fig 8C). In the mid-dose group compared to the model group, there were five pathways dominated by metabolite upregulation, namely sphingolipid metabolism, necroptosis, human papillomavirus infection, sphingolipid signaling pathways, rheumatoid arthritis. And Sphingolipid metabolism, necroptosis, and sphingolipid signaling pathways were the ones that displayed statistically significant changes. And seven pathways dominated by metabolite downregulation, namely glycosylphosphatidylinositol (GPI)-anchor biosynthesis, autophagy-other, choline metabolism in cancer, tryptophan metabolism, nicotinic acid and nicotinamide metabolism, retrograde endogenous cannabinoid signaling, glycerophospholipid metabolism. The pathways with statistically significant differences were choline metabolism in cancer, lipid metabolism, tryptophan metabolism, nicotinic acid and nicotinamide metabolism, retrograde endogenous cannabinoid signaling, and glycerophospholipid metabolism (Fig 8D). Overall, the results showed that DHJSD treatment of intervertebral discs promotes upregulation of metabolites related to sphingolipid metabolism, necroptosis, and sphingolipid signaling pathway, and downregulation of metabolites related to choline metabolism in cancer. The process of intervertebral disc degeneration primarily causes upregulation of metabolites in the glycerophospholipid metabolic pathway.

Finally, we identified a total of 59 metabolic pathways related to metabolites in the model and mid-dose groups, as well as a total of 37 metabolic pathways related to differential metabolites in the model and normal groups (S10 Table in S4 File). There were 69 significant metabolic pathways after combining the information from the three metabolic pathway groups and eliminating redundant pathways. We finally screened out 12 important metabolic pathways (Fig 8E and 8F, S11 Table in S4 File), which include amino acid biosynthesis, ABC transporters, protein digestion and absorption, cofactor biosynthesis, bile secretion, glycerophospholipid metabolism, amino acid tRNA biosynthesis, glycine, serine, and threonine metabolism, 2-oxocarboxylic acid metabolism, and metabolic pathways. The aforementioned 12 metabolic pathways are thought to be the key ones via which DHJSD may affect the serum metabolic profile and metabolic pathways for treating intervertebral disc degeneration in rats.

#### 4.3 Correlation analysis of intestinal flora and metabolites

**Fig 9:**
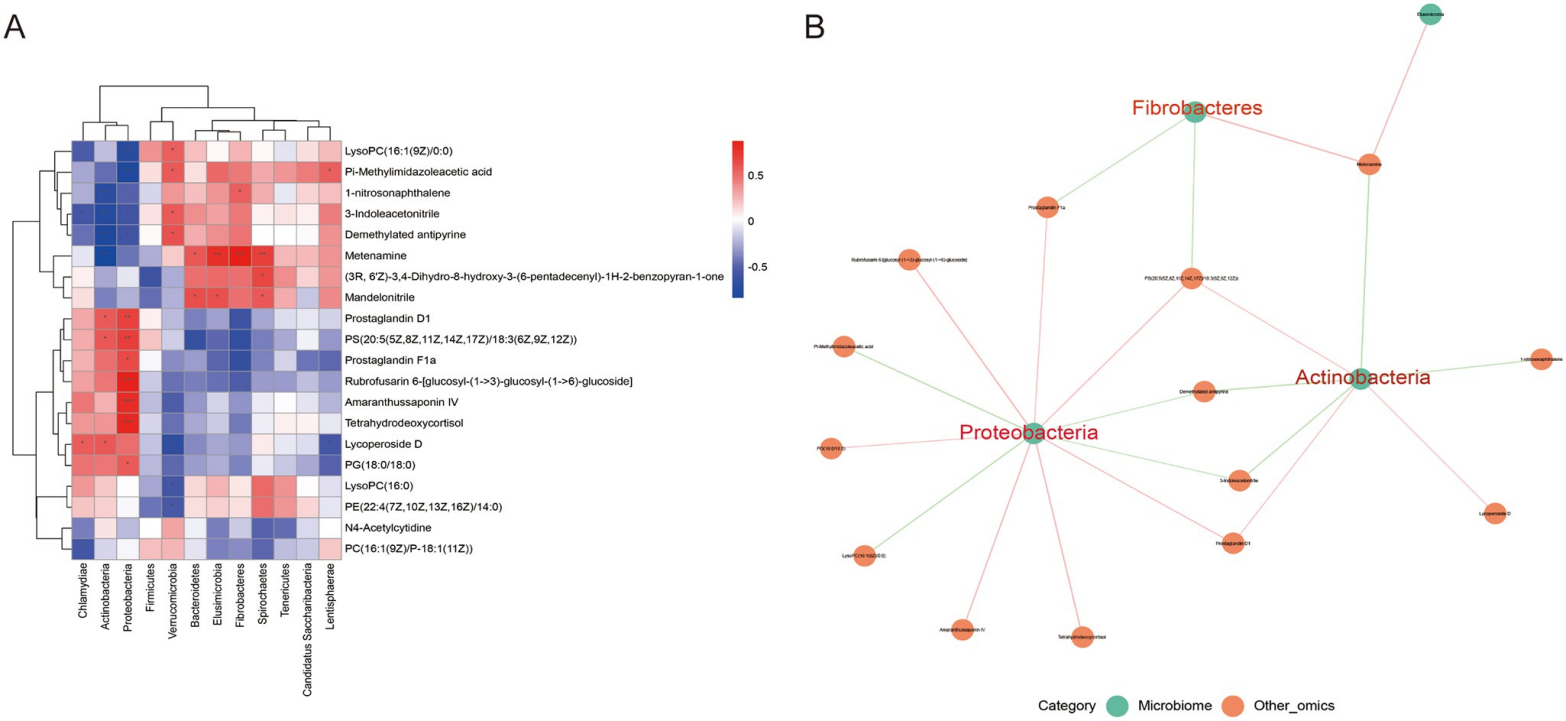
Correlation analysis of characteristic bacteria and metabolites. Correlation analysis of gate-level associated flora with key metabolites in degenerated disc rats and after DHJSD treatment, red indicates positive correlation and blue indicates negative correlation. B. Network diagram of association between intestinal flora and metabolites.

The association between considerably variable tract flora at the Phylum level abundance and significantly altering metabolite levels was examined using the spearman method to further investigate the relationship between tract flora and metabolites during disc degeneration, DHJSD treatment (S13 Table in S4 File). The correlation results were also displayed using a correlation heat map and a correlation network diagram. The correlation heat map’s red and blue colors denote positive and negative correlation, respectively. Fig 9A shows that Actinobacteria were positively correlated with the following metabolites: *Prostaglandin D1*, *PS (20*:*5 (5Z*,*8Z*,*11Z*,*14Z*,*17Z)/18*:*3(6Z*,*9Z*,*12Z))*, *Lycoperoside D*, and negatively correlated with the following metabolites: *Metenamine*, *Demethylated antipyrine*, *1-nitrosonaphthalene*, *3-Indoleacetonitrile*. *Bacteroidetes* were positively correlated with *Mandelonitrile*, *Metenamine*, and negatively correlated with *PS (20*:*5(5Z*,*8Z*,*11Z*, *14Z*,*17Z)/18*:*3(6Z*,*9Z*,*12Z))*. *Chlamydiae* was positively correlated with *Lycoperoside D* and negatively correlated with *3-Indoleacetonitrile*, *PC (16*:*1(9Z)/P-18*:*1(11Z))*. *Fibrobacteres* were positively correlated with *Metenamine*, *1-nitrosonaphthalene and negatively correlated with Prostaglandin D1*, *PS (20*:*5(5Z*,*8Z*,*11Z*,*14Z*,*17Z)/18*:*3 (6Z*,*9Z*,*12Z))*, *Prostaglandin F1a*. *Proteobacteria* were positively correlated with *Rubrofusarin 6-[glucosyl-(1->3)-glucosyl-(1->6)-glucoside]*, *Amaranthussaponin IV*, *Tetrahydrodeoxycortisol*, *Prostaglandin D1*, *PS(20*:*5(5Z*,*8Z*,*11Z*,*14Z*,*17Z)/18*:*3(6Z*,*9Z*,*12Z))*, *Prostaglandin F1a*,*PG(18*:*0/18*:*0)*, and negatively correlated with *LysoPC(16*:*1(9Z)/0*:*0)*, *Pi-Methylimidazoleacetic acid*, *Demethylated antipyrine*, *3-Indoleacetonitrile*. Among them, *Actinobacteria* had statistically significant differences in their negative correlation with metenamine and demethylated *antipyrine* (***P< 0.001). *Fibrobacteres* and *metenamine* had a favorable correlation with statistically significant differences. The differences between Proteobacteria and *Rubrofusarin 6-[glucosyl-(1->3)-glucosyl-(1->6)-glucoside] and Amaranthussaponin IV* were statistically significant (S13 Table in S4 File). *Actinobacteria*, *Fibrobacteria*, *and Proteobacteria* were highly connected with metabolites, according to network plots (Fig 9B), which were consistent with the correlation heat map data.

## Discussion

IVDD will undoubtedly become a common and frequent disease of particular concern today and into the future as the population ages more rapidly [28]. Recent research has revealed that gut flora and metabolite dysregulation may be directly related to IVDD [29]. According to Yinwei et al., dysbiosis of the gut microbiota ecology causes a number of chronic diseases [30]. Additionally, several chronic disorders are linked to inflammatory and metabolic homeostatic abnormalities. The combined metagenomics-metabolomics analysis can be used as a new research tool to investigate the metabolic effects of microbial products and metabolites produced by microorganisms in various organs, forecast the therapeutic potential of focusing on the gut microbiota, and shed light on the pathological mechanisms underlying disease [31]. By controlling metabolic homeostasis and delaying disc degradation, Chen Li et al. discovered that mTORC1 signaling maintains the viability of NP cells [32]. A prior investigation by our group revealed that DHJSD primarily controls the inflammatory response, apoptosis, and extracellular matrix degradation to slow intervertebral disc degeneration [9,33]. Our dose selection is based on the conversion of the dose used by human beings in daily treatment, in which the medium dose is in line with the conventional dose, and for the comparative analysis, half of the medium dose and two times of the low and high doses were set. Therefore, we initially thought that the medium dose was effective, and the results based on Figs 2 and 3 also verified that the medium dose was effective and conformed to the conventional dose used by humans. It has not yet been thoroughly investigated whether DHJSD can treat IVDD by altering gut flora and metabolites.

The development of skeletal diseases is significantly influenced by the gut microbiota [34], which may have an impact on skeletal diseases via three different possible mechanisms, including the regulation of the mucosal and immune systems, control of nutrient absorption in the intestinal epithelium, and translocation of gut microbes through the intestinal endothelial barrier [35]. The *Firmicutes* can encourage polysaccharide fermentation and take part in maintaining intestinal flora homeostasis [36], whereas the *Bacteroidetes* can work on the NF-κB signaling pathway to trigger an inflammatory response [37]. Additionally, there is a strong correlation between intestinal inflammation and a rise in the relative abundance of the *Bacteroidetes* and a fall in the relative abundance of the firmicutes [38]. The intestinal flora may influence the immune system and postpone disc degeneration by creating chemicals with immunomodulatory and anti-inflammatory effects, according to research that links Clostridia to Th cell growth and Treg cell induction within the resident flora [39]. According to our finding, DHJSD can have a positive biological impact by up-regulating the firmicutes, proteobacteria and down-regulating the *bacteroidetes*, enriching the rat intestinal microflora and improving the composition and structure of the bacterial flora.

There may be a connection between *Prevotellaceae* and the inflammatory response in IVDD [40]. *Prevotella* can damage the intestinal mucosal barrier, resulting in intestinal infections and systemic inflammatory reactions when its concentration is high [41]. Firmicutes, which are human probiotics, include *Fibrobacteria* and *Elusimicrobiales*, while Bacteroidetes, which are conditionally harmful bacteria, include *Bacteroidales* and *Prevotellaceae* [42]. The findings of this study demonstrated that DHJSD can slow disc degeneration by altering the microecological balance of the intestine through boosting the abundance of *fibrobacteria* and *elusimicrobia* while reducing the abundance of bacteria that are conditionally pathogenic, like *bacteroides* and *Prevotella*. The intestinal flora of healthy mice also had low levels of the pro-inflammatory cytokines IL-3 and TNF-a, according to some research [43]. Our experimental findings demonstrated that intestinal flora imbalance results in higher levels of pro-inflammatory factors including IL-3 and TNF-a, and that DHJSD treatments can successfully suppress the expression of inflammatory factors. Additionally, we discovered that the apoptotic pathway—of which capse8 is a crucial component—is a crucial mechanism in the process of disc degeneration (Fig 6). Regarding how the gut flora controls this linked protein and signaling system, more proof is required.

In the mammalian gut, there are hundreds of millions of different bacterial communities that interact with their hosts and take part in a variety of physiological processes [44]. *Firmicutes* can encourage the fermentation of polysaccharides, and the efficient consumption of polysaccharides can raise good bacteria and lower bad bacteria, so reestablishing the balance of the gut flora. It may also encourage the synthesis of SCFAs such as propionic acid, butyric acid, and acetic acid [45]. While downregulating inflammatory factors like IL-3 and TNF-a to lessen the inflammatory response, SCFAs maintain the integrity of the intestinal mucosal barrier and control the body’s immunity [46]. The Firmicutes/Bacteroidetes ratio, on the other hand, can be directly impacted by increasing glutamine content to enhance gut microbiology [47]. Our experimental also findings demonstrate that DHJSD can enhance the metabolism of amino acids, carbohydrates, and other intestinal flora metabolic pathways, hence regulating intestinal flora and delaying IVDD.

Modernizing Chinese medicine’s pharmacology through metabolomics results in breakthroughs [48]. Therefore, this study also compared the serum metabolic profile of IVDD rats with normal rats as well as animals that had received DHJSD treatment. It was discovered that IVDD is significantly influenced by changes in metabolites and associated metabolic pathways. Aggregated glycans, which are hydrophilic linker and core proteins with significant amounts of acidic amino acids to preserve the structure and functionality of the intervertebral disc, are produced during amino acid metabolism [49]. Degradation of proteoglycans, which results in the release of neurotransmitters like glutamate into the surrounding milieu and affects ganglion activity and neurotransmission, is strongly linked to intervertebral disc degeneration [50]. In this setting, the degradation of disc proteoglycans during inflammation and disc degeneration is accelerated by glutamate, a final metabolite involved in the metabolism of glutathione, aspartate, and glutamate [51]. Although they are also involved in a number of metabolic processes, other related metabolites such as hypotaurine, taurine, phenylalanine, and threonine have not yet been linked to IVDD or low back pain. Our research demonstrates that potential metabolic indicators of IVDD include serum elevated metabolites such as glutamate, aspartate, glycine, and lactate.

In addition to amino acid metabolism, IVDD rats have altered levels of metabolites in the carbohydrate metabolic pathway. The tricarboxylic acid cycle uses glucose 1-phosphate, which is said to be crucial for the production of energy [50]. We discovered that higher aerobic metabolism and disc pathology-related inflammatory processes are linked to the lower IVDD response to glucose 1-phosphate during pain perception [52]. Besides this, when Shan et al. looked at the plasma levels of metabolites in patients with lumbar disc herniation, they discovered elevated levels of glutamate, aspartate, and glycine as well as decreased glucose 1-phosphate [53]. Radek et alanalysis.’s also revealed that severe disc degeneration was linked to increased concentrations of creatine, glycine, hydroxyproline, alanine, leucine, valine, acetate [54], this generally agrees with our conclusions. Our research also revealed that DHJSD treatment promoted metabolites related to sphingolipid metabolism, necroptosis, and upregulation of the sphingolipid signaling pathway, as well as metabolites related to downregulation of choline metabolism and lipid metabolism in cancer. Finally, disc degeneration caused upregulation of metabolites primarily in the glycerophospholipid metabolic pathway.

**Fig 10:**
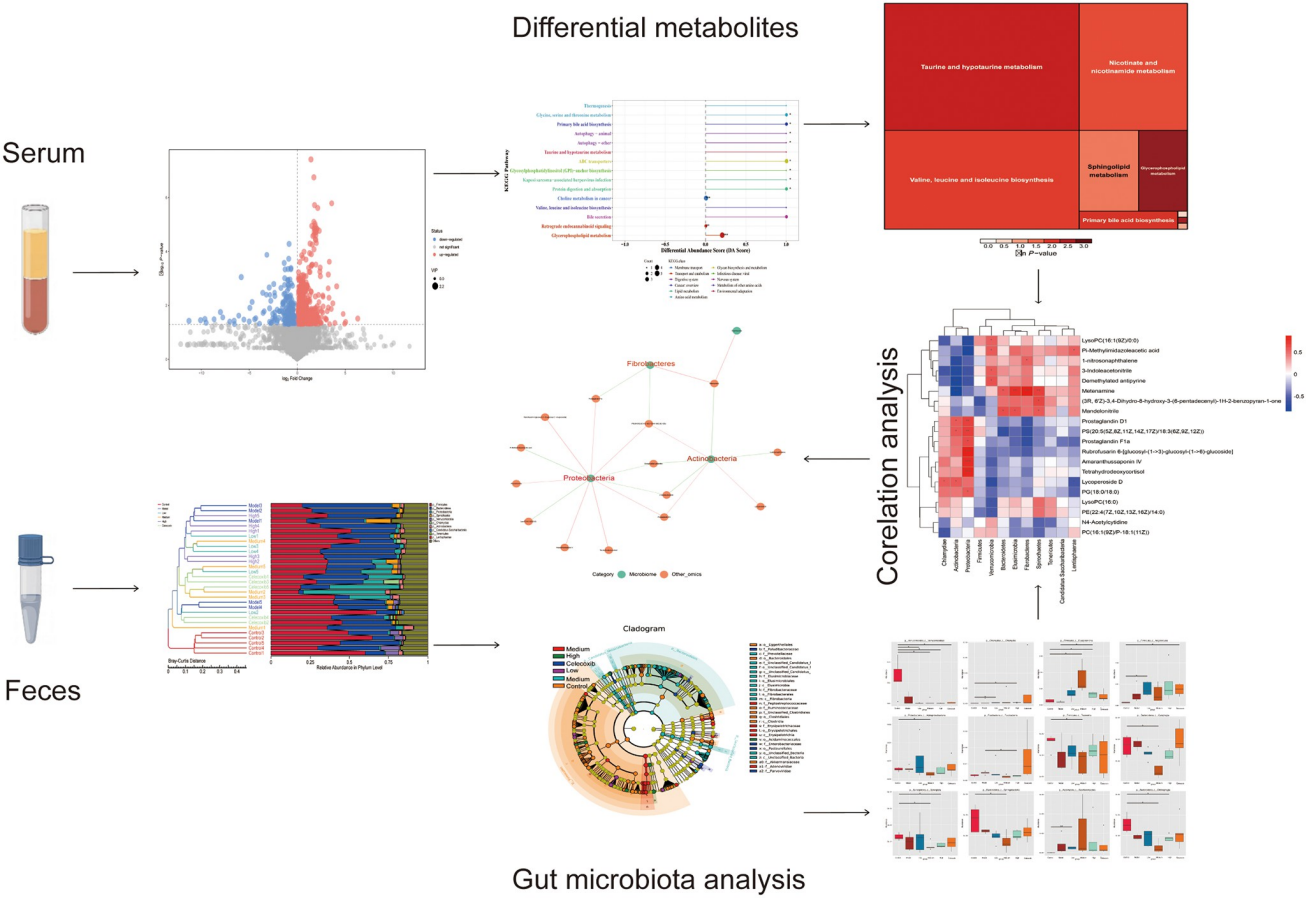
Graphical abstract. Flow chart of macrogenomics and metabolomics investigation of deteriorated disc rats treated with DHJSD.

According to the findings of our experiment, there is a significant correlation between changes in intestinal flora and serum metabolites. On the one hand, we discovered that various flora alterations were positively or adversely linked with metabolite changes. Actinobacteria, Fibrobacteria, Proteobacteria, and Metabolites were among those that have a significant relationship with one another. Additionally, the regulation of intestinal flora and metabolites by metabolic pathways include co-existing metabolic pathways for amino acid biosynthesis, glycine, serine, and threonine metabolism, 2-oxocarboxylic acid metabolism, and metabolism of D-amino acids. So, based on the modulation of these common metabolic pathways, we believe that DHJSD enhances intervertebral discs via altering serum metabolites and gut flora (Fig 10).

## Conclusions

This study shown that IVDD caused alterations in the ecology of *Fibrobacteria*, *Clostridia*, *Elusimicrobiales*, *Prevotella*, etc. as well as a drop in *Firmicutes* and an increase in *Bacteroidetes*. This shift was successfully mitigated by DHJSD intervention. DHJSD therapy following IVDD, the majority of serum metabolites were enriched in metabolic pathways such as amino acid biosynthesis, metabolism of glycine, serine, and threonine, metabolism of 2-oxocarboxylic acids, metabolism of D-amino acids, followed by metabolic pathways, ABC transporters, protein digestion and absorption, biosynthesis of cofactors, bile secretion, metabolism of glycerophospholipids, biosynthesis of amino acid tRNA, and metabolism. Additionally, a strong link between different bacteria and different metabolites was discovered, which may be a crucial regulatory mechanism for the DHJSD treatment of IVDD. These findings provide the theoretical underpinnings for expanding the use of DHJSD in the treatment of IVDD.

## Supporting information

S1 File **Additional Supplementary Fig:** Fig 1: The Total Ion Current (TIC) overlay plot reveals. Fig 2: The aggregation of QC samples in the 2D PCA score plot. **Additional Western Blot:** Original strips and Processing strips. **Additional pathwaymaps.report:** Macrogenome-based analysis of differential pathways across groups.
(ZIP)

S2 File (ZIP)

S3 File (ZIP)

S4 File Additional Supplement. **Table: Statistical calculation**: Source documents for statistical analysis involved in the manuscript. **Oligonucleotide primers**: Oligonucleotide primers used in real-time PCR. Supplement—Table S1: Raw data for different groups of DHI values. Supplement—Table S2: Raw data for different groups of staining scores. Supplement—Table S3: Statistics of the number of genes in each sample. Supplement—Table S4: the differential abundance statistics of phylum-level flora. Supplement—Table S5-1: NMDS_scores. Supplement—Table S5-2: table.p10.cluster. Supplement—Table S6: Characteristic flora of different groups. Supplement—Table S7: KEGG pathway annotation. Supplement—Table S8 Normal-Modle: Serum metabolites in different groups. Supplement—Table S8 Medium-Modle: Serum metabolites in different groups. Supplement—Table S9: The top 15 up- and down-regulated metabolites in the different groups of serum metabolite changes. Supplement—Table S10: Important metabolic pathways for different groups of serum metabolites. Supplement—Table S11: Key metabolic pathways. Supplement—Table S12: The genes encoding CASP8, TNF-α, and IL-3’s final list of primer sequences was acquired. Supplement—Table S13: The association between considerably variable tract flora at the Phylum level abundance and significantly altering metabolite levels. Supplement—Table S14: Mass spectrometry data of major components of DHJSD.
(ZIP)

## Abbreviations

DHI: disc height index
DHJSD: Duhuo Jisheng Decoction
HE: hematoxylin-eosin
IVDD: intervertebral degenerative disc degeneration
LBP: low back pain
SCFAs: short-chain amino acids
SOFG: Safranin O-Fast Green
TCM: Traditional Chinese Medicine
TIC: total ion current

## References

[1] Siccoli A, Staartjes VE, De Wispelaere MP, Vergroesen PA, Schröder ML. Tandem Disc Herniation of the Lumbar and Cervical Spine: Case Series and Review of the Epidemiological, Pathophysiological and Genetic Literature. Cureus. 2019;11(2):e4081. Epub 2019/04/26. doi: 10.7759/cureus.4081 . 31019859 PMC6467429

[2] Dower A, Davies MA, Ghahreman A. Pathologic Basis of Lumbar Radicular Pain. World Neurosurg. 2019;128:114–21. Epub 2019/04/28. doi: 10.1016/j.wneu.2019.04.147 . 31028982

[3] Hartvigsen J, Hancock MJ, Kongsted A, Louw Q, Ferreira ML, Genevay S, et al. What low back pain is and why we need to pay attention. Lancet. 2018;391(10137):2356–67. Epub 2018/03/27. doi: 10.1016/S0140-6736(18)30480-X . 29573870

[4] Wang L, Guo Q, Lu X, Ni B. Surgical versus nonsurgical treatment of chronic low back pain: A meta-analysis based on current evidence. J Back Musculoskelet Rehabil. 2016;29(3):393–401. Epub 2015/09/26. doi: 10.3233/BMR-150632 . 26406211

[5] Maher C, Underwood M, Buchbinder R. Non-specific low back pain. Lancet. 2017;389(10070):736–47. Epub 2016/10/18. doi: 10.1016/S0140-6736(16)30970-9 . 27745712

[6] Zhang B, Xu H, Wang J, Liu B, Sun G. A narrative review of non-operative treatment, especially traditional Chinese medicine therapy, for lumbar intervertebral disc herniation. Biosci Trends. 2017;11(4):406–17. Epub 2017/09/15. doi: 10.5582/bst.2017.01199 . 28904328

[7] Sun K, Zhu LG, Wei X, Yin H, Zhan JW, Yin XL, et al. [Research progress in mechanism of Chinese herbal compounds and monomers in delaying lumbar intervertebral disc degeneration]. Zhongguo Zhong Yao Za Zhi. 2022;47(9):2400–8. Epub 2022/05/10. doi: 10.19540/j.cnki.cjcmm.20211020.401 . 35531687

[8] Zhao J, Liang G, Pan J, Yang W, Zeng L, Liu J. Efficacy of Duhuo Jisheng Decoction for Treating Cold-Dampness Obstruction Syndrome-Type Knee Osteoarthritis: A Pooled Analysis. Biomed Res Int. 2022;2022:2350404. Epub 2022/07/02. doi: 10.1155/2022/2350404 . 35774274 PMC9239816

[9] Liu ZC, Wang ZL, Huang CY, Fu ZJ, Liu Y, Wei ZC, et al. Duhuo Jisheng Decoction inhibits SDF-1-induced inflammation and matrix degradation in human degenerative nucleus pulposus cells in vitro through the CXCR4/NF-κB pathway. Acta Pharmacol Sin. 2018;39(6):912–22. Epub 2018/05/26. doi: 10.1038/aps.2018.36 . 29795361 PMC6256264

[10] Zhou D, Song C, Mei Y, Cheng K, Liu F, Cai W, et al. A review of Duhuo Jisheng decoction mechanisms in intervertebral disc degeneration in vitro and animal studies. J Orthop Surg Res. 2023;18(1):436. Epub 20230616. doi: 10.1186/s13018-023-03869-4 . 37322524 PMC10273736

[11] Wang YH, Zhou Y, Gao X, Sun S, Xie YZ, Hu YP, et al. Duhuo Jisheng Decoction regulates intracellular zinc homeostasis by enhancing autophagy via PTEN/Akt/mTOR pathway to improve knee cartilage degeneration. PLoS One. 2024;19(1):e0290925. Epub 20240102. doi: 10.1371/journal.pone.0290925 . 38166086 PMC10760926

[12] Liu ZC, Jiang Y, Huang CY, Liu Y, Wei ZC, Liu SG, et al. [Mechanism of Duhuo Jisheng decotion in delaying degeneration of nucleus pulposus cells in human intervertebral disc]. Zhongguo Zhong Yao Za Zhi. 2018;43(13):2764–9. Epub 2018/08/17. doi: 10.19540/j.cnki.cjcmm.20180503.001 . 30111029

[13] Sun K, Huang F, Qi B, Yin H, Tang B, Yang B, et al. A systematic review and meta-analysis for Chinese herbal medicine Duhuo Jisheng decoction in treatment of lumbar disc herniation: A protocol for a systematic review. Medicine (Baltimore). 2020;99(9):e19310. Epub 2020/03/03. doi: 10.1097/MD.0000000000019310 . 32118755 PMC7478537

[14] Guo D, Cheng K, Song C, Liu F, Cai W, Chen J, et al. Mechanisms of inhibition of nucleus pulposus cells pyroptosis through SDF1/CXCR4-NFkB-NLRP3 axis in the treatment of intervertebral disc degeneration by Duhuo Jisheng Decoction. Int Immunopharmacol. 2023;124(Pt A):110844. Epub 20230828. doi: 10.1016/j.intimp.2023.110844 . 37647678

[15] Liu W, Jin S, Huang M, Li Y, Wang Z, Wang P, et al. Duhuo jisheng decoction suppresses matrix degradation and apoptosis in human nucleus pulposus cells and ameliorates disc degeneration in a rat model. J Ethnopharmacol. 2020;250:112494. Epub 2019/12/25. doi: 10.1016/j.jep.2019.112494 . 31874213

[16] Lu L, Chen X, Liu Y, Yu X. Gut microbiota and bone metabolism. Faseb j. 2021;35(7):e21740. Epub 2021/06/19. doi: 10.1096/fj.202100451R . 34143911

[17] Li J, Ho WTP, Liu C, Chow SK, Ip M, Yu J, et al. The role of gut microbiota in bone homeostasis. Bone Joint Res. 2021;10(1):51–9. Epub 2021/01/16. doi: 10.1302/2046-3758.101.BJR-2020-0273.R1 . 33448869 PMC7845471

[18] Zaiss MM, Jones RM, Schett G, Pacifici R. The gut-bone axis: how bacterial metabolites bridge the distance. J Clin Invest. 2019;129(8):3018–28. Epub 2019/07/16. doi: 10.1172/JCI128521 VSL Pharmaceuticals. 31305265 PMC6668676

[19] Ding K, Hua F, Ding W. Gut Microbiome and Osteoporosis. Aging Dis. 2020;11(2):438–47. Epub 2020/04/08. doi: 10.14336/AD.2019.0523 . 32257552 PMC7069453

[20] Qian J, Ge J, Yan Q, Wu C, Yang H, Zou J. Selection of the Optimal Puncture Needle for Induction of a Rat Intervertebral Disc Degeneration Model. Pain Physician. 2019;22(4):353–60. Epub 2019/07/25. . 31337166

[21] Cuperlovic-Culf M, Culf AS. Applied metabolomics in drug discovery. Expert Opin Drug Discov. 2016;11(8):759–70. Epub 2016/07/02. doi: 10.1080/17460441.2016.1195365 . 27366968

[22] Raes J, Foerstner KU, Bork P. Get the most out of your metagenome: computational analysis of environmental sequence data. Curr Opin Microbiol. 2007;10(5):490–8. Epub 2007/10/16. doi: 10.1016/j.mib.2007.09.001 . 17936679

[23] Inoue H, Ohmori K, Miyasaka K, Hosoe H. Radiographic evaluation of the lumbosacral disc height. Skeletal Radiol. 1999;28(11):638–43. Epub 1999/12/11. doi: 10.1007/s002560050566 . 10591927

[24] Zeller G, Tap J, Voigt AY, Sunagawa S, Kultima JR, Costea PI, et al. Potential of fecal microbiota for early-stage detection of colorectal cancer. Mol Syst Biol. 2014;10(11):766. Epub 2014/11/30. doi: 10.15252/msb.20145645 . 25432777 PMC4299606

[25] Zhang Z, Zhai L, Lu J, Sun S, Wang D, Zhao D, et al. Shen-Hong-Tong-Luo Formula Attenuates Macrophage Inflammation and Lipid Accumulation through the Activation of the PPAR-γ/LXR-α/ABCA1 Pathway. Oxid Med Cell Longev. 2020;2020:3426925. Epub 2020/10/22. doi: 10.1155/2020/3426925 . 33082908 PMC7556105

[26] Ponce MC, Zorzi AR, Miranda JB, Amstalden EMI. Proposal for a New Histological Scoring System for Cartilage Repair. Clinics (Sao Paulo). 2018;73:e562. Epub 2018/12/06. doi: 10.6061/clinics/2018/e562 . 30517286 PMC6238816

[27] Bylesjö M, Eriksson D, Sjödin A, Jansson S, Moritz T, Trygg J. Orthogonal projections to latent structures as a strategy for microarray data normalization. BMC Bioinformatics. 2007;8:207. Epub 2007/06/20. doi: 10.1186/1471-2105-8-207 . 17577396 PMC1906839

[28] Adams MA, Roughley PJ. What is intervertebral disc degeneration, and what causes it? Spine (Phila Pa 1976). 2006;31(18):2151–61. Epub 2006/08/18. doi: 10.1097/01.brs.0000231761.73859.2c . 16915105

[29] Cunha C, Silva AJ, Pereira P, Vaz R, Gonçalves RM, Barbosa MA. The inflammatory response in the regression of lumbar disc herniation. Arthritis Res Ther. 2018;20(1):251. Epub 2018/11/08. doi: 10.1186/s13075-018-1743-4 . 30400975 PMC6235196

[30] Chen Y, Zhou J, Wang L. Role and Mechanism of Gut Microbiota in Human Disease. Front Cell Infect Microbiol. 2021;11:625913. Epub 2021/04/06. doi: 10.3389/fcimb.2021.625913 . 33816335 PMC8010197

[31] Olofsson LE, Bäckhed F. The Metabolic Role and Therapeutic Potential of the Microbiome. Endocr Rev. 2022. Epub 2022/01/31. doi: 10.1210/endrev/bnac004 . 35094076 PMC9512151

[32] Chen R, Yang F, Wang Y, Wang X, Fan X. Pharmacological inhibition of mTORC1 activity protects against inflammation-induced apoptosis of nucleus pulposus cells. Braz J Med Biol Res. 2021;54(5):e10185. Epub 2021/03/18. doi: 10.1590/1414-431X202010185 . 33729389 PMC7959168

[33] Liu Z, Ma C, Shen J, Wang D, Hao J, Hu Z. SDF‑1/CXCR4 axis induces apoptosis of human degenerative nucleus pulposus cells via the NF‑κB pathway. Mol Med Rep. 2016;14(1):783–9. Epub 2016/05/26. doi: 10.3892/mmr.2016.5341 . 27220474 PMC4918601

[34] Hernandez CJ, Guss JD, Luna M, Goldring SR. Links Between the Microbiome and Bone. J Bone Miner Res. 2016;31(9):1638–46. Epub 2016/06/19. doi: 10.1002/jbmr.2887 . 27317164 PMC5434873

[35] Tu Y, Yang R, Xu X, Zhou X. The microbiota-gut-bone axis and bone health. J Leukoc Biol. 2021;110(3):525–37. Epub 2021/04/23. doi: 10.1002/JLB.3MR0321-755R . 33884666

[36] Porter NT, Martens EC. The Critical Roles of Polysaccharides in Gut Microbial Ecology and Physiology. Annu Rev Microbiol. 2017;71:349–69. Epub 2017/06/29. doi: 10.1146/annurev-micro-102215-095316 . 28657886

[37] Kitaura H, Kimura K, Ishida M, Kohara H, Yoshimatsu M, Takano-Yamamoto T. Immunological reaction in TNF-α-mediated osteoclast formation and bone resorption in vitro and in vivo. Clin Dev Immunol. 2013;2013:181849. Epub 2013/06/14. doi: 10.1155/2013/181849 . 23762085 PMC3676982

[38] Zhang X, Zhang D, Jia H, Feng Q, Wang D, Liang D, et al. The oral and gut microbiomes are perturbed in rheumatoid arthritis and partly normalized after treatment. Nat Med. 2015;21(8):895–905. Epub 2015/07/28. doi: 10.1038/nm.3914 . 26214836

[39] Maeda Y, Kurakawa T, Umemoto E, Motooka D, Ito Y, Gotoh K, et al. Dysbiosis Contributes to Arthritis Development via Activation of Autoreactive T Cells in the Intestine. Arthritis Rheumatol. 2016;68(11):2646–61. Epub 2016/10/28. doi: 10.1002/art.39783 . 27333153

[40] Marietta EV, Murray JA, Luckey DH, Jeraldo PR, Lamba A, Patel R, et al. Suppression of Inflammatory Arthritis by Human Gut-Derived Prevotella histicola in Humanized Mice. Arthritis Rheumatol. 2016;68(12):2878–88. Epub 2016/06/24. doi: 10.1002/art.39785 . 27337150 PMC5125894

[41] Pisipati S, Connor BA, Riddle MS. Updates on the epidemiology, pathogenesis, diagnosis, and management of postinfectious irritable bowel syndrome. Curr Opin Infect Dis. 2020;33(5):411–8. Epub 2020/08/25. doi: 10.1097/QCO.0000000000000666 . 32833689

[42] Carasso S, Fishman B, Lask LS, Shochat T, Geva-Zatorsky N, Tauber E. Metagenomic analysis reveals the signature of gut microbiota associated with human chronotypes. Faseb j. 2021;35(11):e22011. Epub 2021/10/26. doi: 10.1096/fj.202100857RR . 34695305

[43] Sjögren K, Engdahl C, Henning P, Lerner UH, Tremaroli V, Lagerquist MK, et al. The gut microbiota regulates bone mass in mice. J Bone Miner Res. 2012;27(6):1357–67. Epub 2012/03/13. doi: 10.1002/jbmr.1588 . 22407806 PMC3415623

[44] Costa MC, Weese JS. Understanding the Intestinal Microbiome in Health and Disease. Vet Clin North Am Equine Pract. 2018;34(1):1–12. Epub 2018/02/07. doi: 10.1016/j.cveq.2017.11.005 . 29402480

[45] AlEssa HB, Cohen R, Malik VS, Adebamowo SN, Rimm EB, Manson JE, et al. Carbohydrate quality and quantity and risk of coronary heart disease among US women and men. Am J Clin Nutr. 2018;107(2):257–67. Epub 2018/03/13. doi: 10.1093/ajcn/nqx060 . 29529162 PMC6454480

[46] Parada Venegas D, De la Fuente MK, Landskron G, González MJ, Quera R, Dijkstra G, et al. Short Chain Fatty Acids (SCFAs)-Mediated Gut Epithelial and Immune Regulation and Its Relevance for Inflammatory Bowel Diseases. Front Immunol. 2019;10:277. Epub 2019/03/28. doi: 10.3389/fimmu.2019.00277 . 30915065 PMC6421268

[47] de Souza AZ, Zambom AZ, Abboud KY, Reis SK, Tannihão F, Guadagnini D, et al. Oral supplementation with L-glutamine alters gut microbiota of obese and overweight adults: A pilot study. Nutrition. 2015;31(6):884–9. Epub 2015/05/03. doi: 10.1016/j.nut.2015.01.004 . 25933498

[48] He Y, Gao T, Li J, Chen Z, Wang L, Zhang J, et al. Metabonomics study on the effect of Siwu Decoction for blood deficiency syndrome in rats using UPLC-Q/TOF-MS analysis. Biomed Chromatogr. 2019;33(11):e4617. Epub 2019/06/18. doi: 10.1002/bmc.4617 . 31207665

[49] Melrose J, Ghosh P, Taylor TK, Latham J, Moore R. Topographical variation in the catabolism of aggrecan in an ovine annular lesion model of experimental disc degeneration. J Spinal Disord. 1997;10(1):55–67. Epub 1997/02/01. . 9041497

[50] Harrington JF, Messier AA, Bereiter D, Barnes B, Epstein MH. Herniated lumbar disc material as a source of free glutamate available to affect pain signals through the dorsal root ganglion. Spine (Phila Pa 1976). 2000;25(8):929–36. Epub 2000/04/18. doi: 10.1097/00007632-200004150-00006 . 10767804

[51] Bertrand J, Marion-Letellier R, Azhar S, Chan P, Legrand R, Goichon A, et al. Glutamine enema regulates colonic ubiquitinated proteins but not proteasome activities during TNBS-induced colitis leading to increased mitochondrial activity. Proteomics. 2015;15(13):2198–210. Epub 2015/02/18. doi: 10.1002/pmic.201400304 . 25689466

[52] Kudo F, Kawabe K, Kuriki H, Eguchi T, Kakinuma K. A new family of glucose-1-phosphate/glucosamine-1-phosphate nucleotidylyltransferase in the biosynthetic pathways for antibiotics. J Am Chem Soc. 2005;127(6):1711–8. Epub 2005/02/11. doi: 10.1021/ja044921b . 15701005

[53] Shan L, Liao F, Jin H, Ye F, Tong P, Xiao L, et al. Plasma metabonomic profiling of lumbar disc herniation and its traditional Chinese medicine subtypes in patients by using gas chromatography coupled with mass spectrometry. Mol Biosyst. 2014;10(11):2965–73. Epub 2014/08/22. doi: 10.1039/c4mb00301b . 25144444

[54] Radek M, Pacholczyk-Sienicka B, Jankowski S, Albrecht Ł, Grodzka M, Depta A, et al. Assessing the correlation between the degree of disc degeneration on the Pfirrmann scale and the metabolites identified in HR-MAS NMR spectroscopy. Magn Reson Imaging. 2016;34(4):376–80. Epub 2015/12/29. doi: 10.1016/j.mri.2015.12.005 . 26708032

