# Supplementary material for: Integrated metagenomic and metabonomic mechanisms for the therapeutic effects of Duhuo Jisheng decoction on intervertebral disc degeneration
Source: PLoS One. 2024 Oct 17;19(10):e0310014. doi: 10.1371/journal.pone.0310014 (PMC11486403; doi:10.1371/journal.pone.0310014)
Supplement: S1 File — Additional Supplementary Fig: Fig 1: The Total Ion Current (TIC) overlay plot reveals. Fig 2: The aggregation of QC samples in the 2D PCA score plot. Additional Western Blot: Original strips and Processing strips. Additional pathwaymaps.report: Macrogenome-based analysis of differential pathways across groups. (ZIP) [file pone.0310014.s001.zip › 3 supplement.material/pathwaymaps.report/pathway.html]

KEGG Pathway maps

  

# The annotated pathways

for mPATH analysis

| Compare Samples | Strict Link |
| low\_VS\_normal | >please click |
| model\_VS\_normal | >please click |
| High\_VS\_normal | >please click |
| Western\_VS\_normal | >please click |
| Dose\_VS\_normal | >please click |

for other pathways' genenum and EC's MetaStats Analysis

| Pathway ID | Pathway Level1 | Pathway Level2 | Pathway Level3 | Identified ECs | Sign\_diff ECs |
| map04210 | Cellular Processes | Cell growth and death | Apoptosis | 2.7.11.1; 1.-.-.-; 2.3.2.27; 3.-.-.-; 2.7.11.25; 2.4.2.30; 3.4.22.-; 2.7.11.24; 3.4.22.1; 2.7.12.2 |  |
| map04214 | Cellular Processes | Cell growth and death | Apoptosis - fly | 2.7.11.25; 2.7.12.2; 2.7.11.24; 2.3.2.27; 3.4.22.-; 1.11.1.15; 2.7.11.1; 3.1.3.16; 2.3.2.23; 1.-.-.-; 3.6.5.-; 3.6.5.5; 2.4.2.30 |  |
| map04215 | Cellular Processes | Cell growth and death | Apoptosis - multiple species | 3.4.22.-; 2.3.2.27; 2.3.2.23; 2.7.11.24 |  |
