# Supplementary material for: Integrated metagenomic and metabonomic mechanisms for the therapeutic effects of Duhuo Jisheng decoction on intervertebral disc degeneration
Source: PLoS One. 2024 Oct 17;19(10):e0310014. doi: 10.1371/journal.pone.0310014 (PMC11486403; doi:10.1371/journal.pone.0310014)
Supplement: S1 File — Additional Supplementary Fig: Fig 1: The Total Ion Current (TIC) overlay plot reveals. Fig 2: The aggregation of QC samples in the 2D PCA score plot. Additional Western Blot: Original strips and Processing strips. Additional pathwaymaps.report: Macrogenome-based analysis of differential pathways across groups. (ZIP) [file pone.0310014.s001.zip › 3 supplement.material/pathwaymaps.report/samples/Dose_VS_normal.html]

KEGG Pathway maps

  

# The annotated pathways for Dose\_VS\_normal

| Pathway ID | Pathway Level1 | Pathway Level2 | Pathway Level3 |
| --- | --- | --- | --- |
| map01210 | Metabolism | Overview | 2-Oxocarboxylic acid metabolism |
| map01230 | Metabolism | Overview | Biosynthesis of amino acids |
| map01130 | Metabolism | Overview | Biosynthesis of antibiotics |
