# Supplementary material for: Integrated metagenomic and metabonomic mechanisms for the therapeutic effects of Duhuo Jisheng decoction on intervertebral disc degeneration
Source: PLoS One. 2024 Oct 17;19(10):e0310014. doi: 10.1371/journal.pone.0310014 (PMC11486403; doi:10.1371/journal.pone.0310014)
Supplement: S1 File — Additional Supplementary Fig: Fig 1: The Total Ion Current (TIC) overlay plot reveals. Fig 2: The aggregation of QC samples in the 2D PCA score plot. Additional Western Blot: Original strips and Processing strips. Additional pathwaymaps.report: Macrogenome-based analysis of differential pathways across groups. (ZIP) [file pone.0310014.s001.zip › 3 supplement.material/Supplement.Statistical/Oligonucleotide primers.docx]

Oligonucleotide primers used in real-time PCR

| Gene | Forward primer (5′-3′) | Reverse primer (5′-3′) |
| --- | --- | --- |
| CASP8 | CAAGAGGAAATCTCCAAATGCAAAC | CAGGATGTCCAACTTTCCTTCTCC |
| TNF-a | GAGGCCAAGCCCTGGTATG | CGGGCCGATTGATCTCAGC |
| IL-3 | CCTCGGGACATCAAAAACAGC | GAGAACACAACCGCACAAGG |
