# Supplementary material for: Integrated metagenomic and metabonomic mechanisms for the therapeutic effects of Duhuo Jisheng decoction on intervertebral disc degeneration
Source: PLoS One. 2024 Oct 17;19(10):e0310014. doi: 10.1371/journal.pone.0310014 (PMC11486403; doi:10.1371/journal.pone.0310014)

**Original strips**

Actin


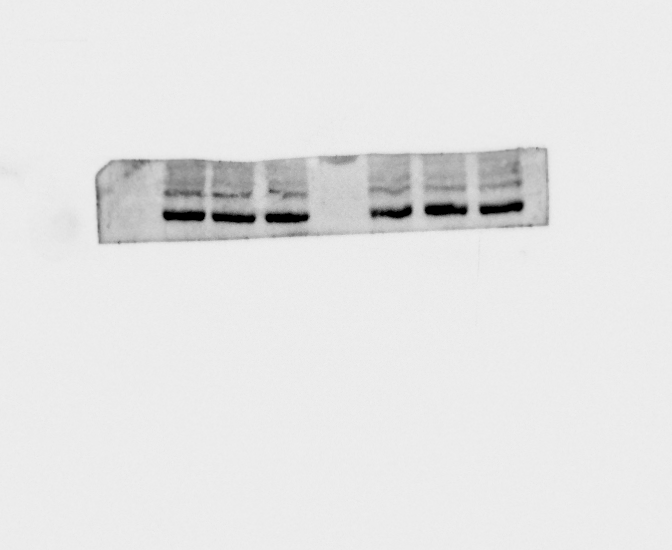

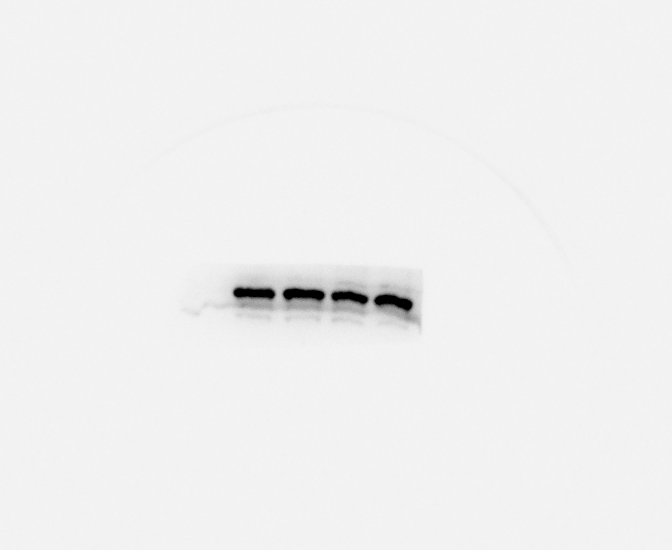


Casp8


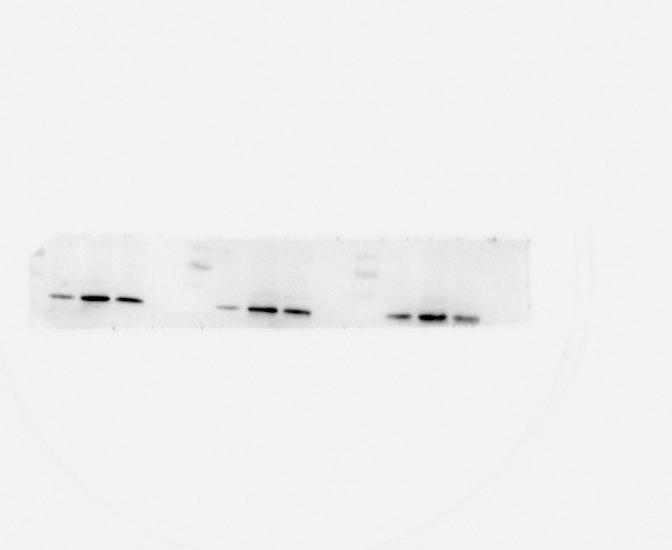


IL-3


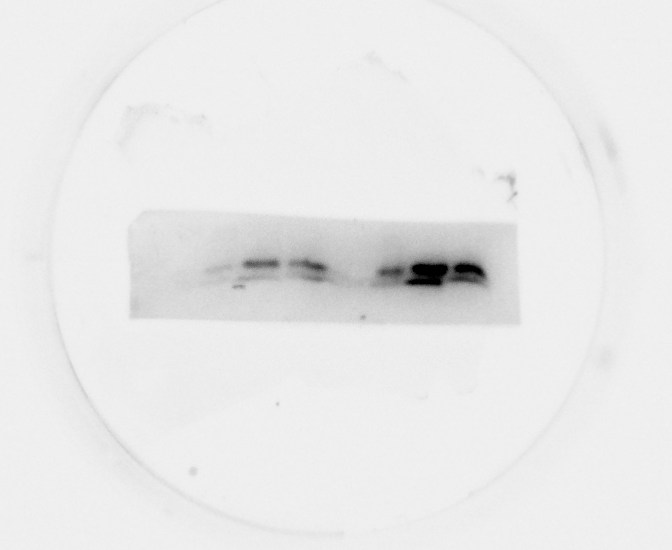

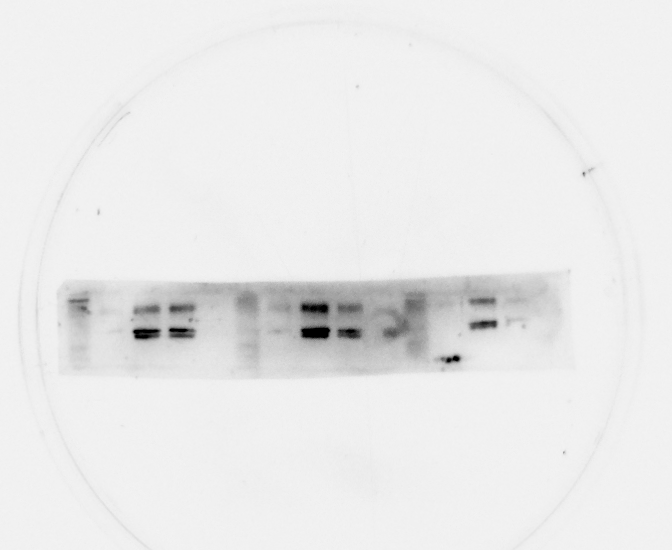


P38


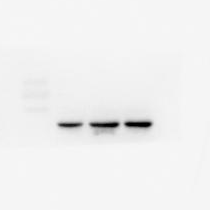

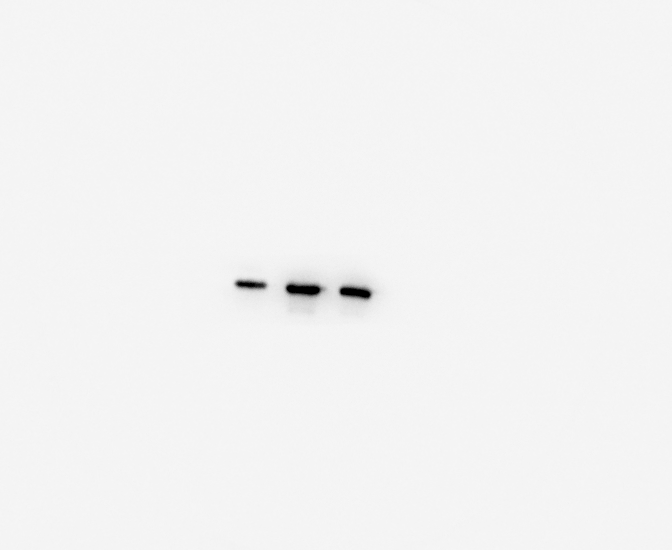


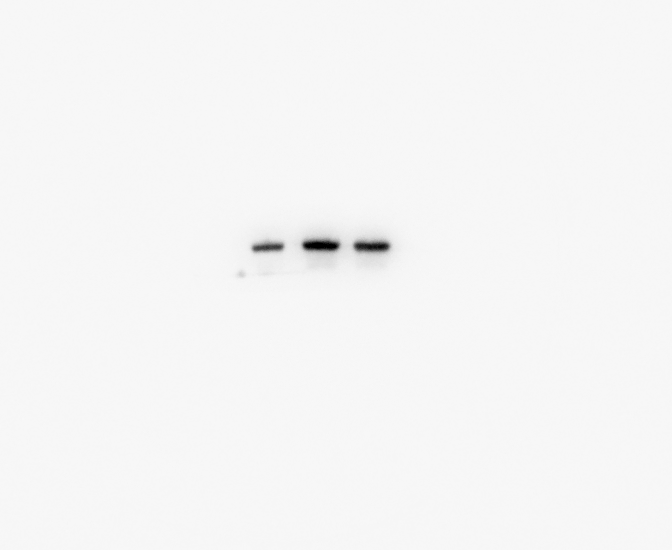


1. p-38


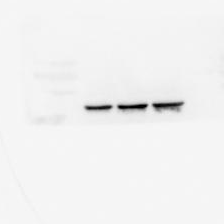

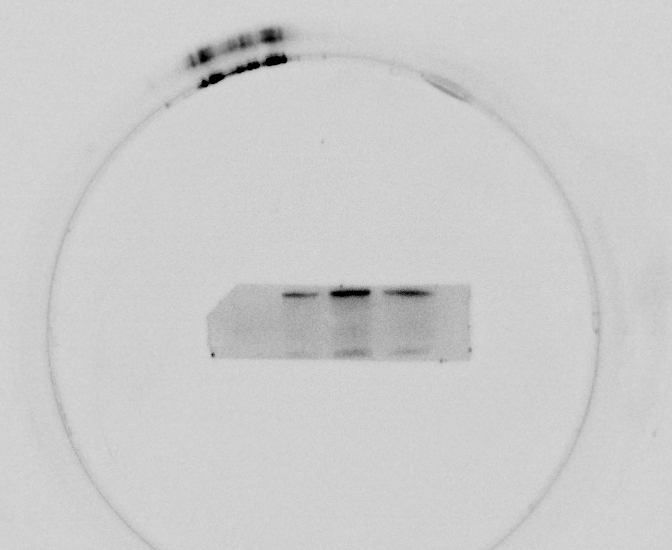

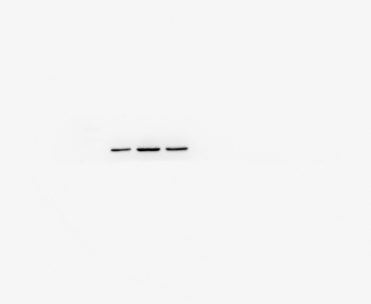


TNF-a


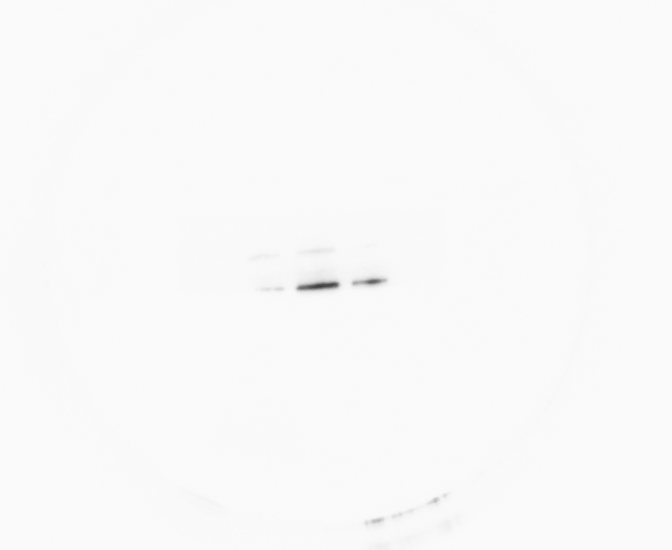

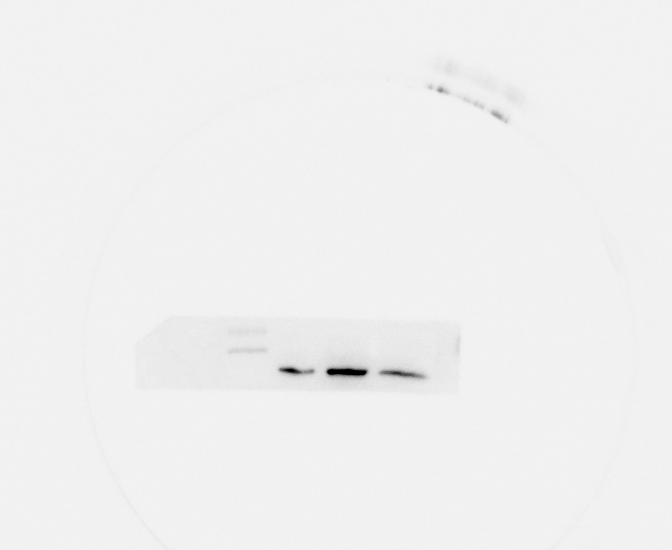

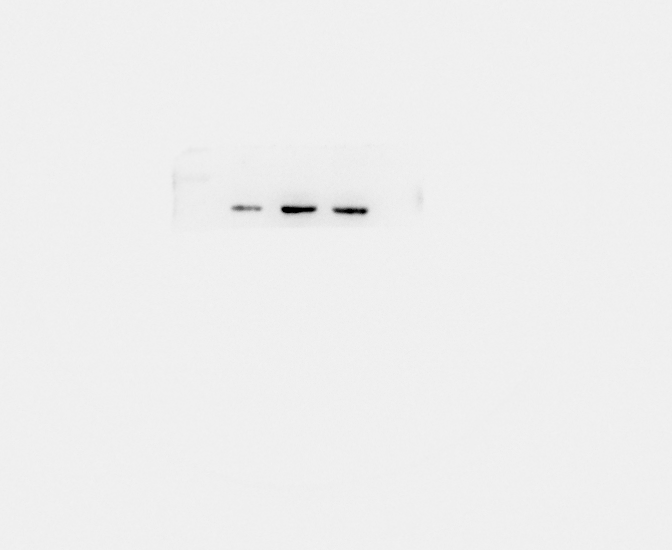


**Processing strips**

**Group 1**

Actin casp8


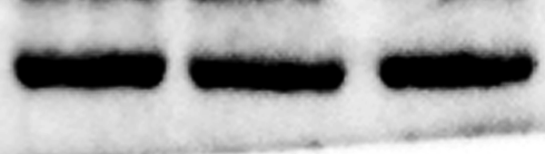

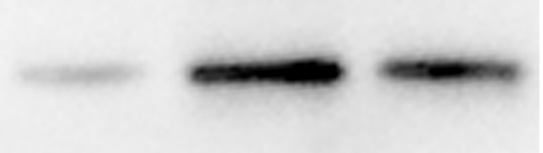


IL-3 p-38


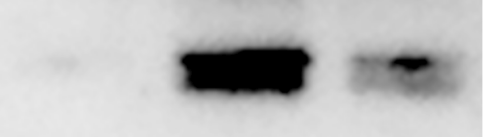

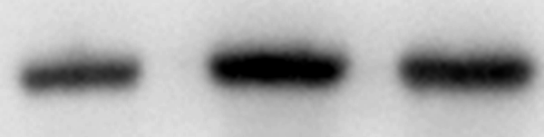


P-p-38 TNF-a


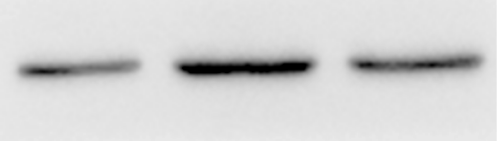

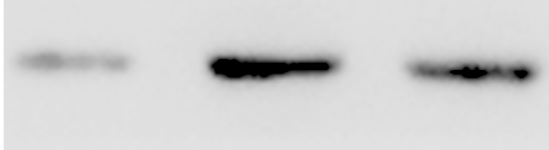


**Group 2**

Actin casp8


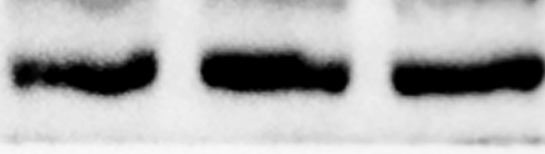

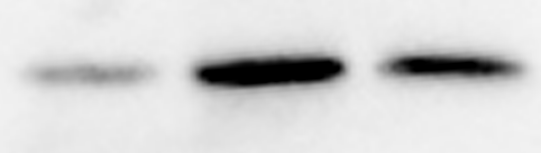


IL-3 p-38


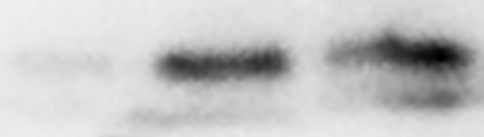

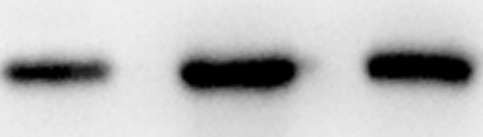


P-p-38 TNF-a


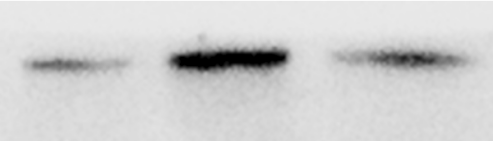

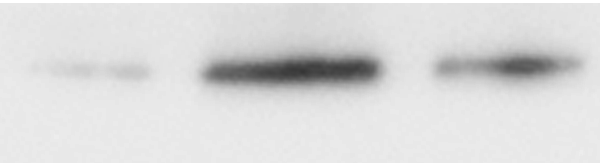


**Group 3**

Actin casp8


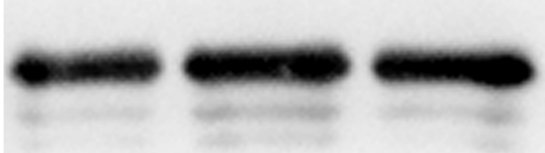

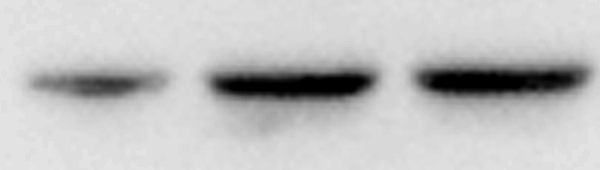


IL-3 p-38


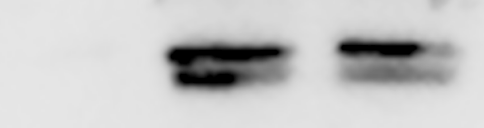

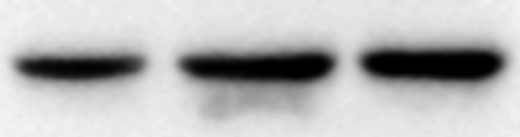


P-p-38 TNF-a


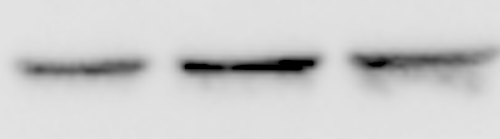

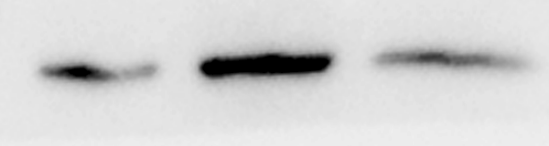

Supplement: S1 File — Additional Supplementary Fig: Fig 1: The Total Ion Current (TIC) overlay plot reveals. Fig 2: The aggregation of QC samples in the 2D PCA score plot. Additional Western Blot: Original strips and Processing strips. Additional pathwaymaps.report: Macrogenome-based analysis of differential pathways across groups. (ZIP) [file pone.0310014.s001.zip › 3 supplement.material/Western Blot/WB (Original strips)/western blot.docx]
