# Supplementary material for: Integrated metagenomic and metabonomic mechanisms for the therapeutic effects of Duhuo Jisheng decoction on intervertebral disc degeneration
Source: PLoS One. 2024 Oct 17;19(10):e0310014. doi: 10.1371/journal.pone.0310014 (PMC11486403; doi:10.1371/journal.pone.0310014)

Original strips

Actin

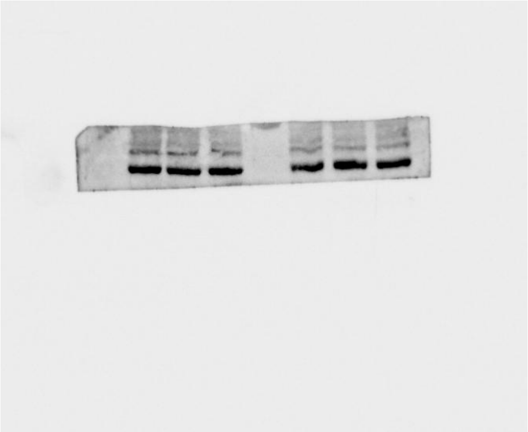 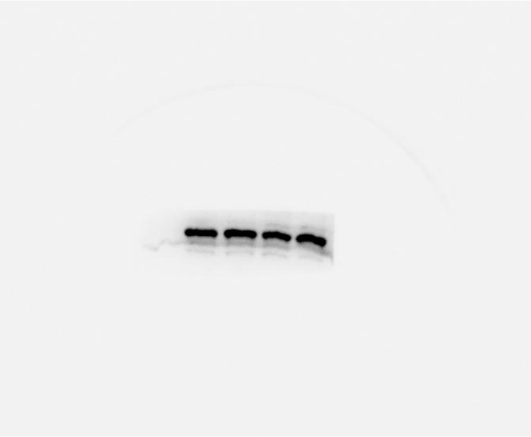

Casp8

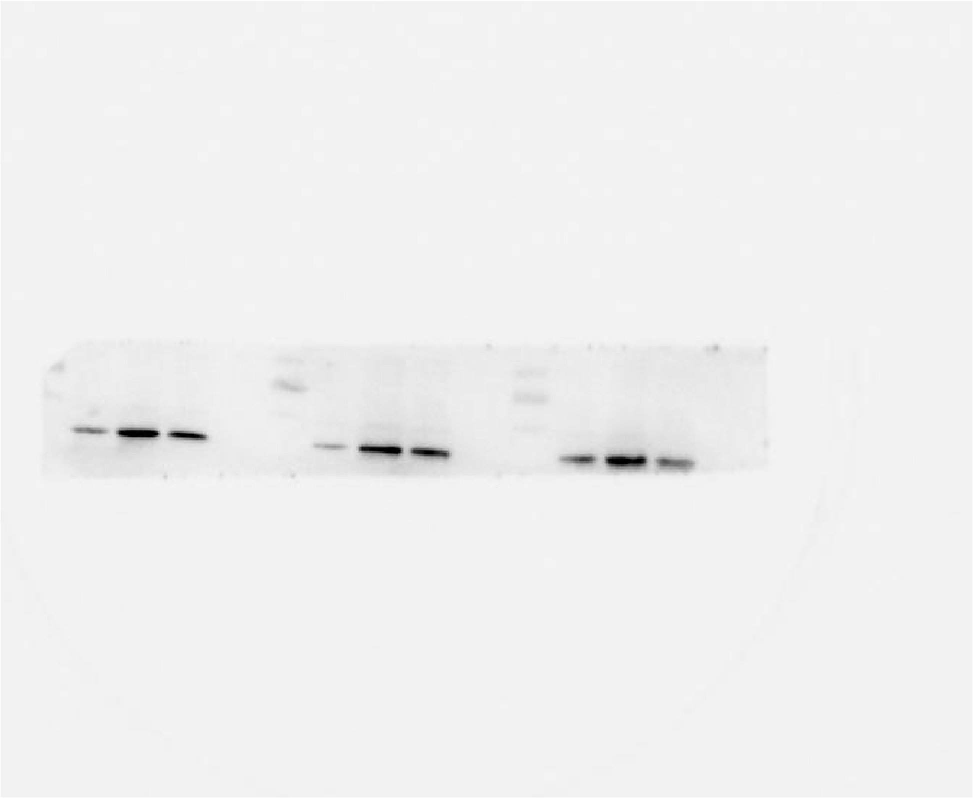

IL-3

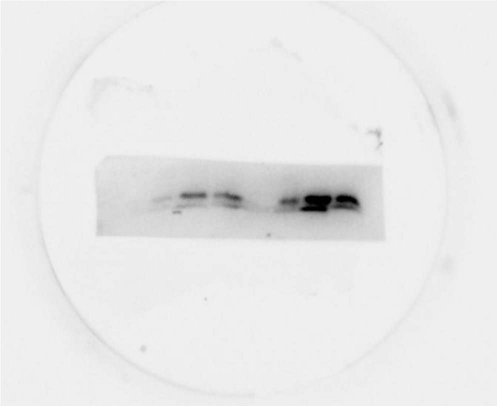 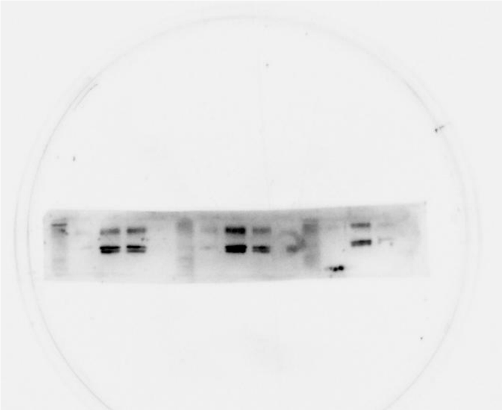

P38

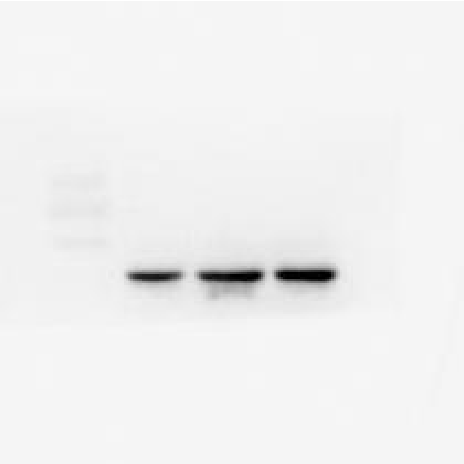 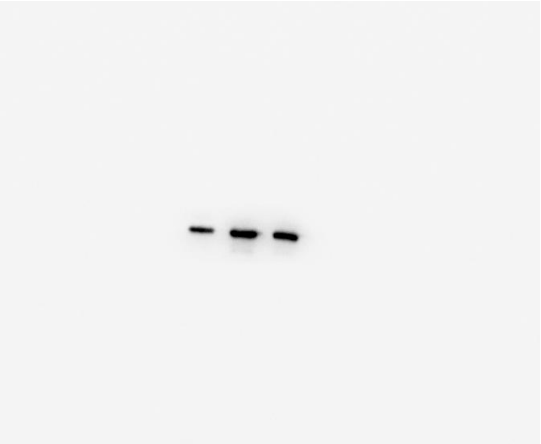

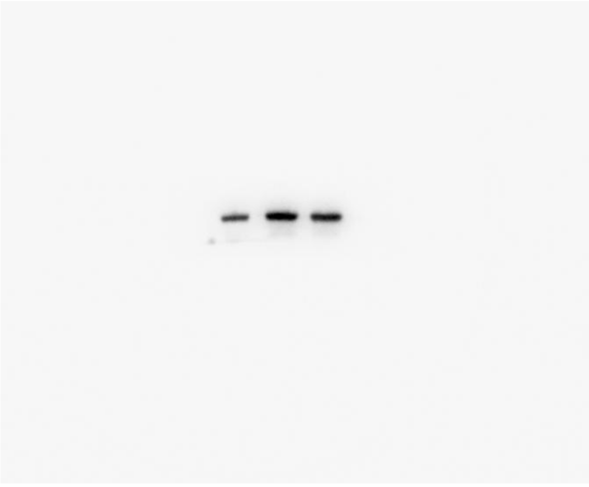

P-p-38

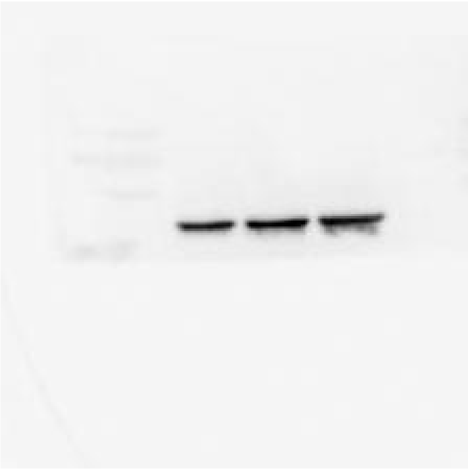

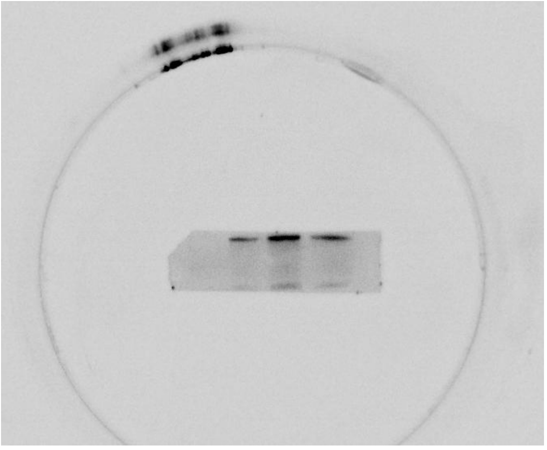

TNF-a

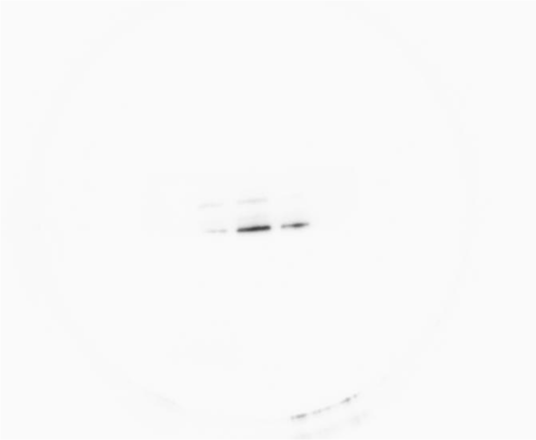

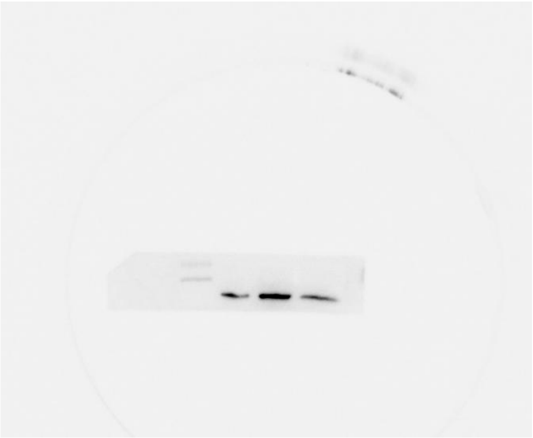

Processing strips

Group 1

Actin

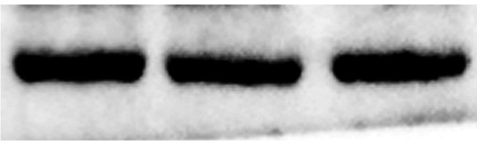

casp8

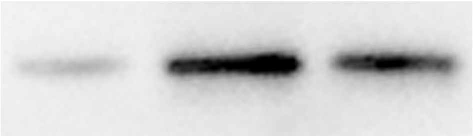

IL-3

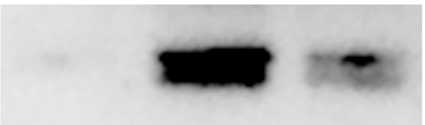

p-38

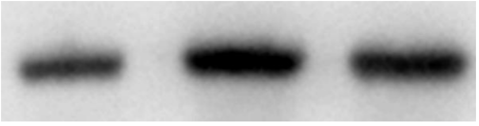

P-p-38

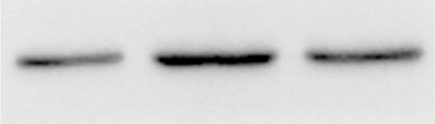

TNF-a

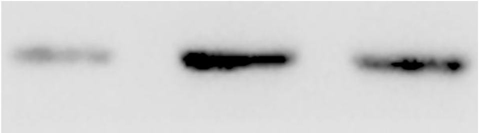

Group 2

Actin

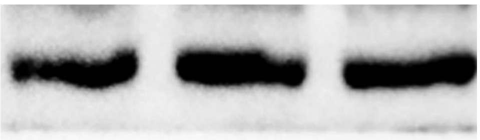

casp8

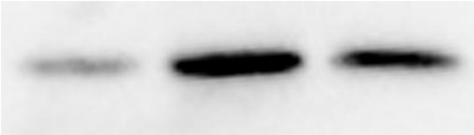

IL-3

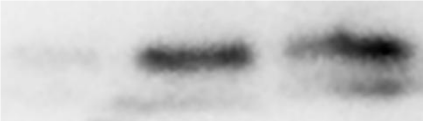

p-38

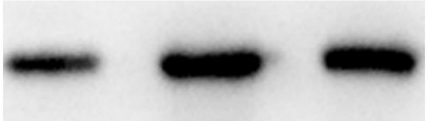

P-p-38

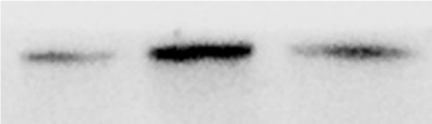

TNF-a

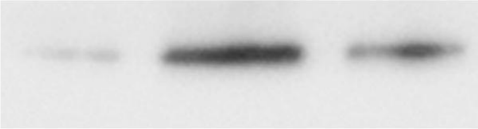

Group 3

Actin

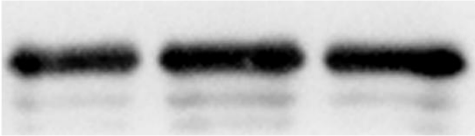

casp8

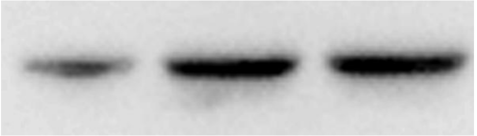

IL-3

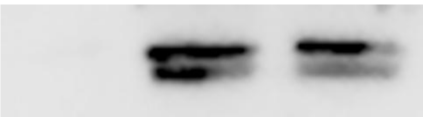

p-38

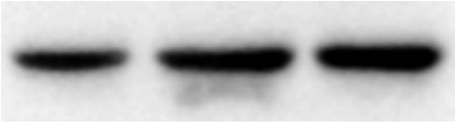

P-p-38

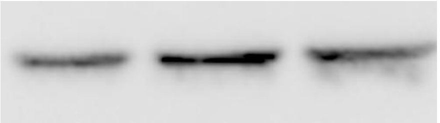

TNF-a

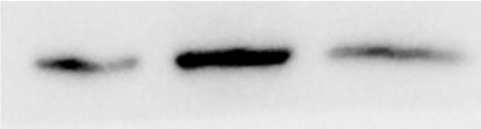

Supplement: S1 File — Additional Supplementary Fig: Fig 1: The Total Ion Current (TIC) overlay plot reveals. Fig 2: The aggregation of QC samples in the 2D PCA score plot. Additional Western Blot: Original strips and Processing strips. Additional pathwaymaps.report: Macrogenome-based analysis of differential pathways across groups. (ZIP) [file pone.0310014.s001.zip › 3 supplement.material/Western Blot/western blot.pdf]
