# Supplementary figures and images for: Integrated metagenomic and metabonomic mechanisms for the therapeutic effects of Duhuo Jisheng decoction on intervertebral disc degeneration
Source: PLoS One. 2024 Oct 17;19(10):e0310014. doi: 10.1371/journal.pone.0310014 (PMC11486403; doi:10.1371/journal.pone.0310014)

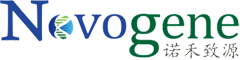

Supplement: S1 File — Additional Supplementary Fig: Fig 1: The Total Ion Current (TIC) overlay plot reveals. Fig 2: The aggregation of QC samples in the 2D PCA score plot. Additional Western Blot: Original strips and Processing strips. Additional pathwaymaps.report: Macrogenome-based analysis of differential pathways across groups. (ZIP) [file pone.0310014.s001.zip › 3 supplement.material/pathwaymaps.report/src/logo.png]

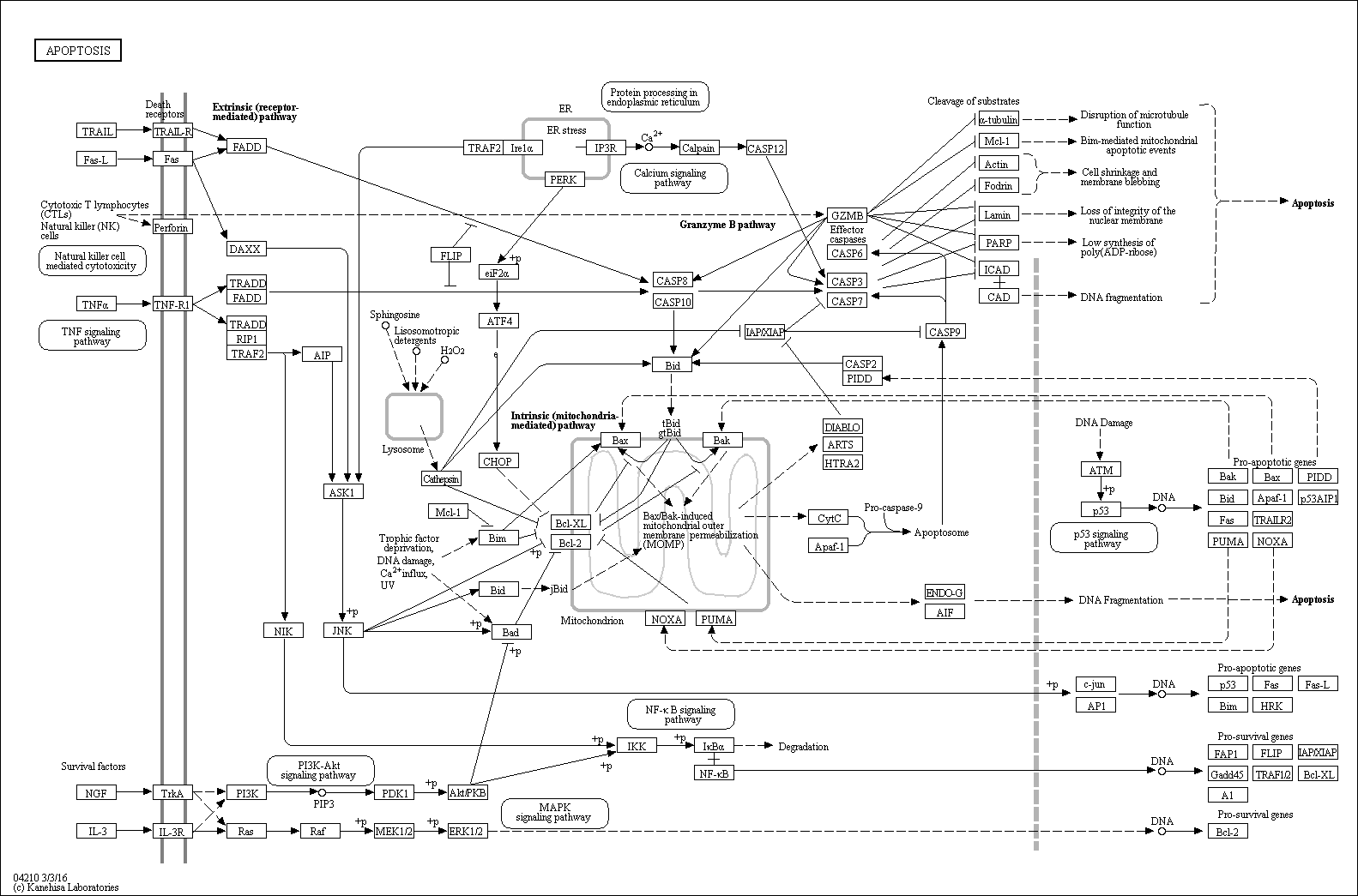

Supplement: S1 File — Additional Supplementary Fig: Fig 1: The Total Ion Current (TIC) overlay plot reveals. Fig 2: The aggregation of QC samples in the 2D PCA score plot. Additional Western Blot: Original strips and Processing strips. Additional pathwaymaps.report: Macrogenome-based analysis of differential pathways across groups. (ZIP) [file pone.0310014.s001.zip › 3 supplement.material/pathwaymaps.report/src/map04210.png]

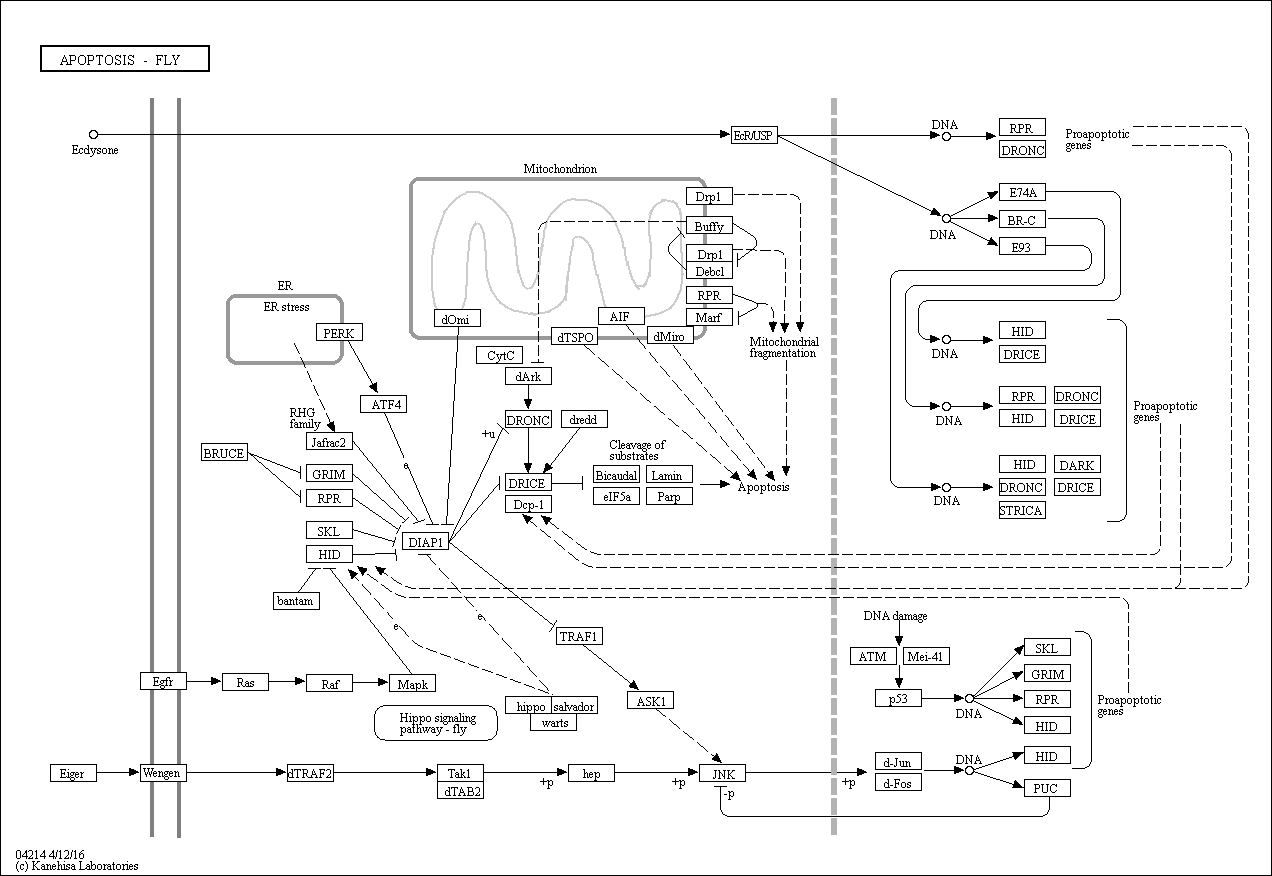

Supplement: S1 File — Additional Supplementary Fig: Fig 1: The Total Ion Current (TIC) overlay plot reveals. Fig 2: The aggregation of QC samples in the 2D PCA score plot. Additional Western Blot: Original strips and Processing strips. Additional pathwaymaps.report: Macrogenome-based analysis of differential pathways across groups. (ZIP) [file pone.0310014.s001.zip › 3 supplement.material/pathwaymaps.report/src/map04214.png]

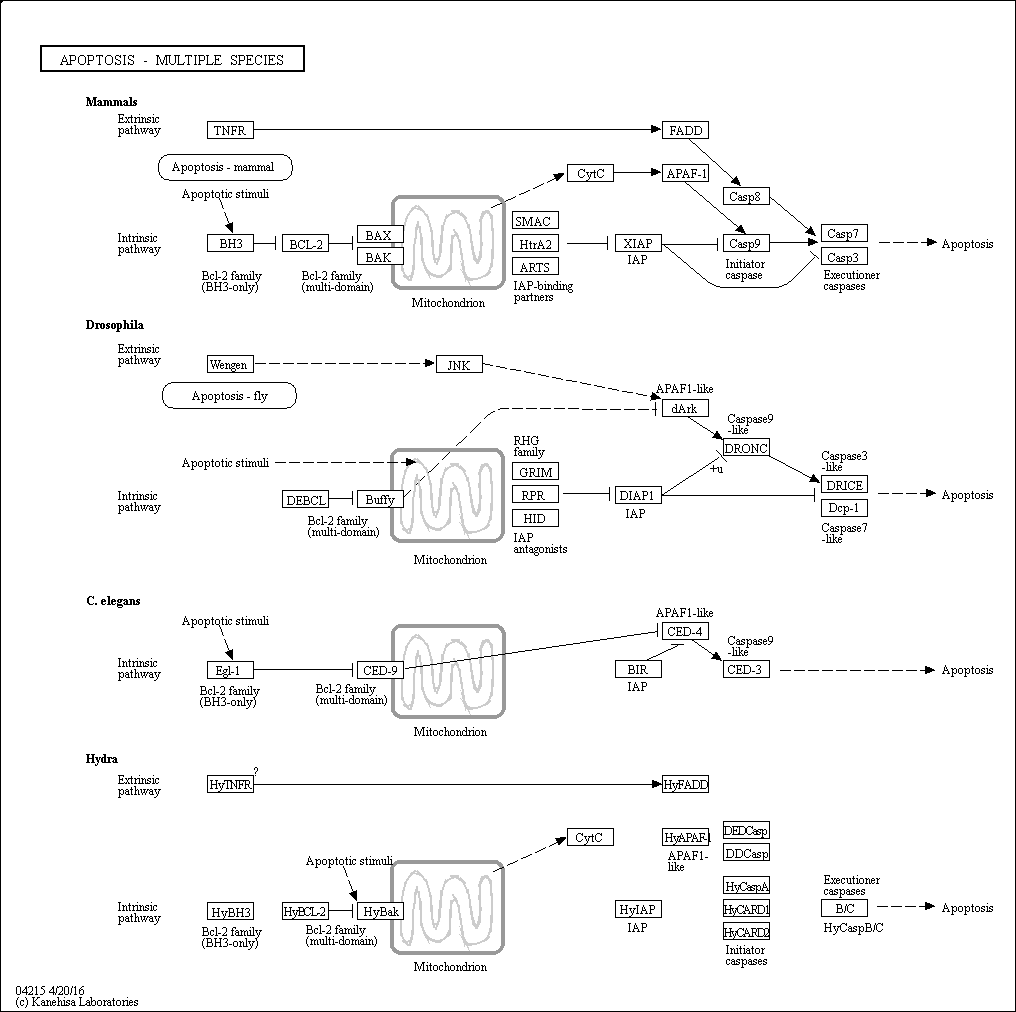

Supplement: S1 File — Additional Supplementary Fig: Fig 1: The Total Ion Current (TIC) overlay plot reveals. Fig 2: The aggregation of QC samples in the 2D PCA score plot. Additional Western Blot: Original strips and Processing strips. Additional pathwaymaps.report: Macrogenome-based analysis of differential pathways across groups. (ZIP) [file pone.0310014.s001.zip › 3 supplement.material/pathwaymaps.report/src/map04215.png]

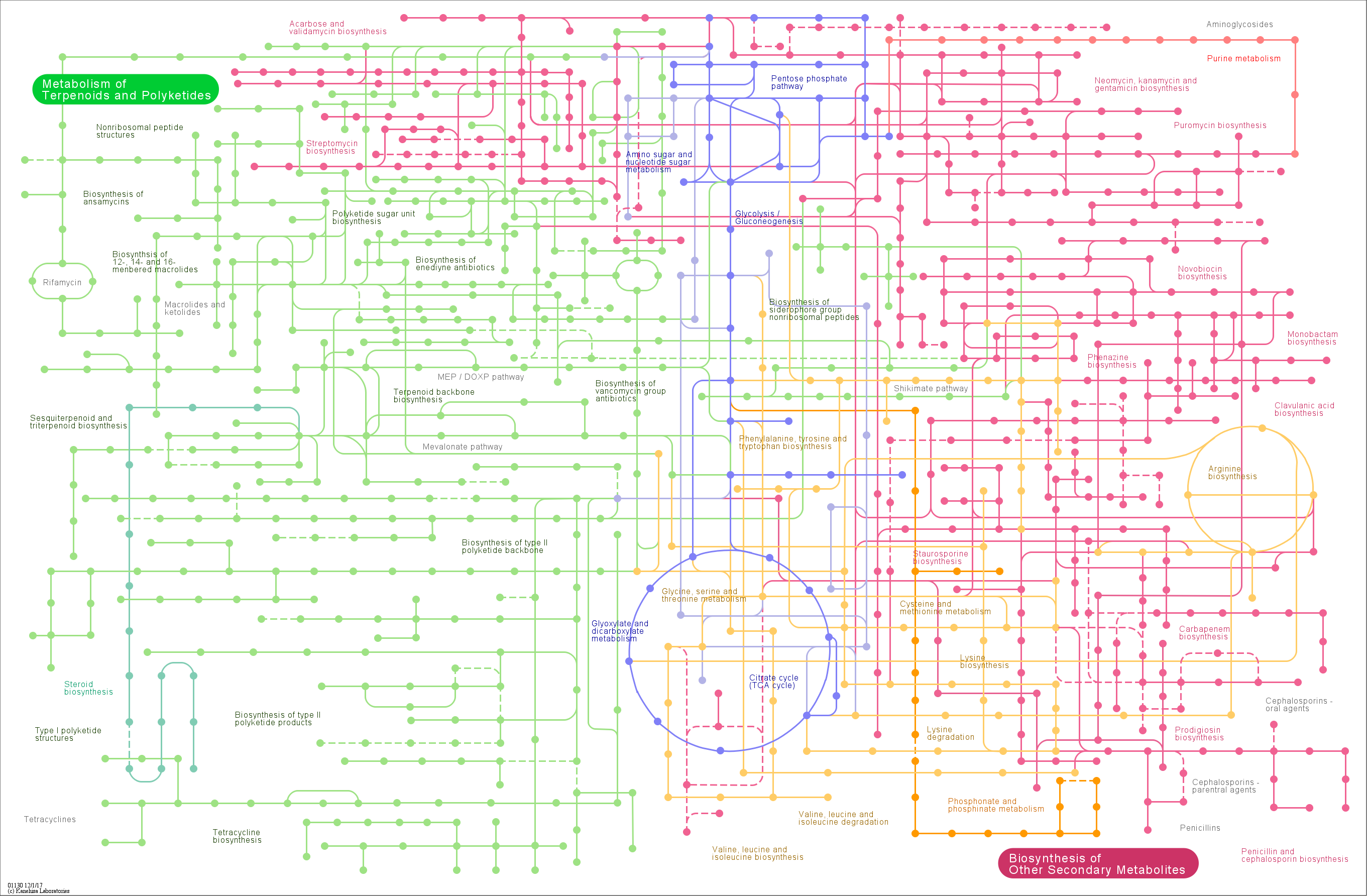

Supplement: S1 File — Additional Supplementary Fig: Fig 1: The Total Ion Current (TIC) overlay plot reveals. Fig 2: The aggregation of QC samples in the 2D PCA score plot. Additional Western Blot: Original strips and Processing strips. Additional pathwaymaps.report: Macrogenome-based analysis of differential pathways across groups. (ZIP) [file pone.0310014.s001.zip › 3 supplement.material/pathwaymaps.report/src/overview/map01130.png]

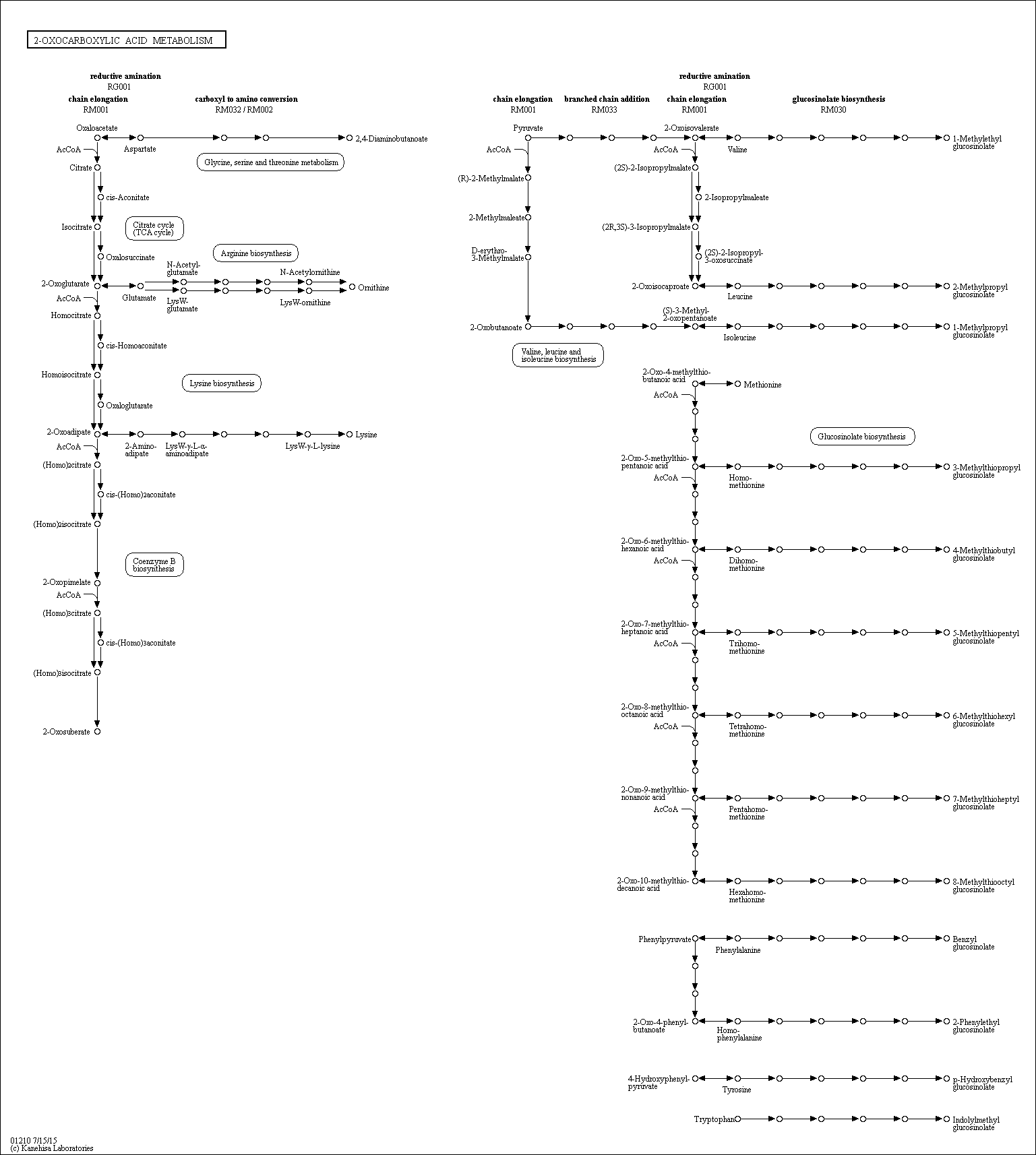

Supplement: S1 File — Additional Supplementary Fig: Fig 1: The Total Ion Current (TIC) overlay plot reveals. Fig 2: The aggregation of QC samples in the 2D PCA score plot. Additional Western Blot: Original strips and Processing strips. Additional pathwaymaps.report: Macrogenome-based analysis of differential pathways across groups. (ZIP) [file pone.0310014.s001.zip › 3 supplement.material/pathwaymaps.report/src/overview/map01210.png]

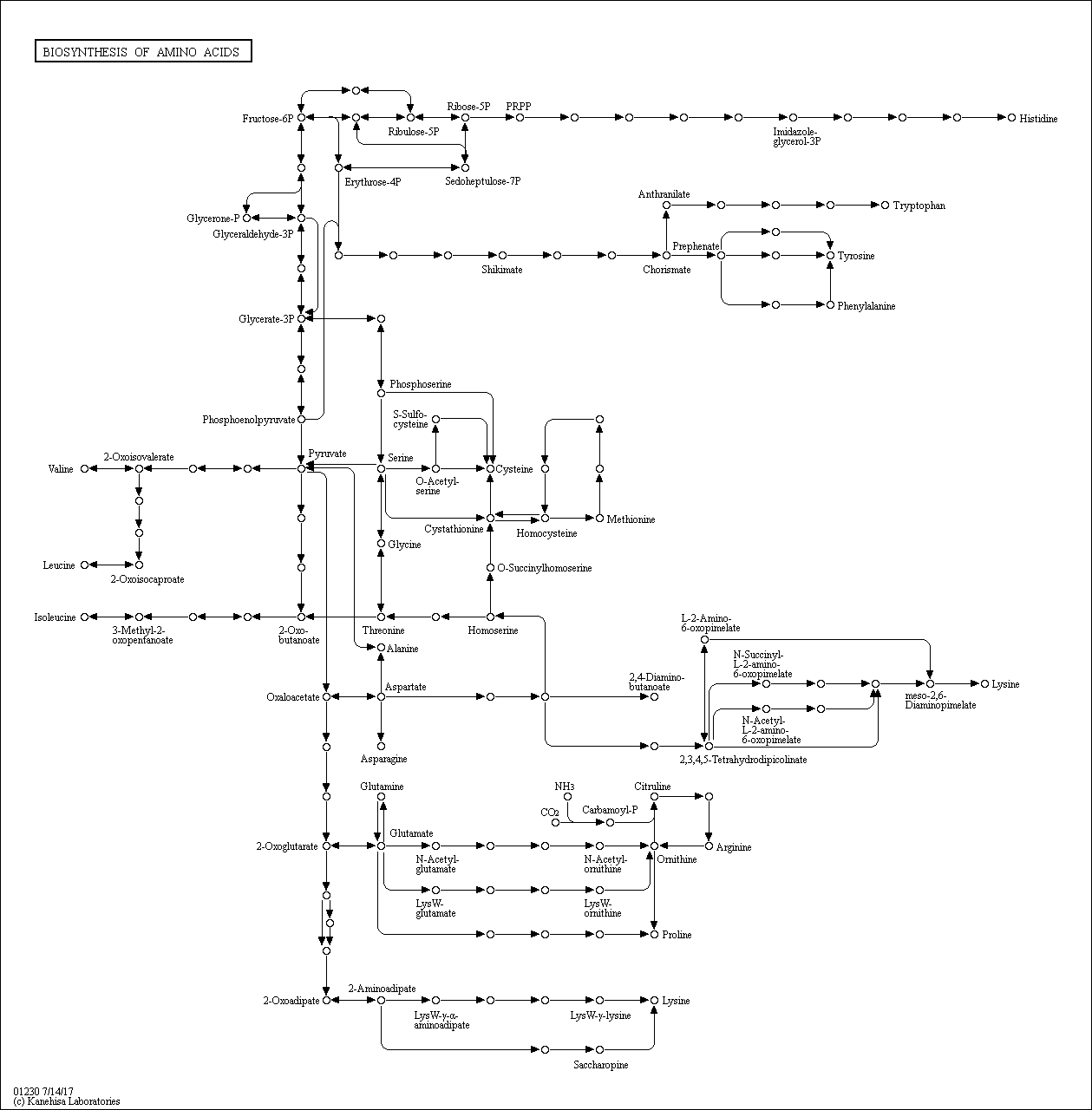

Supplement: S1 File — Additional Supplementary Fig: Fig 1: The Total Ion Current (TIC) overlay plot reveals. Fig 2: The aggregation of QC samples in the 2D PCA score plot. Additional Western Blot: Original strips and Processing strips. Additional pathwaymaps.report: Macrogenome-based analysis of differential pathways across groups. (ZIP) [file pone.0310014.s001.zip › 3 supplement.material/pathwaymaps.report/src/overview/map01230.png]

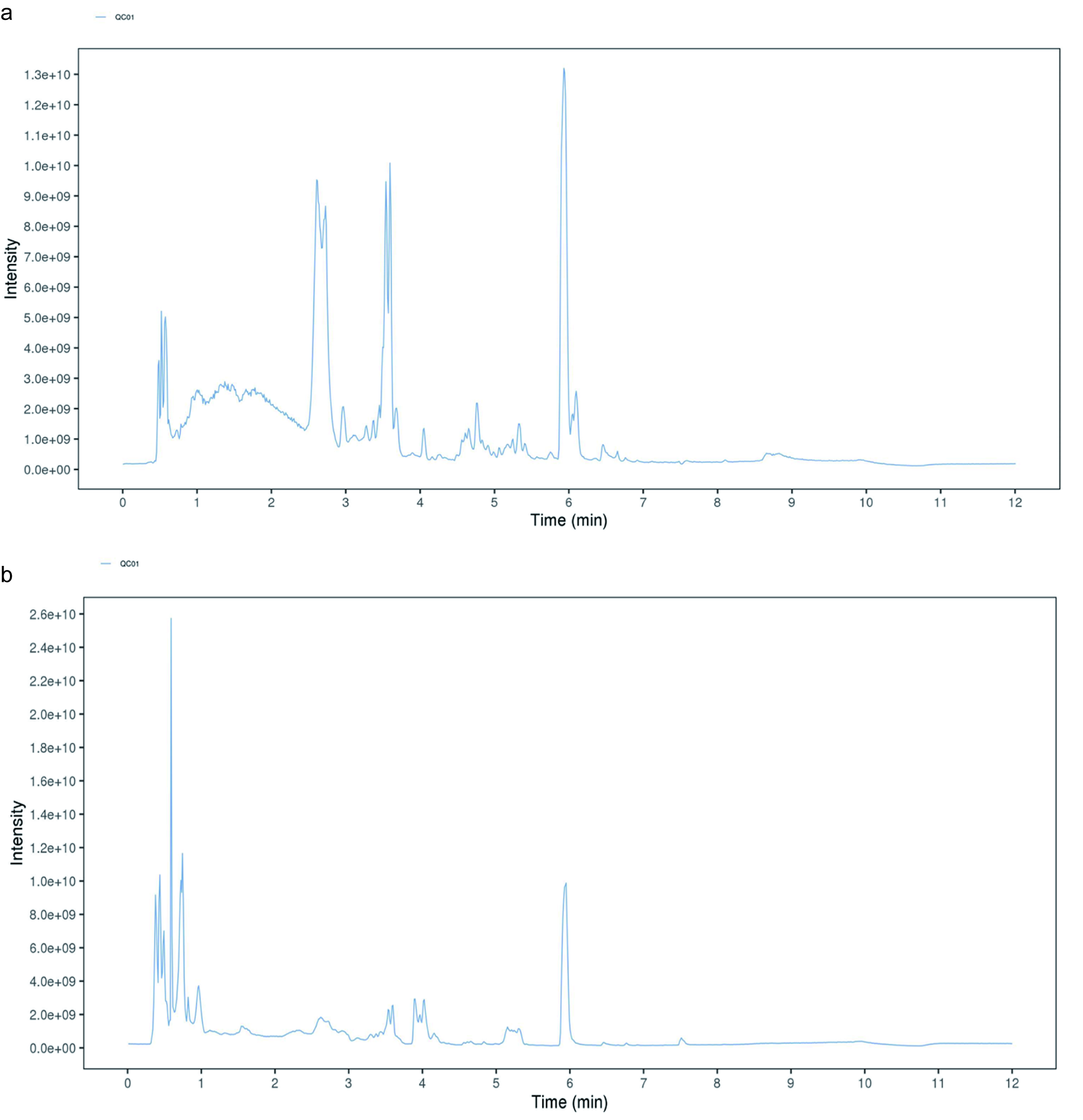

Supplement: S1 File — Additional Supplementary Fig: Fig 1: The Total Ion Current (TIC) overlay plot reveals. Fig 2: The aggregation of QC samples in the 2D PCA score plot. Additional Western Blot: Original strips and Processing strips. Additional pathwaymaps.report: Macrogenome-based analysis of differential pathways across groups. (ZIP) [file pone.0310014.s001.zip › 3 supplement.material/Supplementary Fig/Supplementary Fig 1.tif]

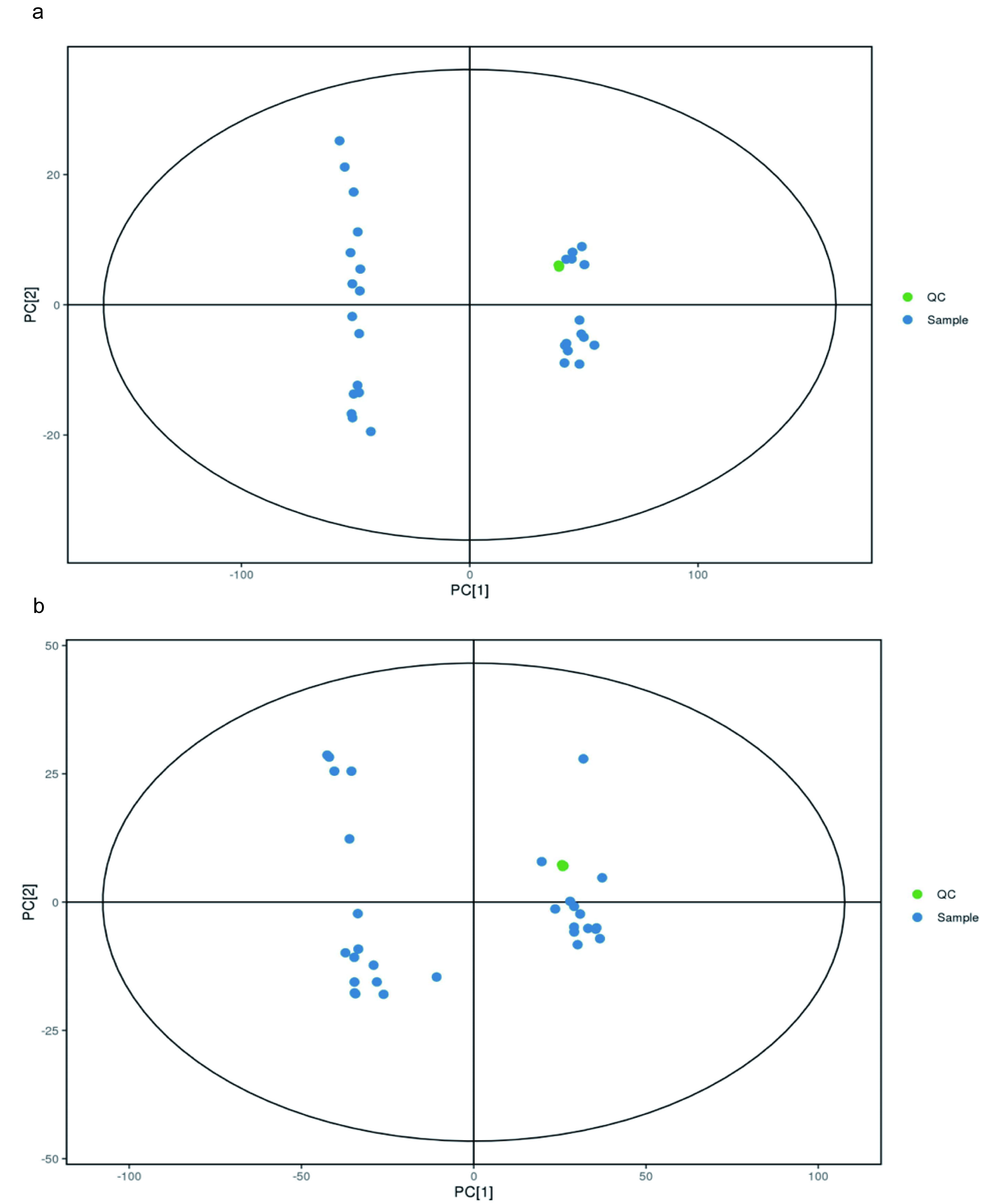

Supplement: S1 File — Additional Supplementary Fig: Fig 1: The Total Ion Current (TIC) overlay plot reveals. Fig 2: The aggregation of QC samples in the 2D PCA score plot. Additional Western Blot: Original strips and Processing strips. Additional pathwaymaps.report: Macrogenome-based analysis of differential pathways across groups. (ZIP) [file pone.0310014.s001.zip › 3 supplement.material/Supplementary Fig/Supplementary Fig 2.tif]

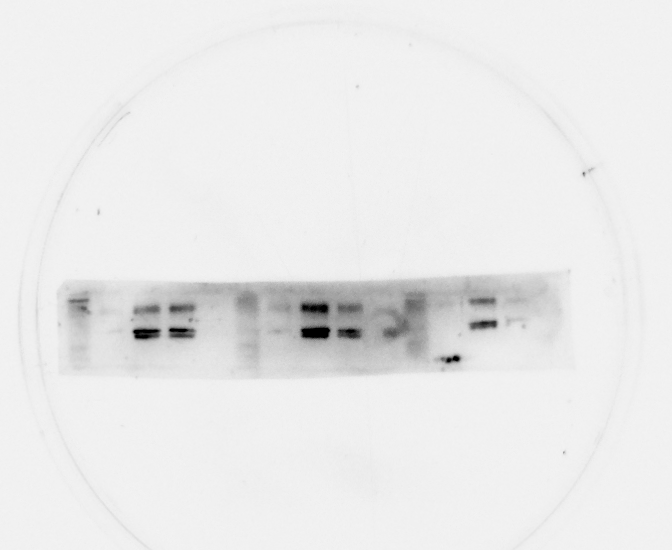

Supplement: S1 File — Additional Supplementary Fig: Fig 1: The Total Ion Current (TIC) overlay plot reveals. Fig 2: The aggregation of QC samples in the 2D PCA score plot. Additional Western Blot: Original strips and Processing strips. Additional pathwaymaps.report: Macrogenome-based analysis of differential pathways across groups. (ZIP) [file pone.0310014.s001.zip › 3 supplement.material/Western Blot/WB (Original strips)/9_已用.tif]

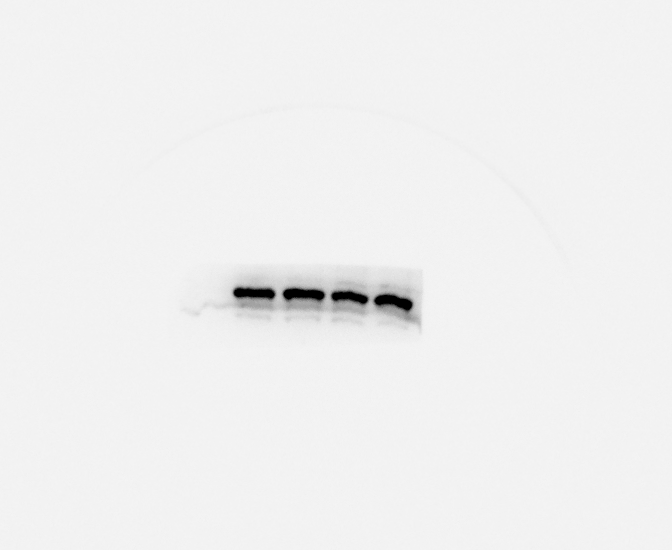

Supplement: S1 File — Additional Supplementary Fig: Fig 1: The Total Ion Current (TIC) overlay plot reveals. Fig 2: The aggregation of QC samples in the 2D PCA score plot. Additional Western Blot: Original strips and Processing strips. Additional pathwaymaps.report: Macrogenome-based analysis of differential pathways across groups. (ZIP) [file pone.0310014.s001.zip › 3 supplement.material/Western Blot/WB (Original strips)/actin-3.tif]

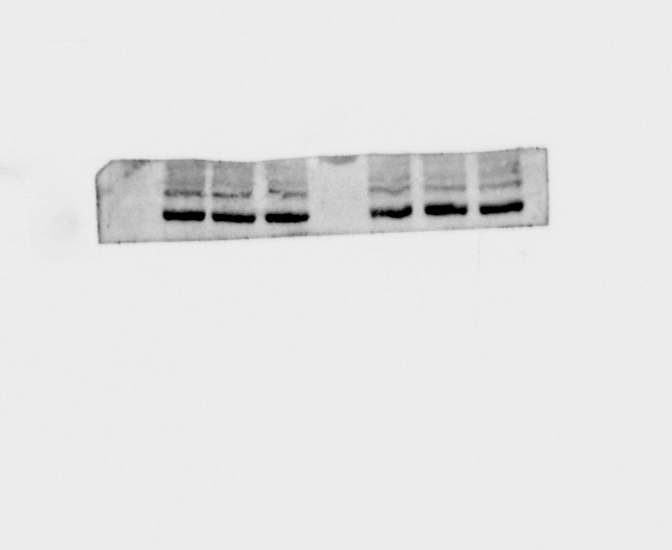

Supplement: S1 File — Additional Supplementary Fig: Fig 1: The Total Ion Current (TIC) overlay plot reveals. Fig 2: The aggregation of QC samples in the 2D PCA score plot. Additional Western Blot: Original strips and Processing strips. Additional pathwaymaps.report: Macrogenome-based analysis of differential pathways across groups. (ZIP) [file pone.0310014.s001.zip › 3 supplement.material/Western Blot/WB (Original strips)/actin1,2.tif]

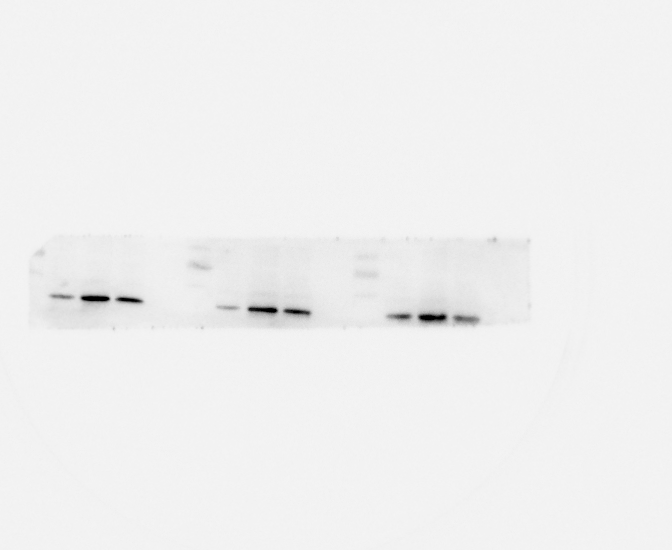

Supplement: S1 File — Additional Supplementary Fig: Fig 1: The Total Ion Current (TIC) overlay plot reveals. Fig 2: The aggregation of QC samples in the 2D PCA score plot. Additional Western Blot: Original strips and Processing strips. Additional pathwaymaps.report: Macrogenome-based analysis of differential pathways across groups. (ZIP) [file pone.0310014.s001.zip › 3 supplement.material/Western Blot/WB (Original strips)/casp8 1,2,3.tif]

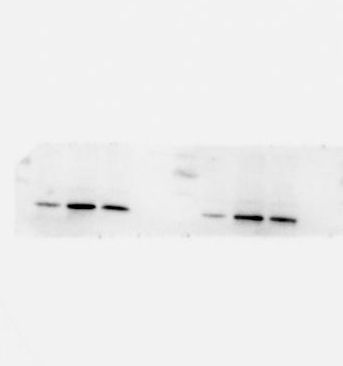

Supplement: S1 File — Additional Supplementary Fig: Fig 1: The Total Ion Current (TIC) overlay plot reveals. Fig 2: The aggregation of QC samples in the 2D PCA score plot. Additional Western Blot: Original strips and Processing strips. Additional pathwaymaps.report: Macrogenome-based analysis of differential pathways across groups. (ZIP) [file pone.0310014.s001.zip › 3 supplement.material/Western Blot/WB (Original strips)/IL-3-2,3.png]

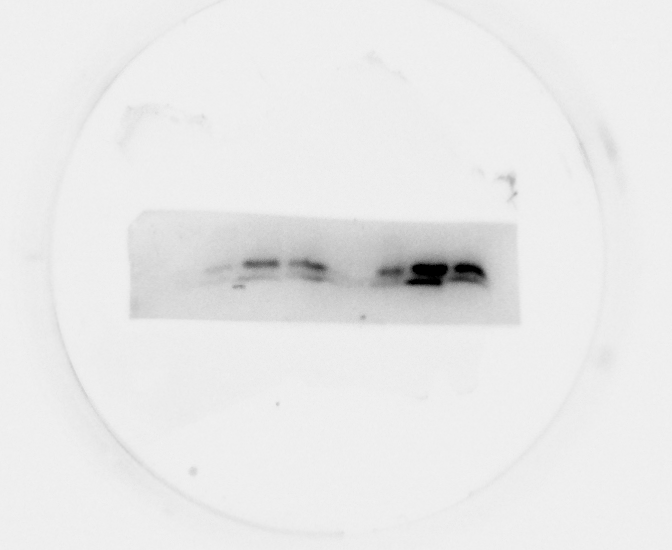

Supplement: S1 File — Additional Supplementary Fig: Fig 1: The Total Ion Current (TIC) overlay plot reveals. Fig 2: The aggregation of QC samples in the 2D PCA score plot. Additional Western Blot: Original strips and Processing strips. Additional pathwaymaps.report: Macrogenome-based analysis of differential pathways across groups. (ZIP) [file pone.0310014.s001.zip › 3 supplement.material/Western Blot/WB (Original strips)/IL_3_1.tif]

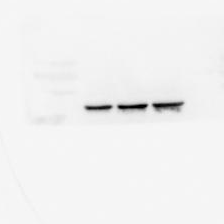

Supplement: S1 File — Additional Supplementary Fig: Fig 1: The Total Ion Current (TIC) overlay plot reveals. Fig 2: The aggregation of QC samples in the 2D PCA score plot. Additional Western Blot: Original strips and Processing strips. Additional pathwaymaps.report: Macrogenome-based analysis of differential pathways across groups. (ZIP) [file pone.0310014.s001.zip › 3 supplement.material/Western Blot/WB (Original strips)/p-p38-1.png]

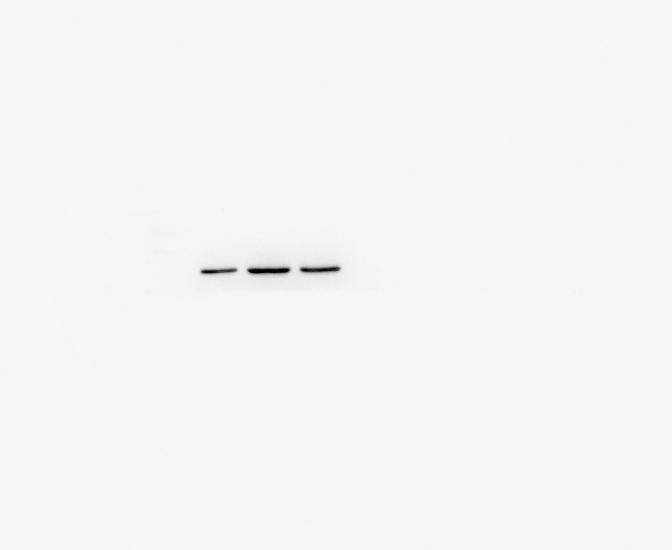

Supplement: S1 File — Additional Supplementary Fig: Fig 1: The Total Ion Current (TIC) overlay plot reveals. Fig 2: The aggregation of QC samples in the 2D PCA score plot. Additional Western Blot: Original strips and Processing strips. Additional pathwaymaps.report: Macrogenome-based analysis of differential pathways across groups. (ZIP) [file pone.0310014.s001.zip › 3 supplement.material/Western Blot/WB (Original strips)/p-p38-3.tif]

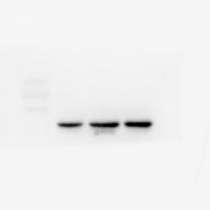

Supplement: S1 File — Additional Supplementary Fig: Fig 1: The Total Ion Current (TIC) overlay plot reveals. Fig 2: The aggregation of QC samples in the 2D PCA score plot. Additional Western Blot: Original strips and Processing strips. Additional pathwaymaps.report: Macrogenome-based analysis of differential pathways across groups. (ZIP) [file pone.0310014.s001.zip › 3 supplement.material/Western Blot/WB (Original strips)/p38-1.png]

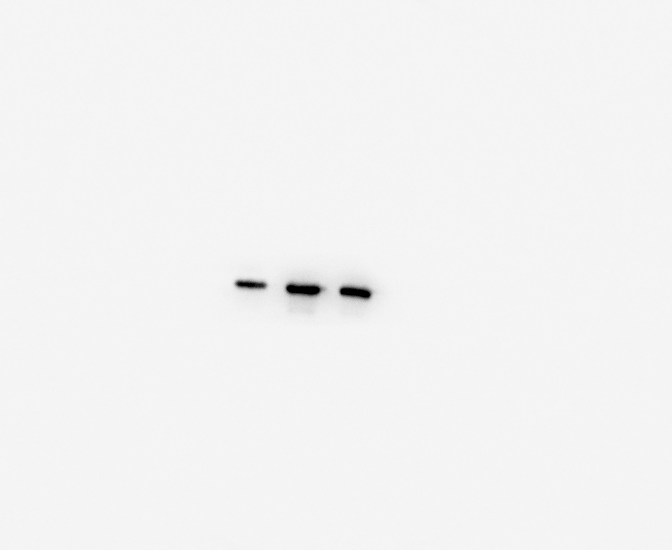

Supplement: S1 File — Additional Supplementary Fig: Fig 1: The Total Ion Current (TIC) overlay plot reveals. Fig 2: The aggregation of QC samples in the 2D PCA score plot. Additional Western Blot: Original strips and Processing strips. Additional pathwaymaps.report: Macrogenome-based analysis of differential pathways across groups. (ZIP) [file pone.0310014.s001.zip › 3 supplement.material/Western Blot/WB (Original strips)/p38-2.tif]

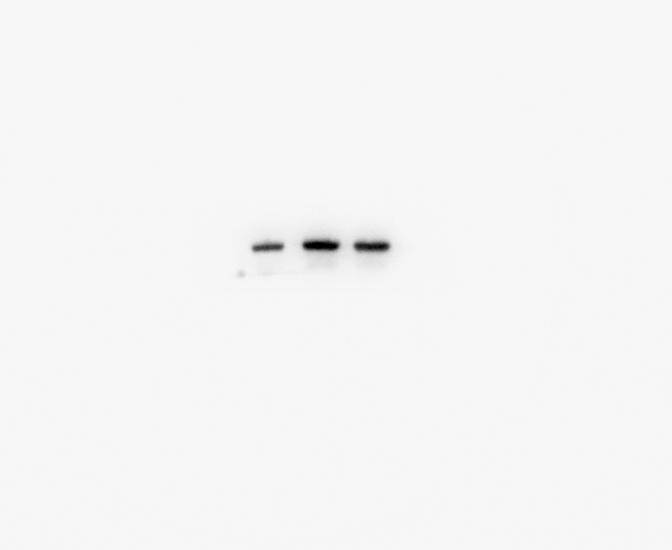

Supplement: S1 File — Additional Supplementary Fig: Fig 1: The Total Ion Current (TIC) overlay plot reveals. Fig 2: The aggregation of QC samples in the 2D PCA score plot. Additional Western Blot: Original strips and Processing strips. Additional pathwaymaps.report: Macrogenome-based analysis of differential pathways across groups. (ZIP) [file pone.0310014.s001.zip › 3 supplement.material/Western Blot/WB (Original strips)/p38-3.tif]

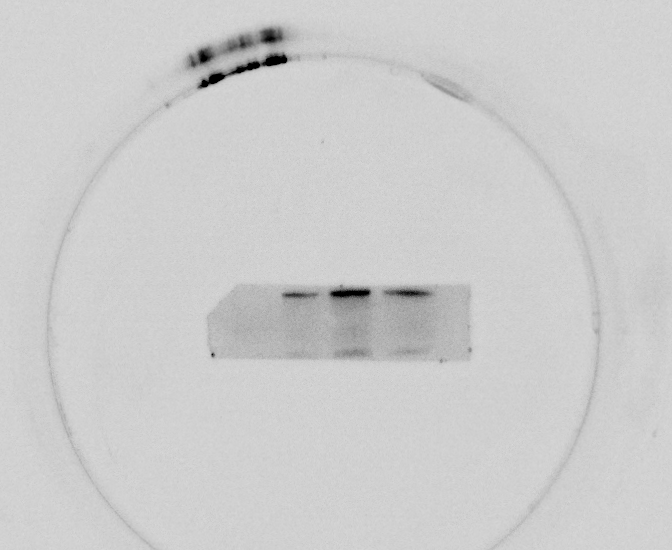

Supplement: S1 File — Additional Supplementary Fig: Fig 1: The Total Ion Current (TIC) overlay plot reveals. Fig 2: The aggregation of QC samples in the 2D PCA score plot. Additional Western Blot: Original strips and Processing strips. Additional pathwaymaps.report: Macrogenome-based analysis of differential pathways across groups. (ZIP) [file pone.0310014.s001.zip › 3 supplement.material/Western Blot/WB (Original strips)/pp38-2.tif]

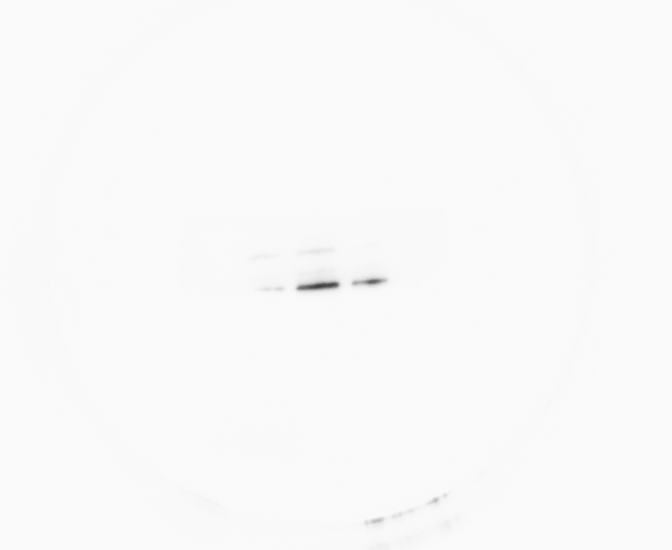

Supplement: S1 File — Additional Supplementary Fig: Fig 1: The Total Ion Current (TIC) overlay plot reveals. Fig 2: The aggregation of QC samples in the 2D PCA score plot. Additional Western Blot: Original strips and Processing strips. Additional pathwaymaps.report: Macrogenome-based analysis of differential pathways across groups. (ZIP) [file pone.0310014.s001.zip › 3 supplement.material/Western Blot/WB (Original strips)/TNF-a-1.tif]

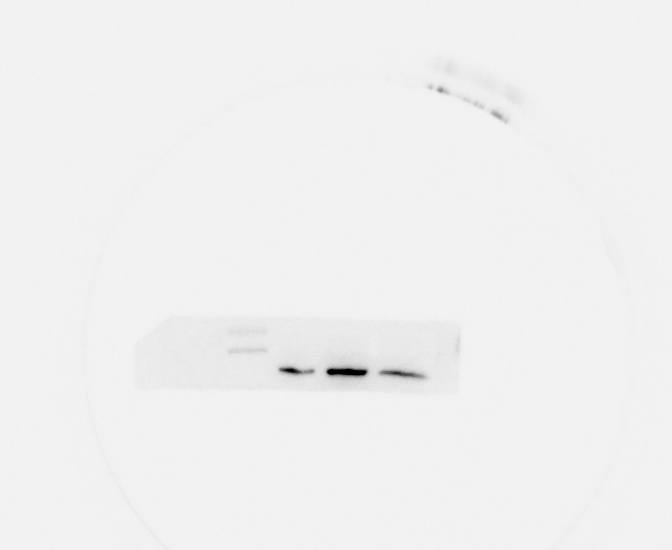

Supplement: S1 File — Additional Supplementary Fig: Fig 1: The Total Ion Current (TIC) overlay plot reveals. Fig 2: The aggregation of QC samples in the 2D PCA score plot. Additional Western Blot: Original strips and Processing strips. Additional pathwaymaps.report: Macrogenome-based analysis of differential pathways across groups. (ZIP) [file pone.0310014.s001.zip › 3 supplement.material/Western Blot/WB (Original strips)/TNF-a-2.tif]

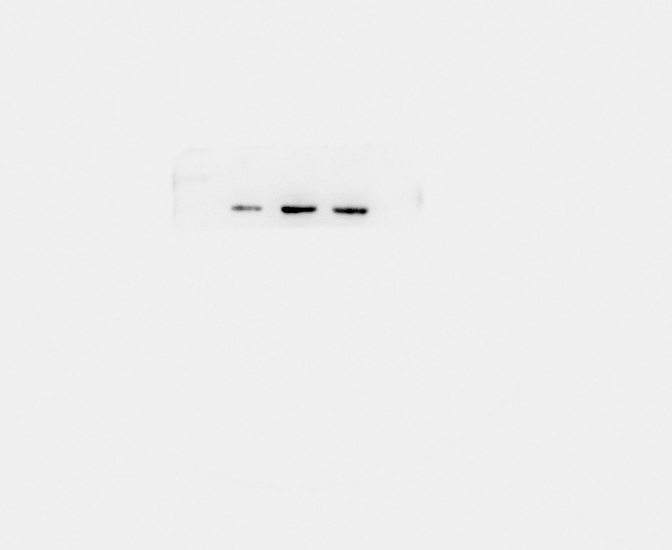

Supplement: S1 File — Additional Supplementary Fig: Fig 1: The Total Ion Current (TIC) overlay plot reveals. Fig 2: The aggregation of QC samples in the 2D PCA score plot. Additional Western Blot: Original strips and Processing strips. Additional pathwaymaps.report: Macrogenome-based analysis of differential pathways across groups. (ZIP) [file pone.0310014.s001.zip › 3 supplement.material/Western Blot/WB (Original strips)/TNF-a-3.tif]

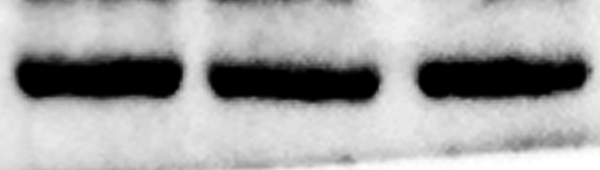

Supplement: S1 File — Additional Supplementary Fig: Fig 1: The Total Ion Current (TIC) overlay plot reveals. Fig 2: The aggregation of QC samples in the 2D PCA score plot. Additional Western Blot: Original strips and Processing strips. Additional pathwaymaps.report: Macrogenome-based analysis of differential pathways across groups. (ZIP) [file pone.0310014.s001.zip › 3 supplement.material/Western Blot/WB(Processing strips)/1-actin.png]

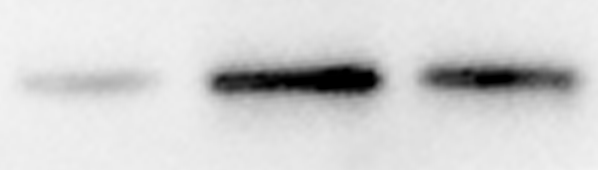

Supplement: S1 File — Additional Supplementary Fig: Fig 1: The Total Ion Current (TIC) overlay plot reveals. Fig 2: The aggregation of QC samples in the 2D PCA score plot. Additional Western Blot: Original strips and Processing strips. Additional pathwaymaps.report: Macrogenome-based analysis of differential pathways across groups. (ZIP) [file pone.0310014.s001.zip › 3 supplement.material/Western Blot/WB(Processing strips)/1-Caspase-8.tif]

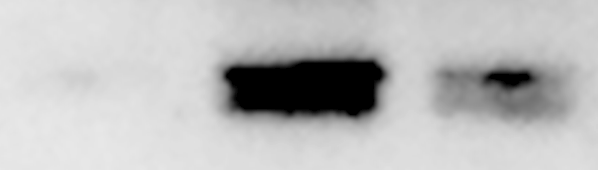

Supplement: S1 File — Additional Supplementary Fig: Fig 1: The Total Ion Current (TIC) overlay plot reveals. Fig 2: The aggregation of QC samples in the 2D PCA score plot. Additional Western Blot: Original strips and Processing strips. Additional pathwaymaps.report: Macrogenome-based analysis of differential pathways across groups. (ZIP) [file pone.0310014.s001.zip › 3 supplement.material/Western Blot/WB(Processing strips)/1-IL-3.tif]

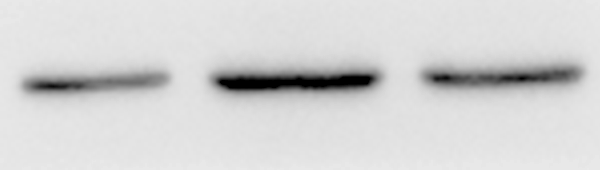

Supplement: S1 File — Additional Supplementary Fig: Fig 1: The Total Ion Current (TIC) overlay plot reveals. Fig 2: The aggregation of QC samples in the 2D PCA score plot. Additional Western Blot: Original strips and Processing strips. Additional pathwaymaps.report: Macrogenome-based analysis of differential pathways across groups. (ZIP) [file pone.0310014.s001.zip › 3 supplement.material/Western Blot/WB(Processing strips)/1-p-p38.png]

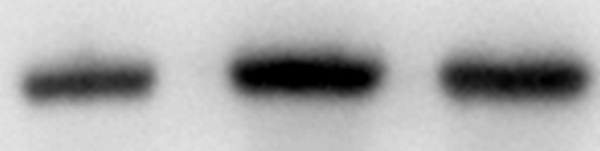

Supplement: S1 File — Additional Supplementary Fig: Fig 1: The Total Ion Current (TIC) overlay plot reveals. Fig 2: The aggregation of QC samples in the 2D PCA score plot. Additional Western Blot: Original strips and Processing strips. Additional pathwaymaps.report: Macrogenome-based analysis of differential pathways across groups. (ZIP) [file pone.0310014.s001.zip › 3 supplement.material/Western Blot/WB(Processing strips)/1-P38.tif]

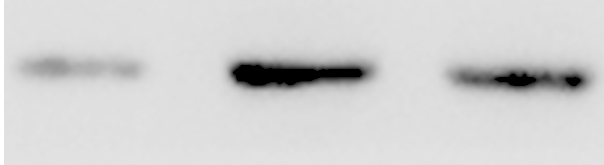

Supplement: S1 File — Additional Supplementary Fig: Fig 1: The Total Ion Current (TIC) overlay plot reveals. Fig 2: The aggregation of QC samples in the 2D PCA score plot. Additional Western Blot: Original strips and Processing strips. Additional pathwaymaps.report: Macrogenome-based analysis of differential pathways across groups. (ZIP) [file pone.0310014.s001.zip › 3 supplement.material/Western Blot/WB(Processing strips)/1-TNF-a.tif]

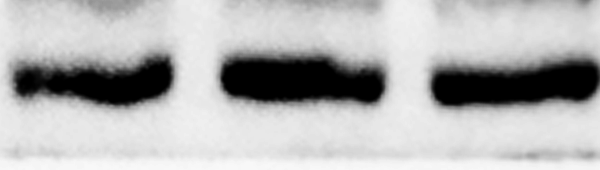

Supplement: S1 File — Additional Supplementary Fig: Fig 1: The Total Ion Current (TIC) overlay plot reveals. Fig 2: The aggregation of QC samples in the 2D PCA score plot. Additional Western Blot: Original strips and Processing strips. Additional pathwaymaps.report: Macrogenome-based analysis of differential pathways across groups. (ZIP) [file pone.0310014.s001.zip › 3 supplement.material/Western Blot/WB(Processing strips)/2-actin.png]

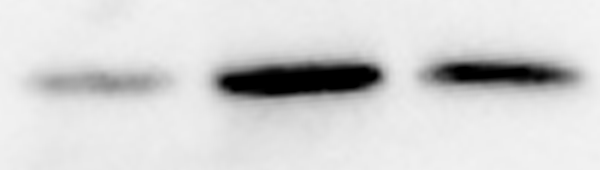

Supplement: S1 File — Additional Supplementary Fig: Fig 1: The Total Ion Current (TIC) overlay plot reveals. Fig 2: The aggregation of QC samples in the 2D PCA score plot. Additional Western Blot: Original strips and Processing strips. Additional pathwaymaps.report: Macrogenome-based analysis of differential pathways across groups. (ZIP) [file pone.0310014.s001.zip › 3 supplement.material/Western Blot/WB(Processing strips)/2-caspase8.png]

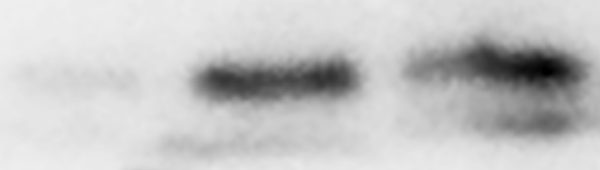

Supplement: S1 File — Additional Supplementary Fig: Fig 1: The Total Ion Current (TIC) overlay plot reveals. Fig 2: The aggregation of QC samples in the 2D PCA score plot. Additional Western Blot: Original strips and Processing strips. Additional pathwaymaps.report: Macrogenome-based analysis of differential pathways across groups. (ZIP) [file pone.0310014.s001.zip › 3 supplement.material/Western Blot/WB(Processing strips)/2-IL-3.png]

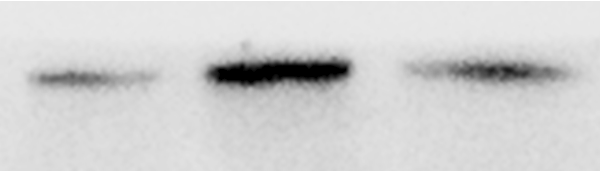

Supplement: S1 File — Additional Supplementary Fig: Fig 1: The Total Ion Current (TIC) overlay plot reveals. Fig 2: The aggregation of QC samples in the 2D PCA score plot. Additional Western Blot: Original strips and Processing strips. Additional pathwaymaps.report: Macrogenome-based analysis of differential pathways across groups. (ZIP) [file pone.0310014.s001.zip › 3 supplement.material/Western Blot/WB(Processing strips)/2-p-p38.png]

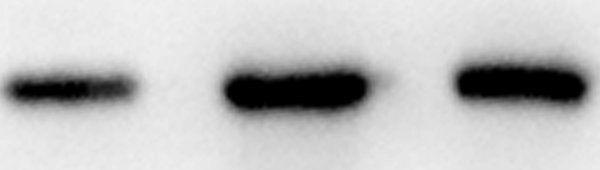

Supplement: S1 File — Additional Supplementary Fig: Fig 1: The Total Ion Current (TIC) overlay plot reveals. Fig 2: The aggregation of QC samples in the 2D PCA score plot. Additional Western Blot: Original strips and Processing strips. Additional pathwaymaps.report: Macrogenome-based analysis of differential pathways across groups. (ZIP) [file pone.0310014.s001.zip › 3 supplement.material/Western Blot/WB(Processing strips)/2-p38.png]

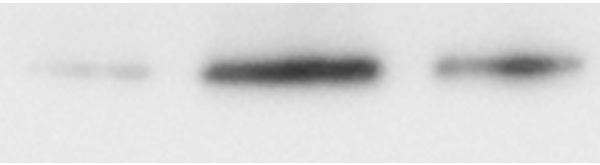

Supplement: S1 File — Additional Supplementary Fig: Fig 1: The Total Ion Current (TIC) overlay plot reveals. Fig 2: The aggregation of QC samples in the 2D PCA score plot. Additional Western Blot: Original strips and Processing strips. Additional pathwaymaps.report: Macrogenome-based analysis of differential pathways across groups. (ZIP) [file pone.0310014.s001.zip › 3 supplement.material/Western Blot/WB(Processing strips)/2-TNF-a.png]

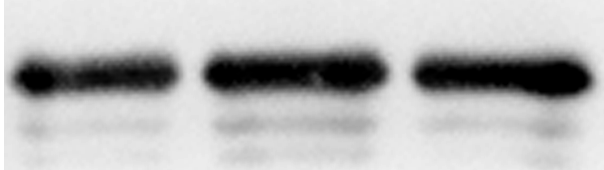

Supplement: S1 File — Additional Supplementary Fig: Fig 1: The Total Ion Current (TIC) overlay plot reveals. Fig 2: The aggregation of QC samples in the 2D PCA score plot. Additional Western Blot: Original strips and Processing strips. Additional pathwaymaps.report: Macrogenome-based analysis of differential pathways across groups. (ZIP) [file pone.0310014.s001.zip › 3 supplement.material/Western Blot/WB(Processing strips)/3-actin.png]

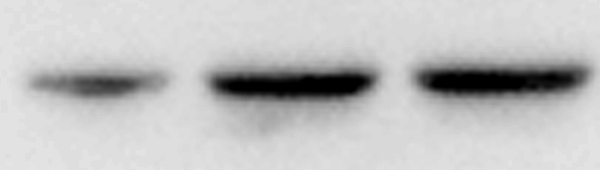

Supplement: S1 File — Additional Supplementary Fig: Fig 1: The Total Ion Current (TIC) overlay plot reveals. Fig 2: The aggregation of QC samples in the 2D PCA score plot. Additional Western Blot: Original strips and Processing strips. Additional pathwaymaps.report: Macrogenome-based analysis of differential pathways across groups. (ZIP) [file pone.0310014.s001.zip › 3 supplement.material/Western Blot/WB(Processing strips)/3-caspase8.png]

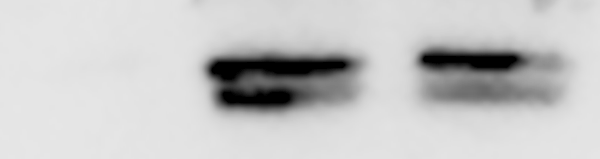

Supplement: S1 File — Additional Supplementary Fig: Fig 1: The Total Ion Current (TIC) overlay plot reveals. Fig 2: The aggregation of QC samples in the 2D PCA score plot. Additional Western Blot: Original strips and Processing strips. Additional pathwaymaps.report: Macrogenome-based analysis of differential pathways across groups. (ZIP) [file pone.0310014.s001.zip › 3 supplement.material/Western Blot/WB(Processing strips)/3-IL-3.png]

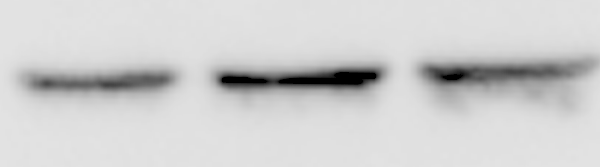

Supplement: S1 File — Additional Supplementary Fig: Fig 1: The Total Ion Current (TIC) overlay plot reveals. Fig 2: The aggregation of QC samples in the 2D PCA score plot. Additional Western Blot: Original strips and Processing strips. Additional pathwaymaps.report: Macrogenome-based analysis of differential pathways across groups. (ZIP) [file pone.0310014.s001.zip › 3 supplement.material/Western Blot/WB(Processing strips)/3-p-p38.png]

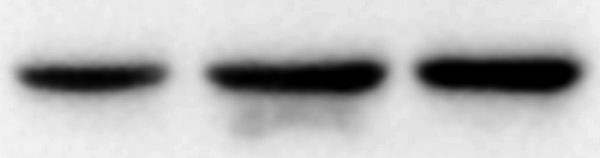

Supplement: S1 File — Additional Supplementary Fig: Fig 1: The Total Ion Current (TIC) overlay plot reveals. Fig 2: The aggregation of QC samples in the 2D PCA score plot. Additional Western Blot: Original strips and Processing strips. Additional pathwaymaps.report: Macrogenome-based analysis of differential pathways across groups. (ZIP) [file pone.0310014.s001.zip › 3 supplement.material/Western Blot/WB(Processing strips)/3-p38.png]

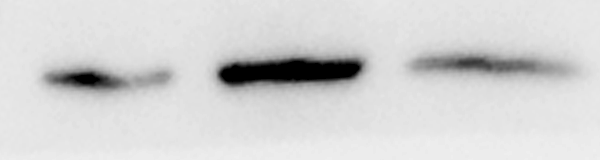

Supplement: S1 File — Additional Supplementary Fig: Fig 1: The Total Ion Current (TIC) overlay plot reveals. Fig 2: The aggregation of QC samples in the 2D PCA score plot. Additional Western Blot: Original strips and Processing strips. Additional pathwaymaps.report: Macrogenome-based analysis of differential pathways across groups. (ZIP) [file pone.0310014.s001.zip › 3 supplement.material/Western Blot/WB(Processing strips)/3-TNF-a.png]

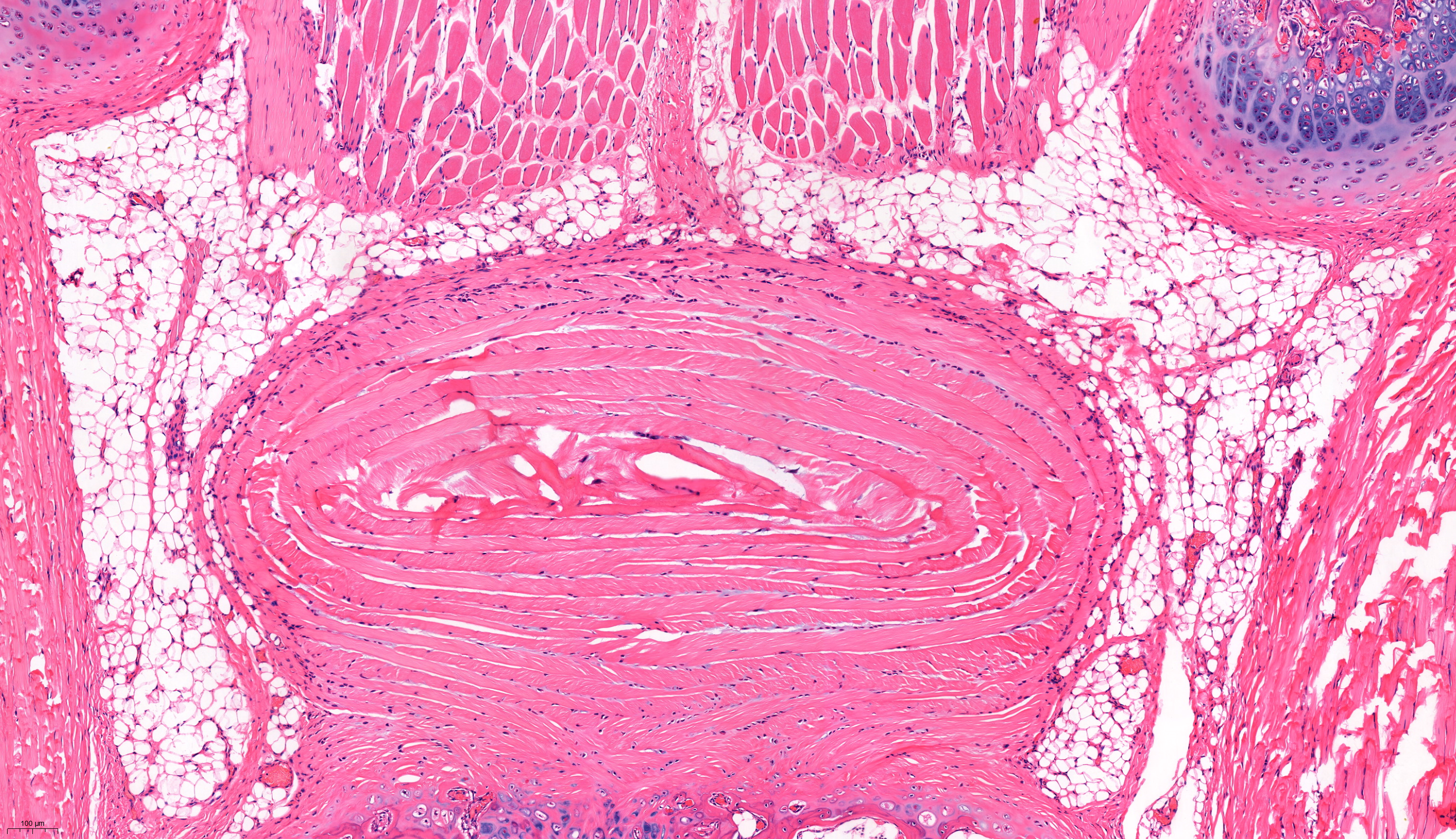

Supplement: S2 File — (ZIP) [file pone.0310014.s002.zip › HE triple section image/Celecoxib1 HE.tif]

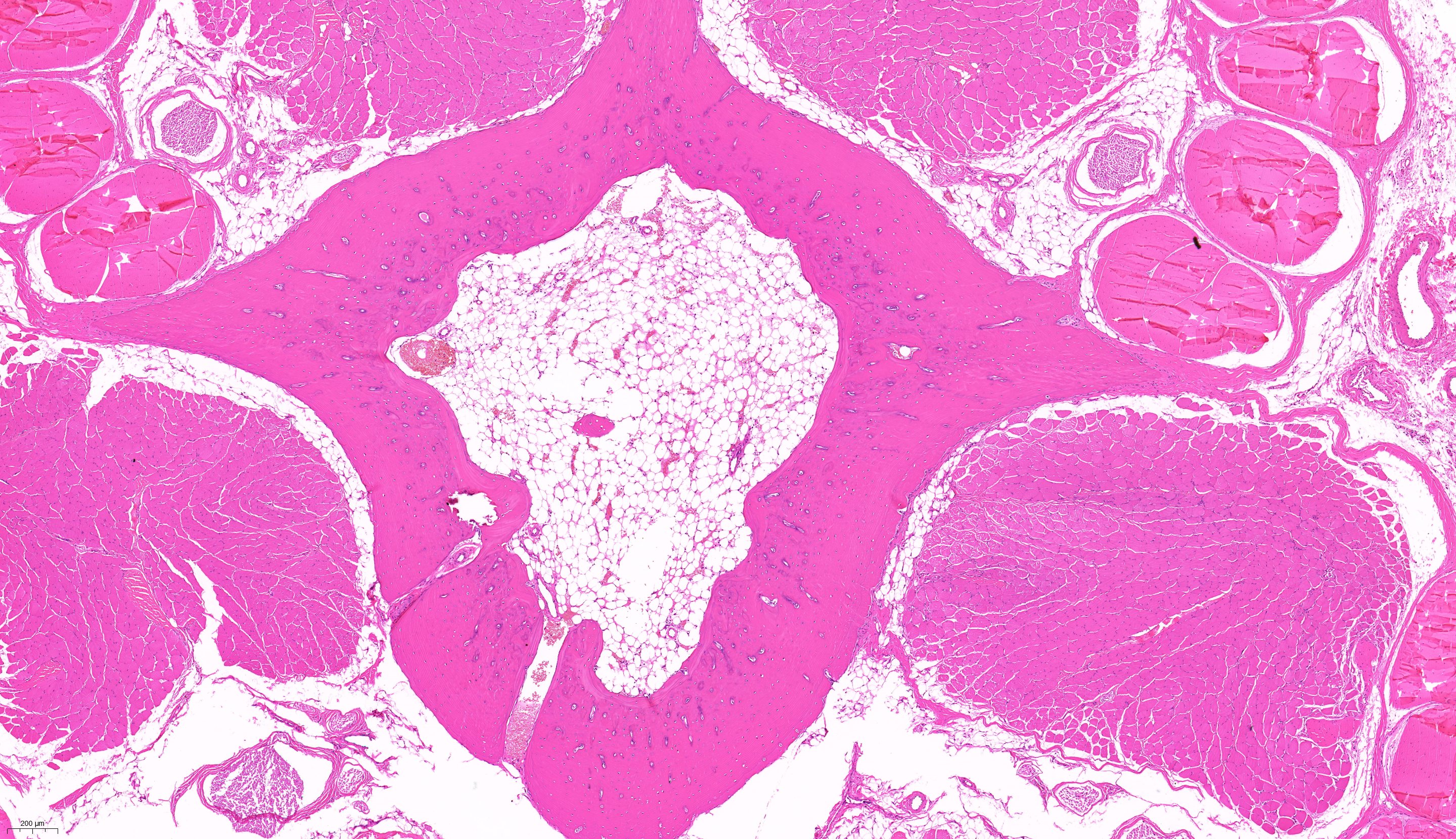

Supplement: S2 File — (ZIP) [file pone.0310014.s002.zip › HE triple section image/Control 1 HE.tif]

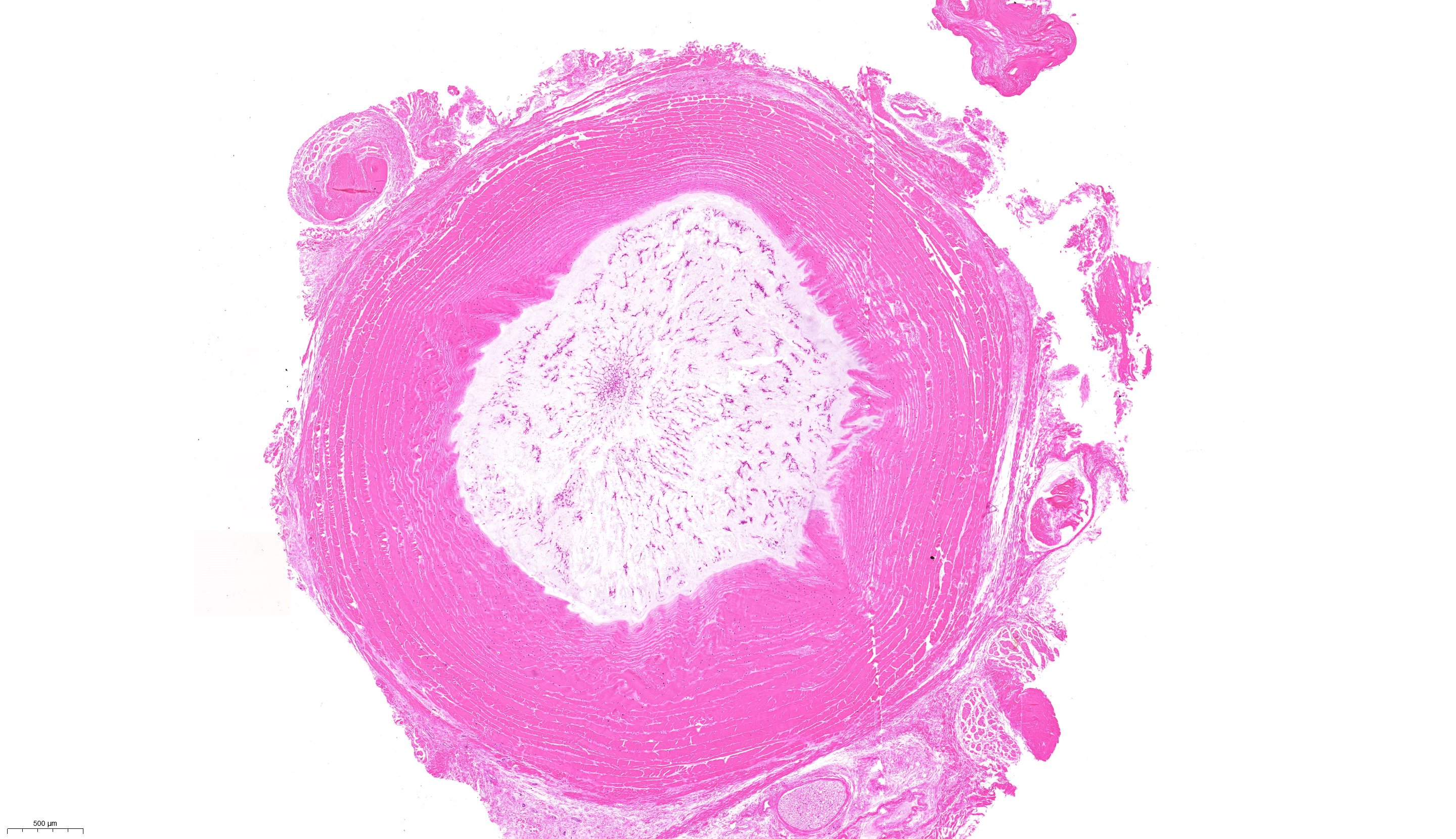

Supplement: S2 File — (ZIP) [file pone.0310014.s002.zip › HE triple section image/Control 2 HE.tif]

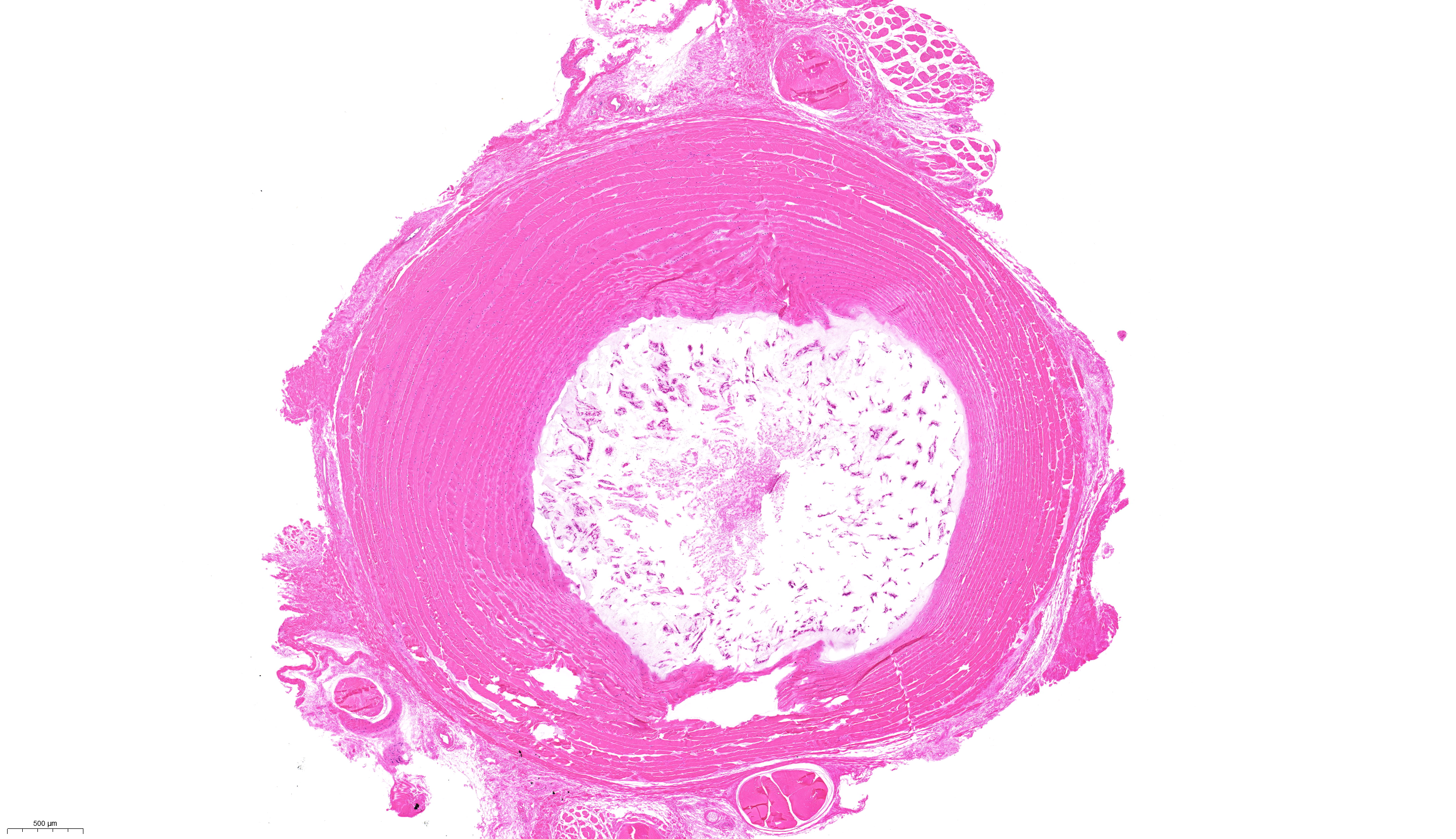

Supplement: S2 File — (ZIP) [file pone.0310014.s002.zip › HE triple section image/Control 3 HE.tif]

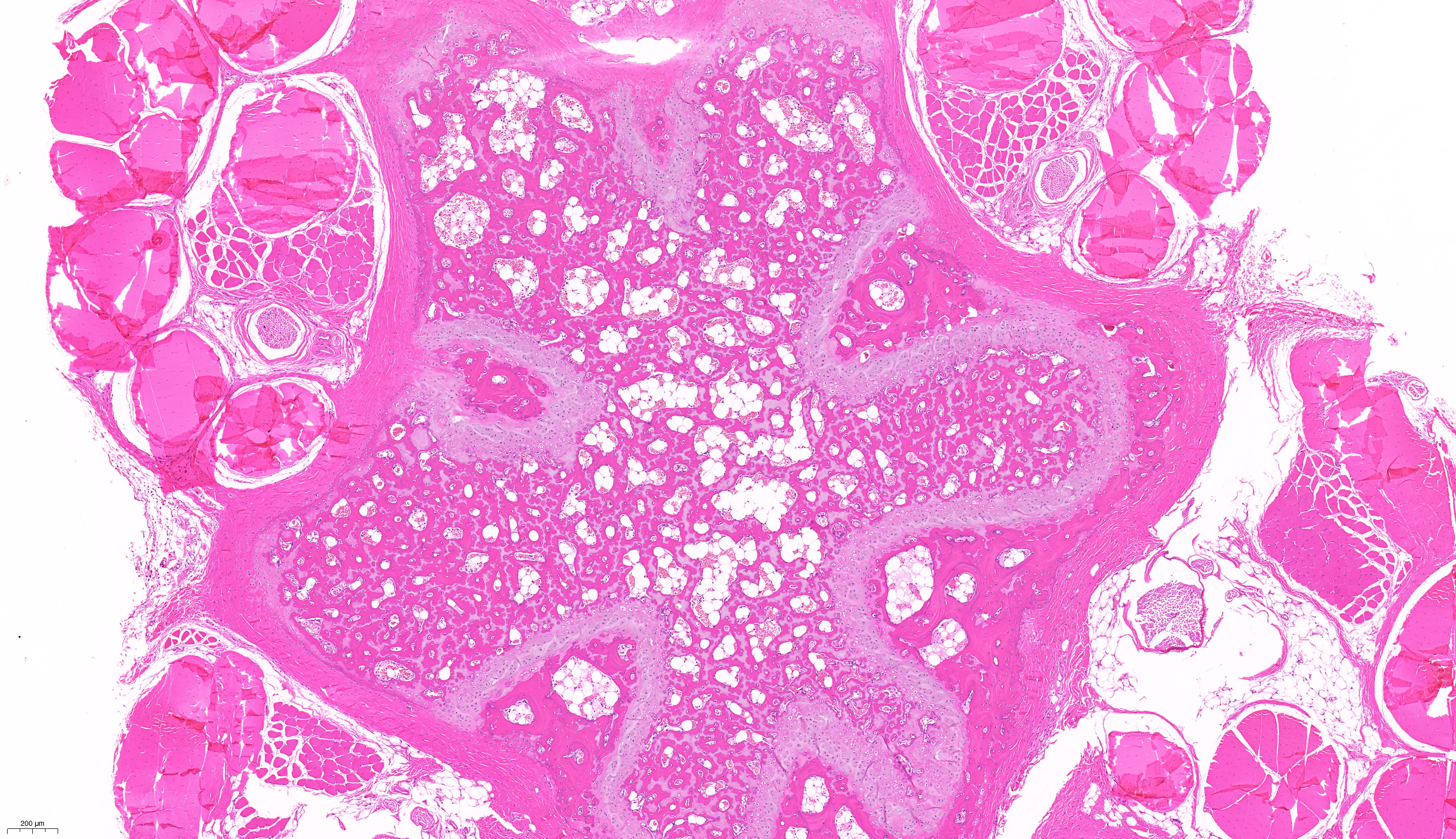

Supplement: S2 File — (ZIP) [file pone.0310014.s002.zip › HE triple section image/High1 HE.tif]

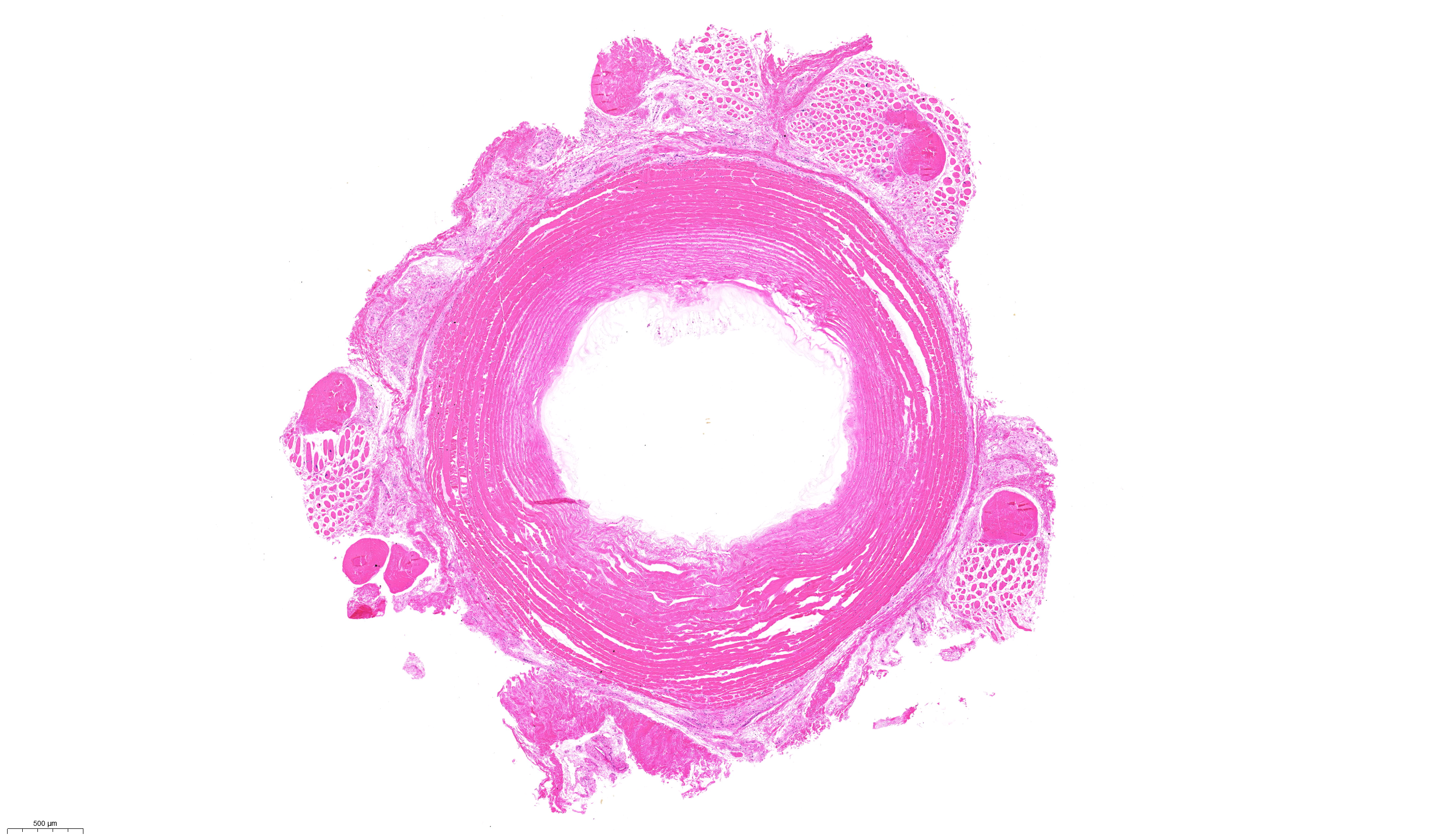

Supplement: S2 File — (ZIP) [file pone.0310014.s002.zip › HE triple section image/High2 HE.tif]

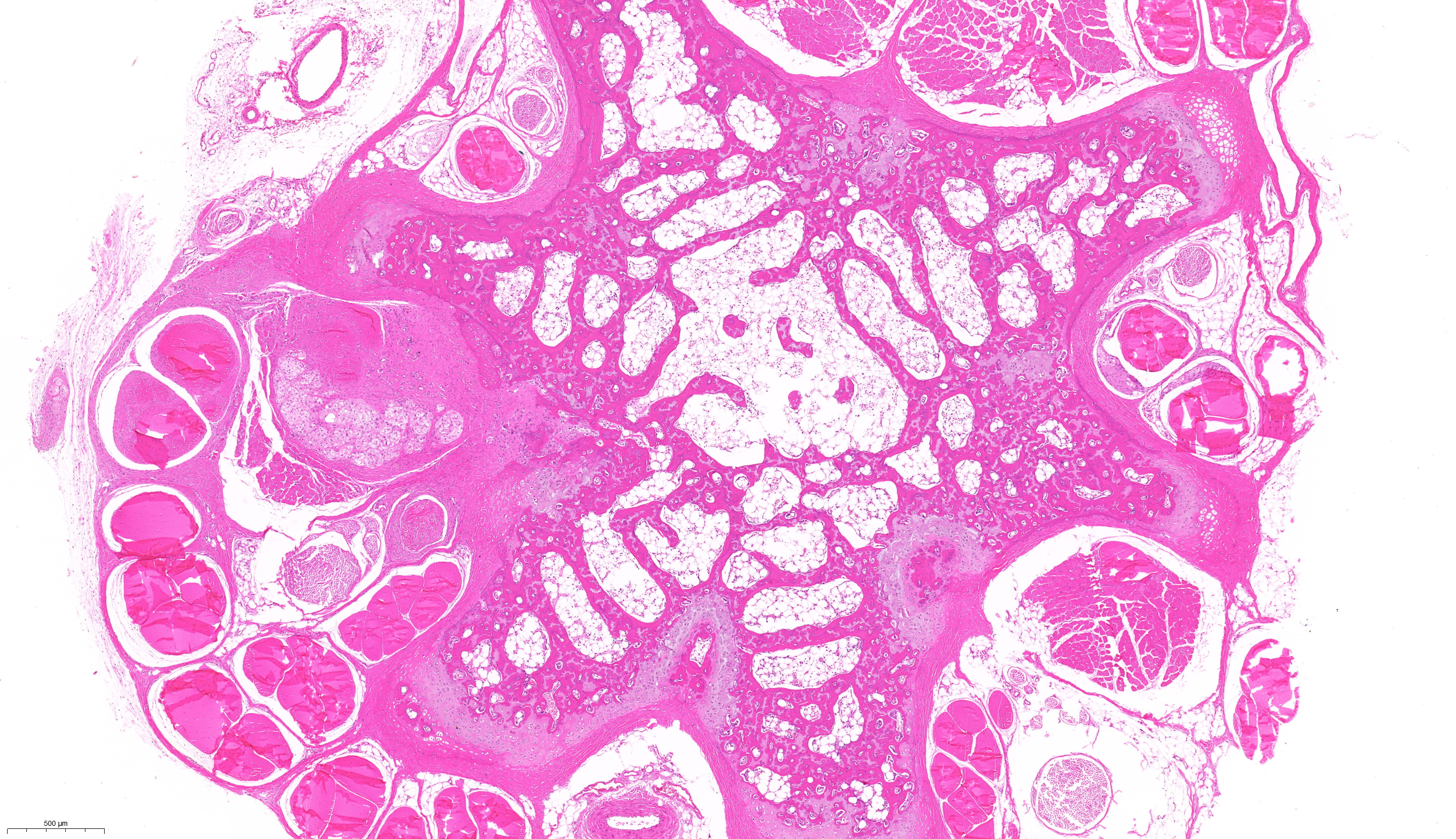

Supplement: S2 File — (ZIP) [file pone.0310014.s002.zip › HE triple section image/Low1 HE.tif]

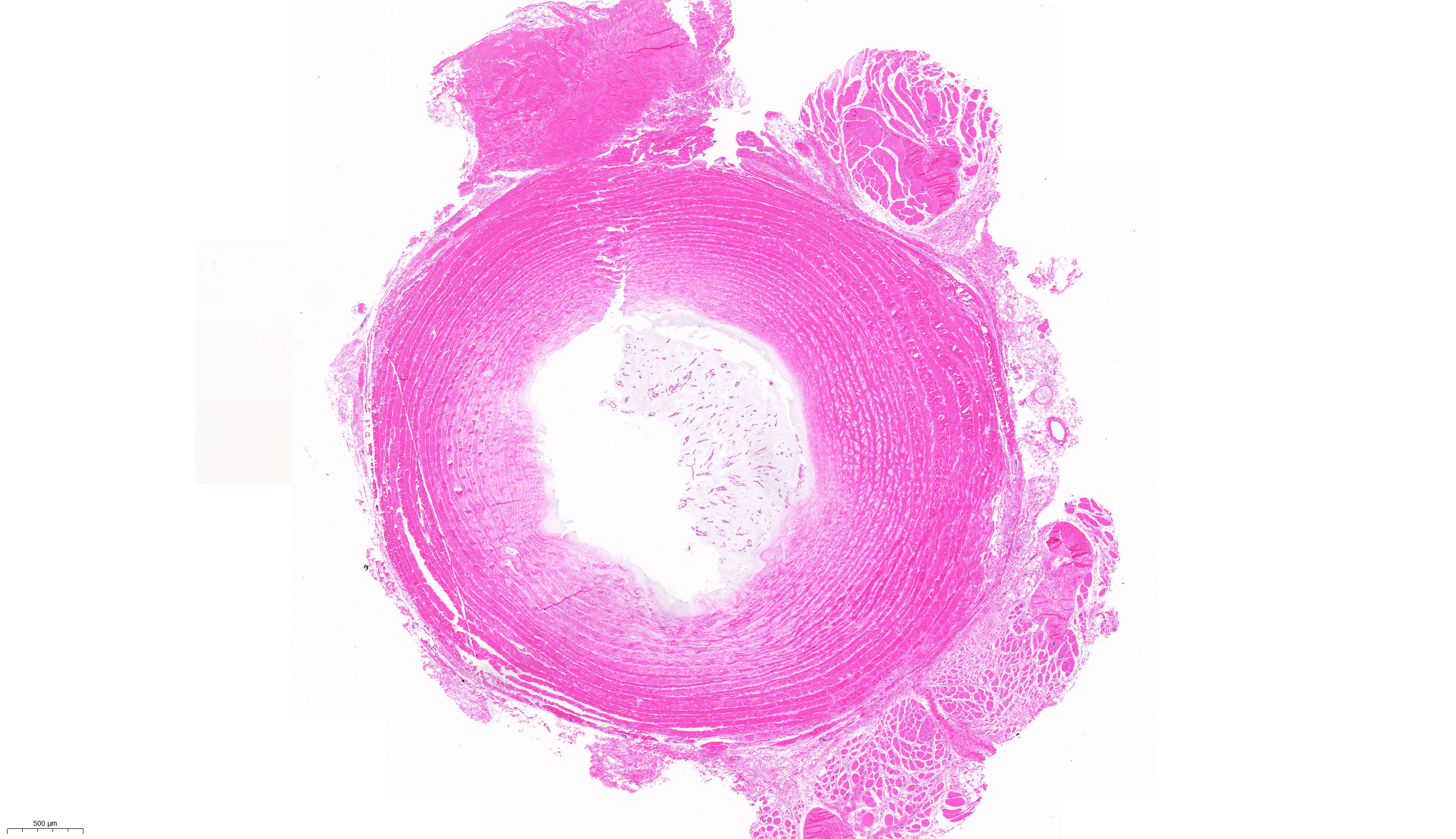

Supplement: S2 File — (ZIP) [file pone.0310014.s002.zip › HE triple section image/Low2 HE.tif]

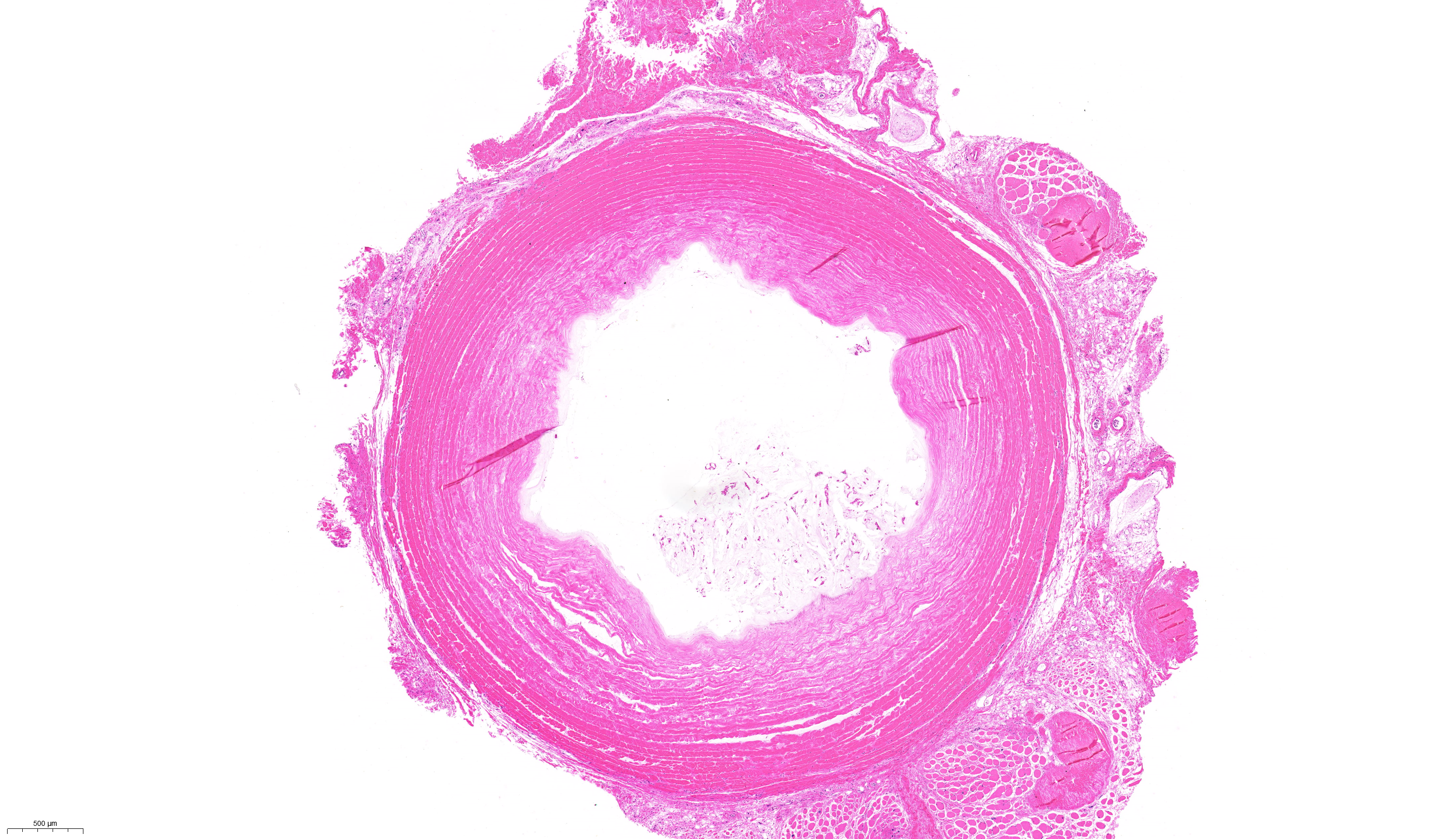

Supplement: S2 File — (ZIP) [file pone.0310014.s002.zip › HE triple section image/LOW3 HE.tif]

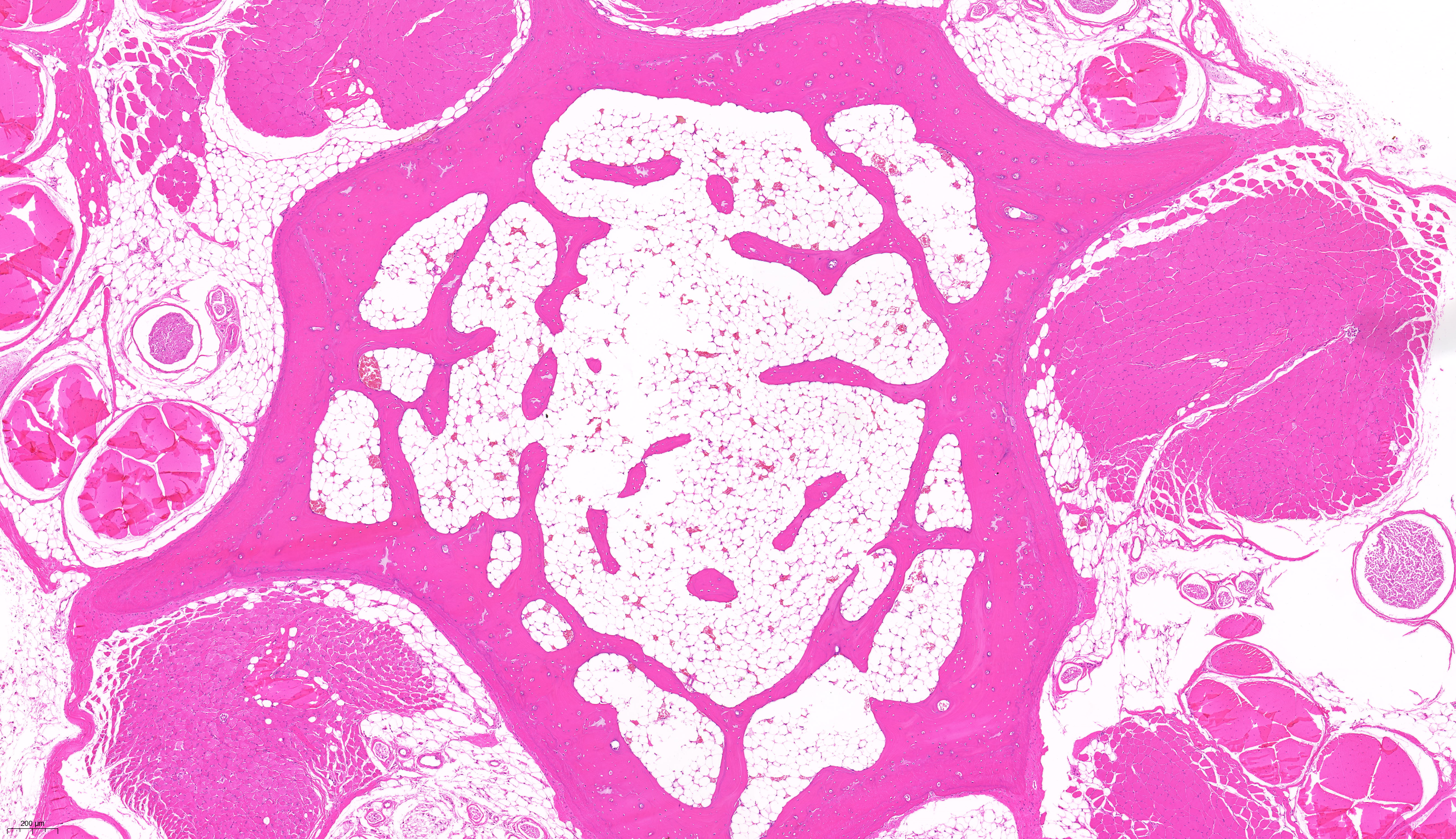

Supplement: S2 File — (ZIP) [file pone.0310014.s002.zip › HE triple section image/Medium 1 HE.tif]

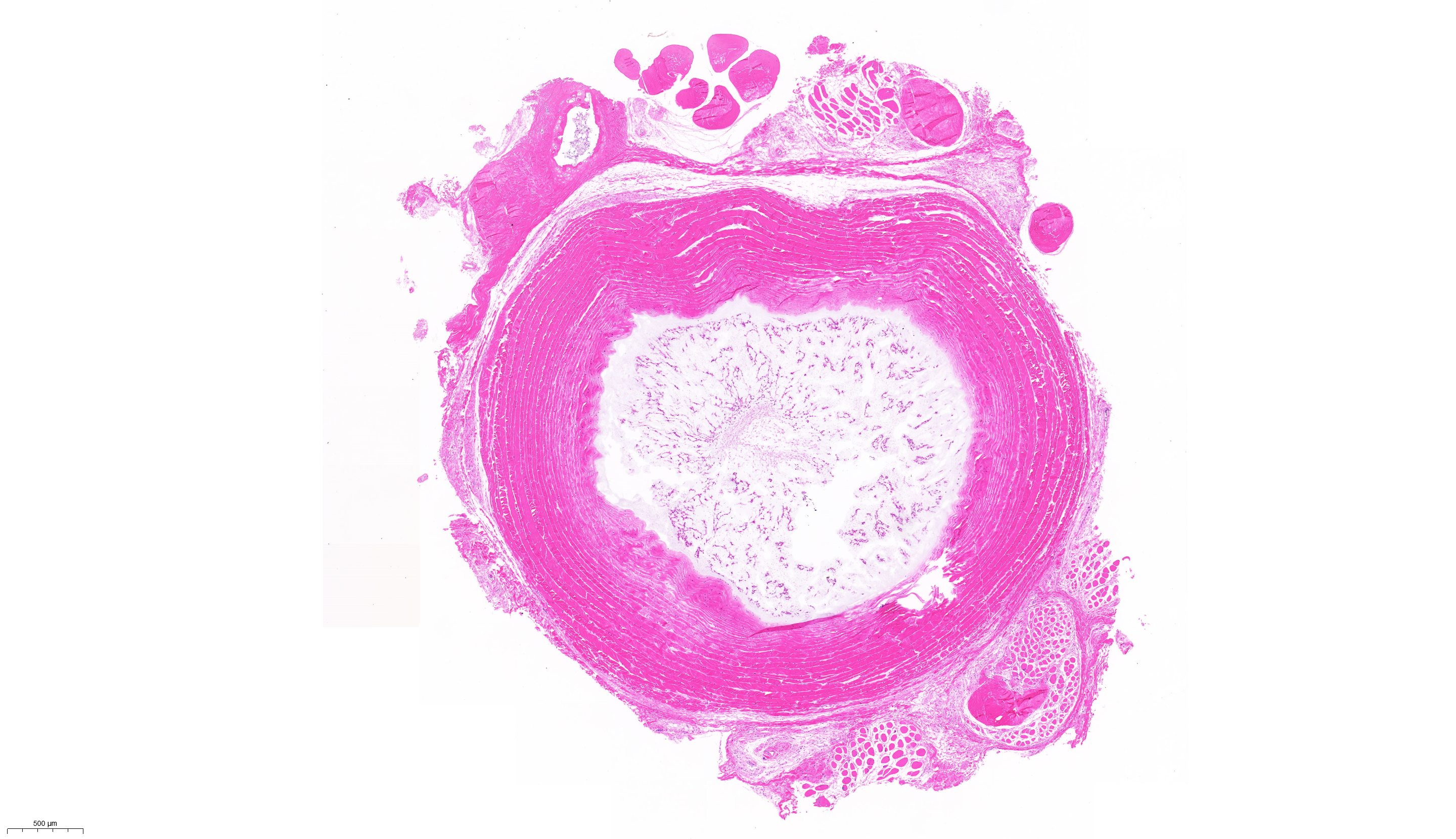

Supplement: S2 File — (ZIP) [file pone.0310014.s002.zip › HE triple section image/Medium 2 HE.tif]

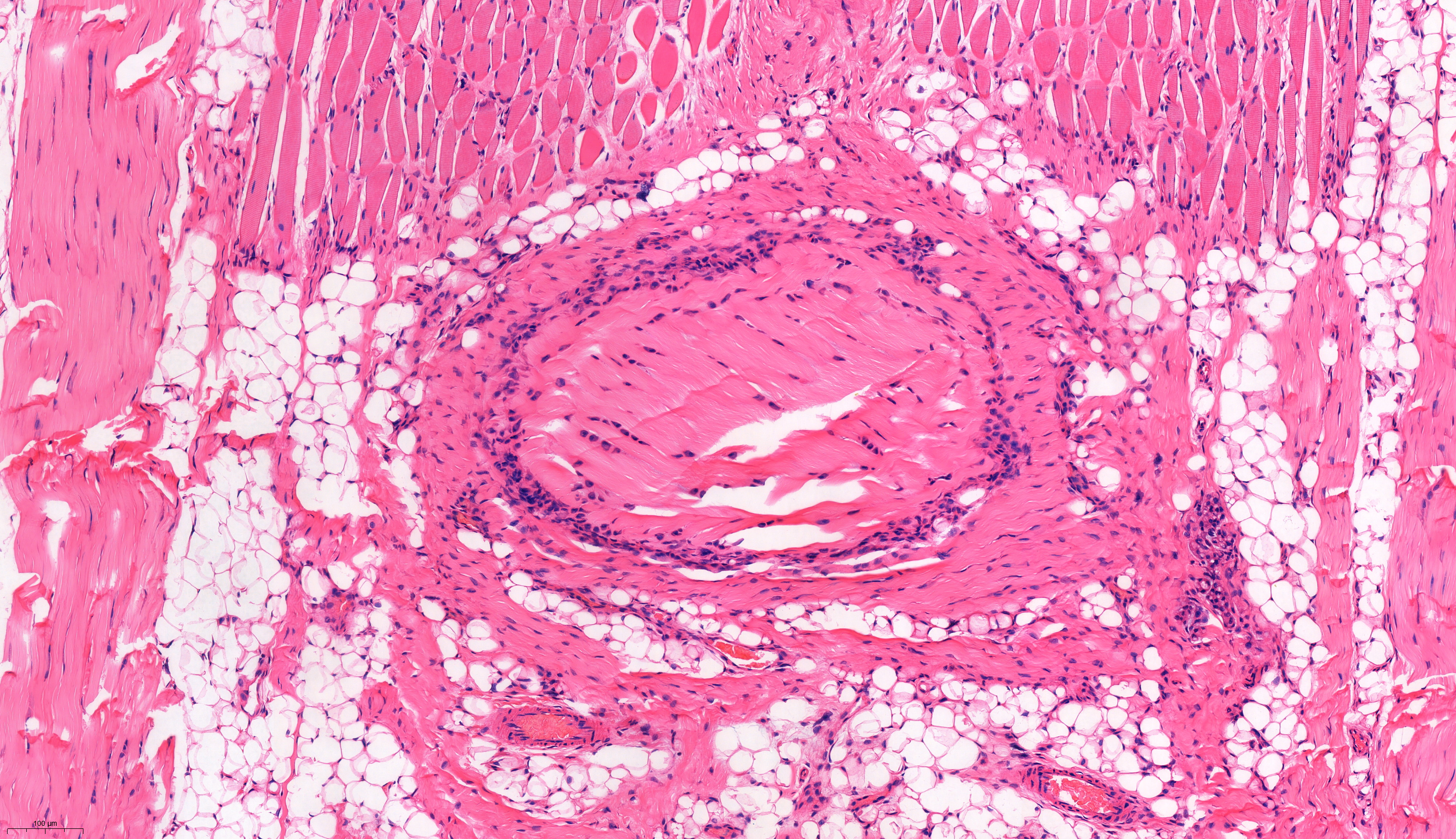

Supplement: S2 File — (ZIP) [file pone.0310014.s002.zip › HE triple section image/Model 1 HE.tif]

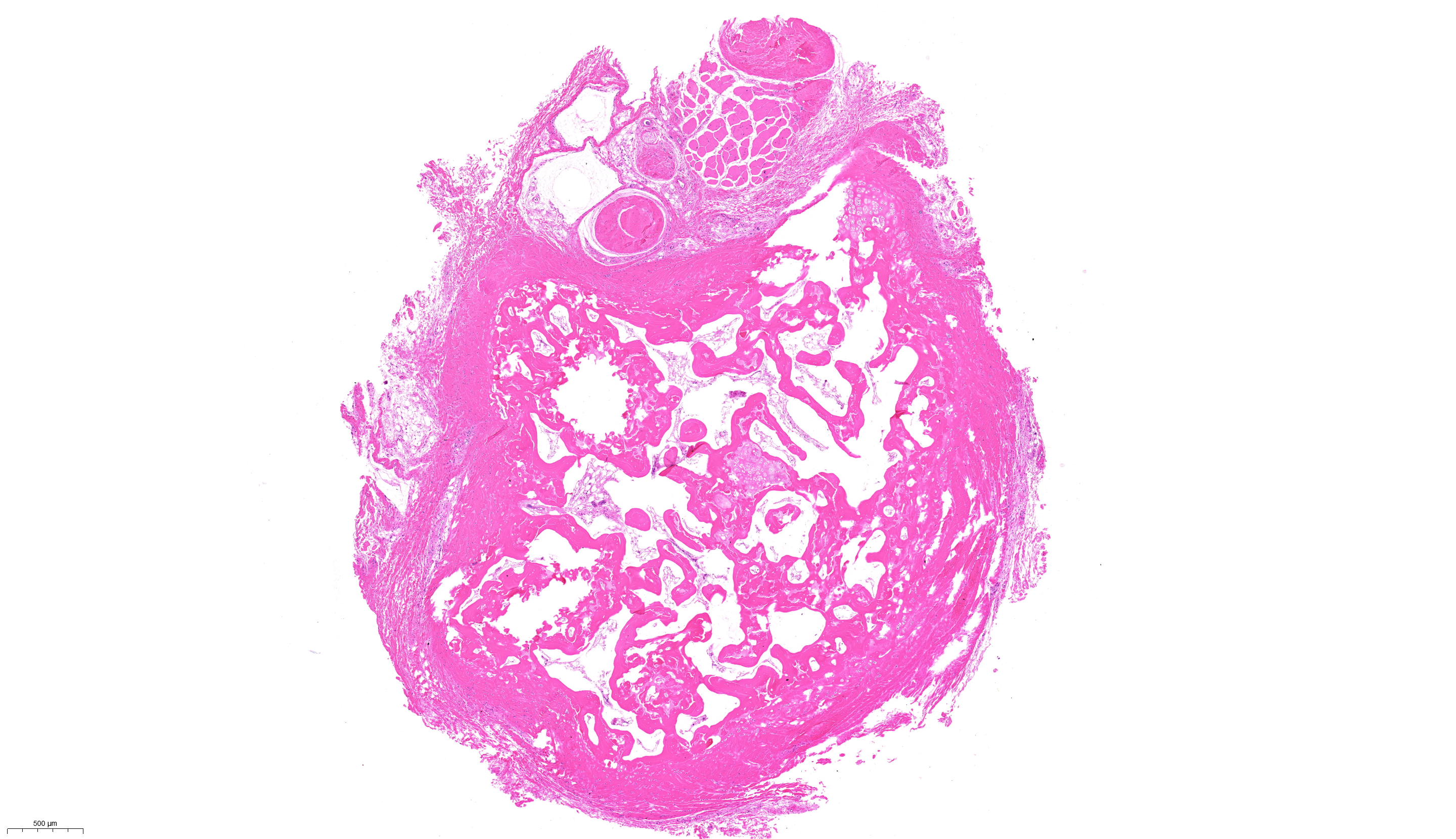

Supplement: S2 File — (ZIP) [file pone.0310014.s002.zip › HE triple section image/Model2 HE.tif]

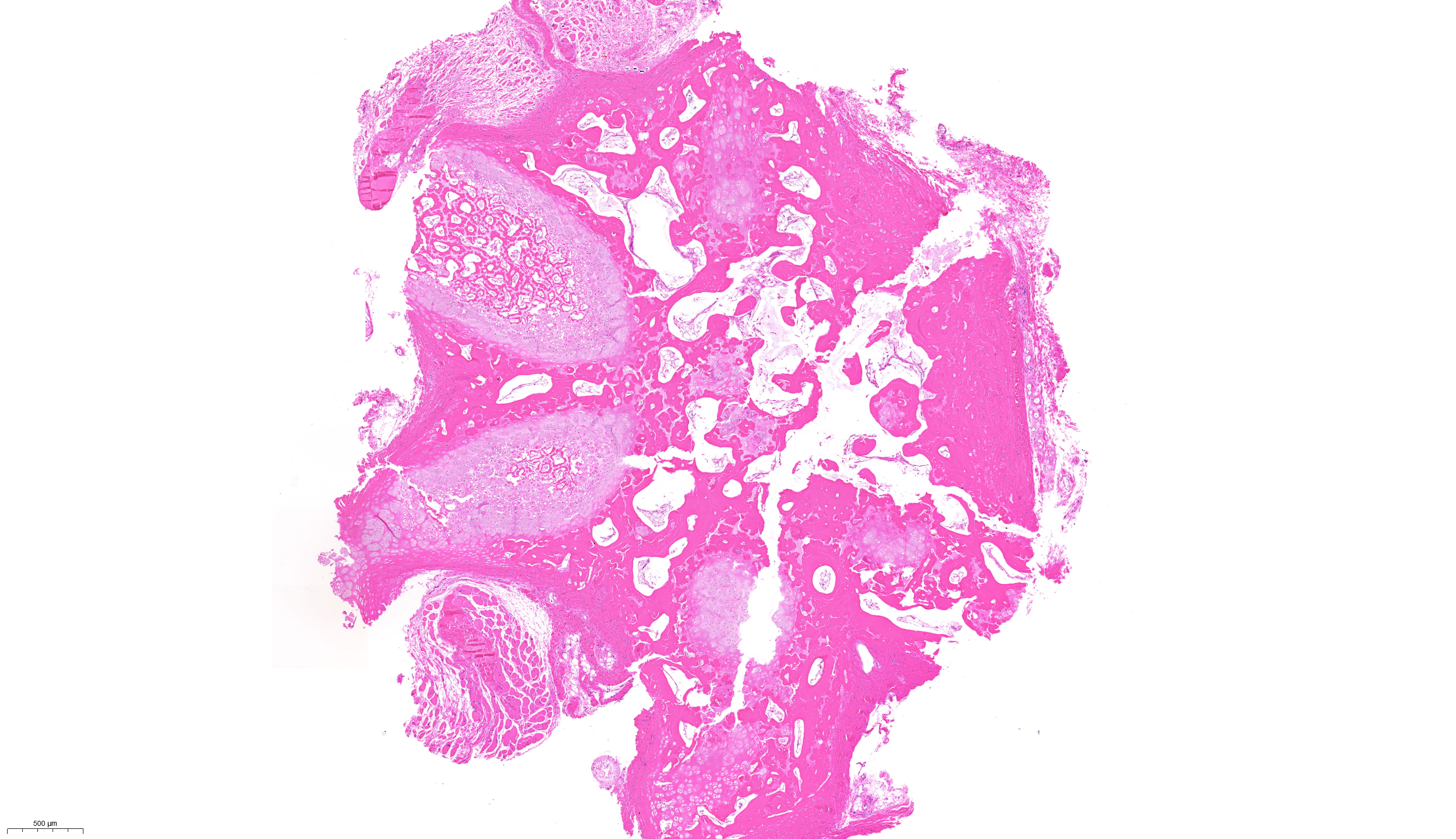

Supplement: S2 File — (ZIP) [file pone.0310014.s002.zip › HE triple section image/Model3 HE.tif]

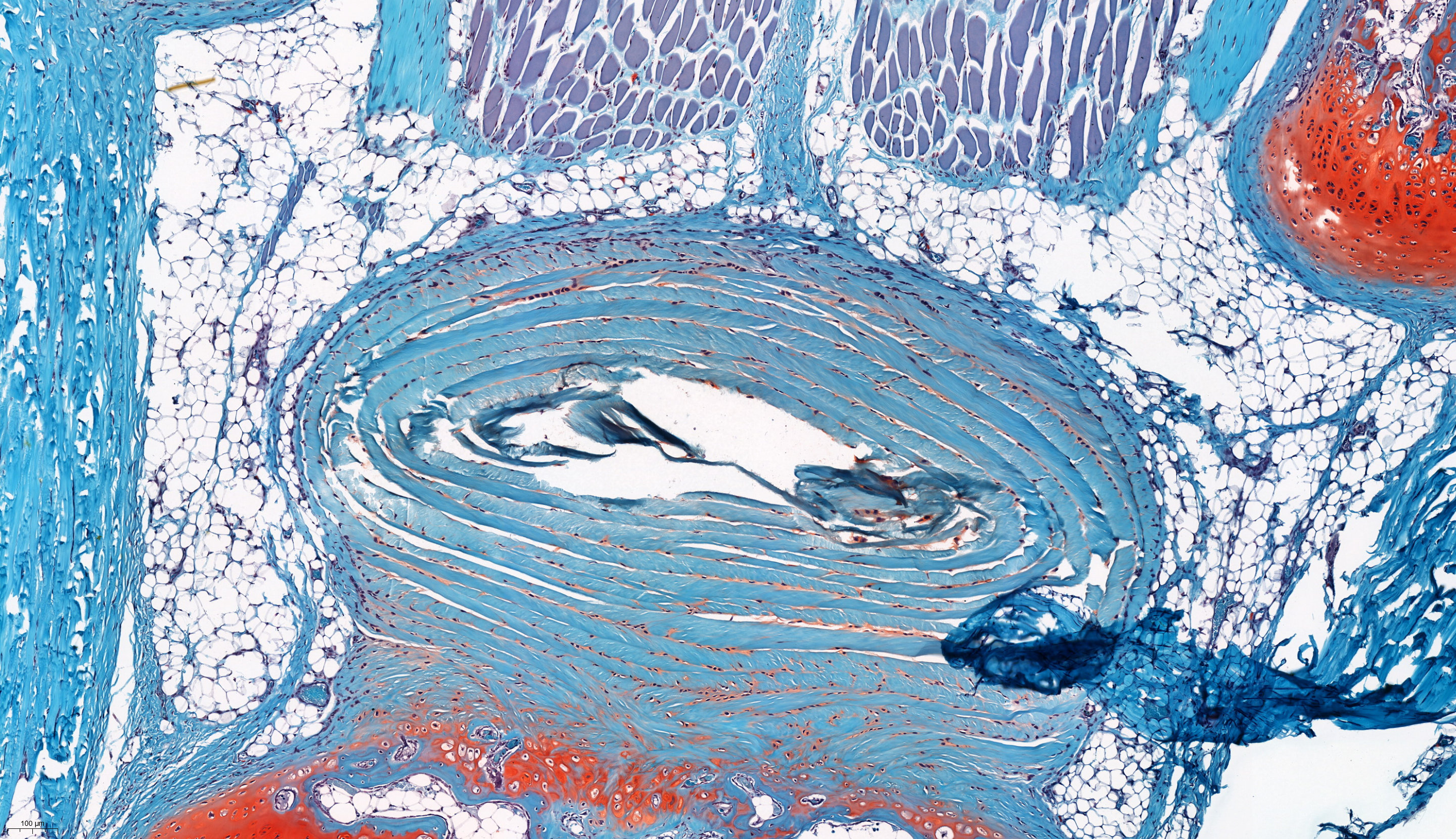

Supplement: S3 File — (ZIP) [file pone.0310014.s003.zip › SOFG triple section image/Celecoxib1 SOFG.tif]

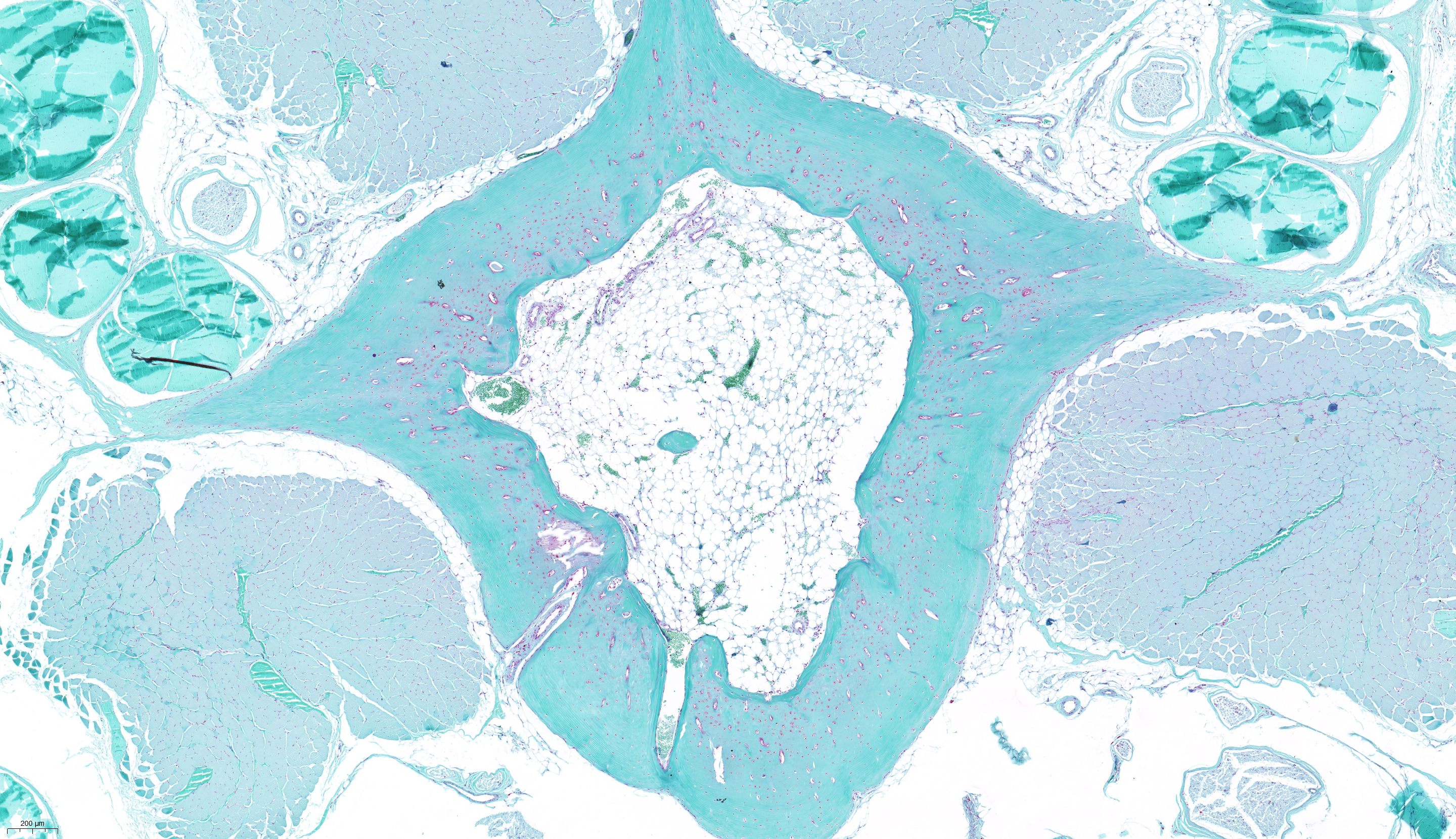

Supplement: S3 File — (ZIP) [file pone.0310014.s003.zip › SOFG triple section image/Control 1 SOFG.tif]

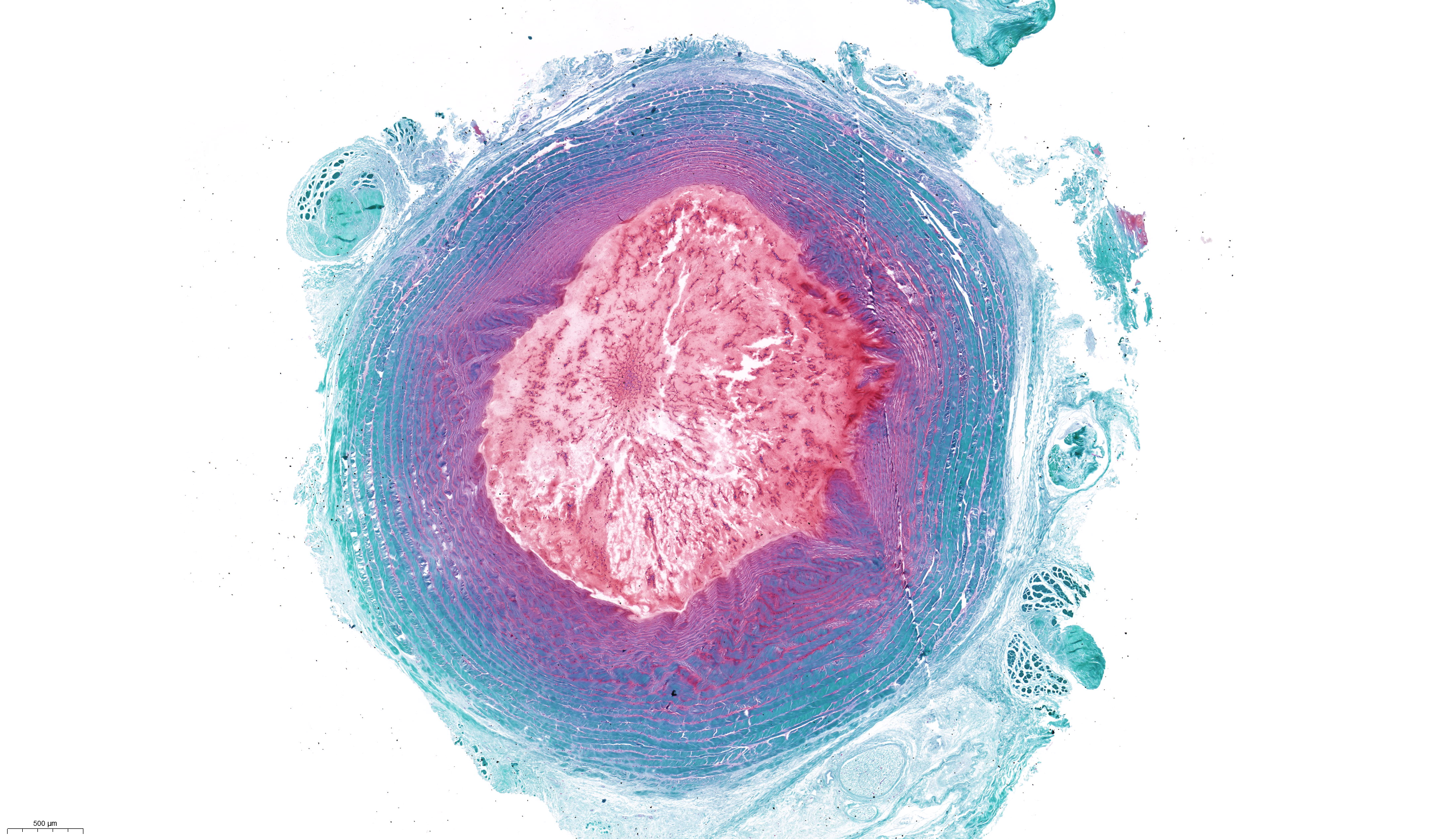

Supplement: S3 File — (ZIP) [file pone.0310014.s003.zip › SOFG triple section image/Control 2 SOFG.tif]

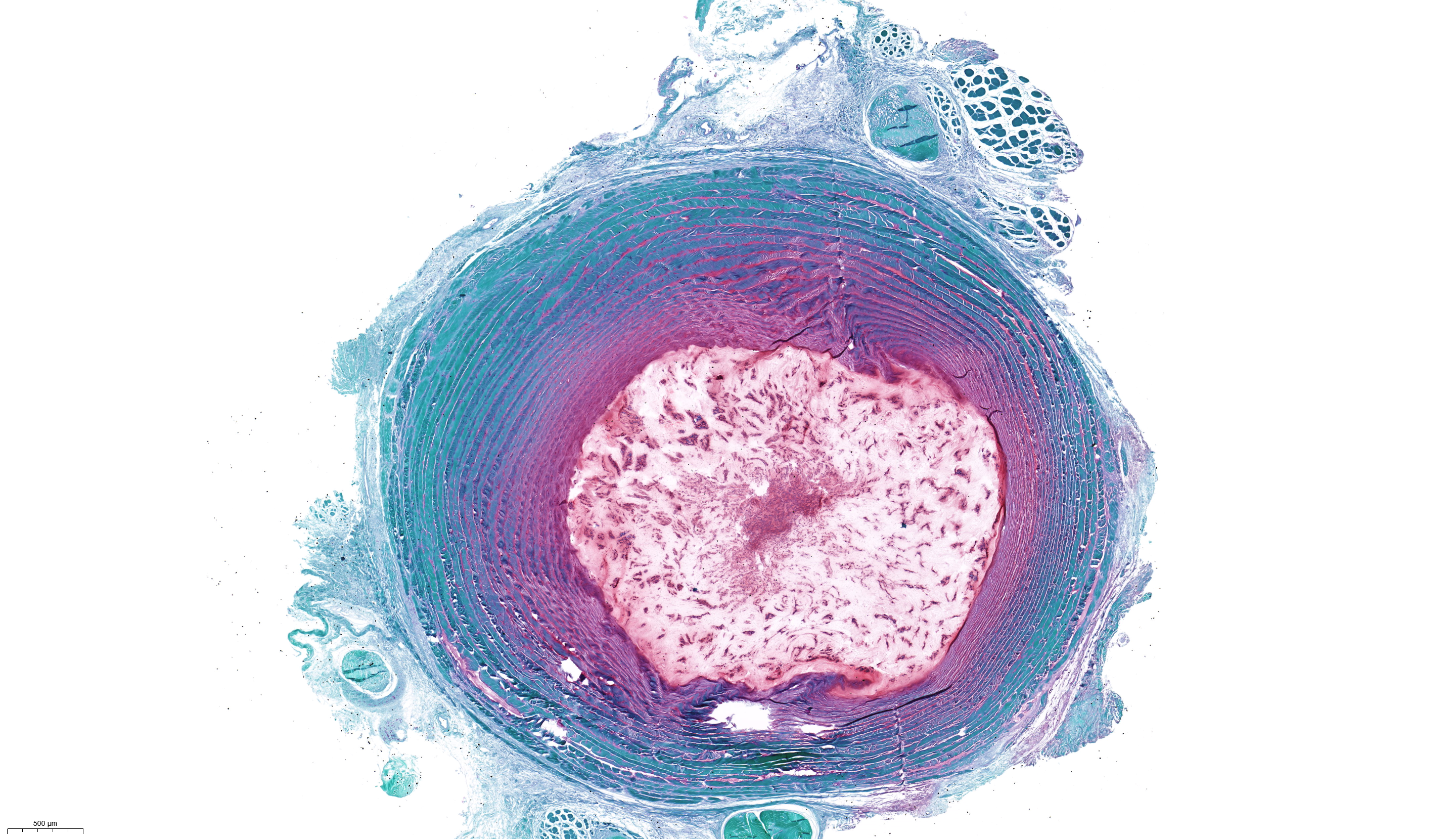

Supplement: S3 File — (ZIP) [file pone.0310014.s003.zip › SOFG triple section image/Control 3 SOFG.tif]

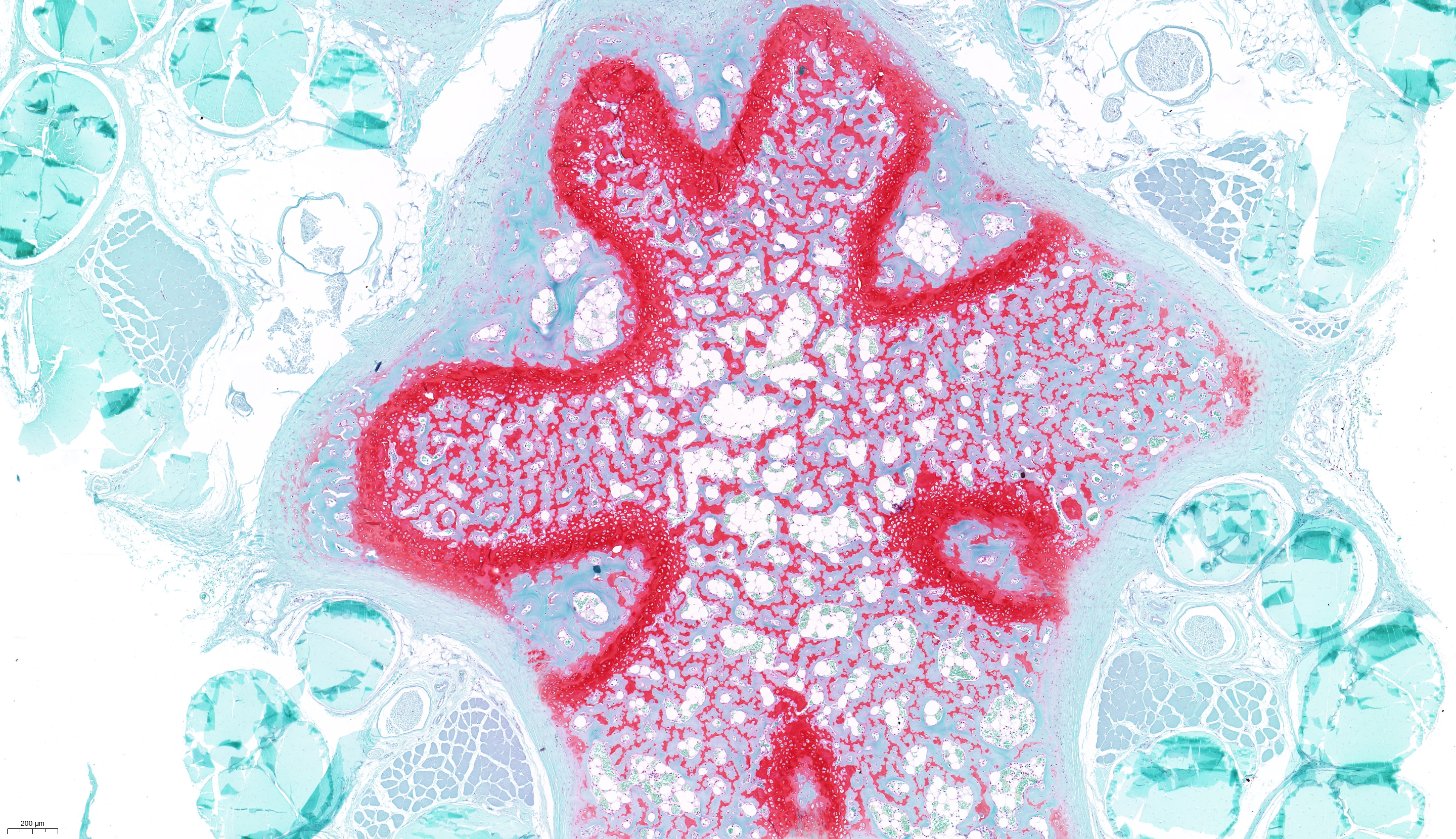

Supplement: S3 File — (ZIP) [file pone.0310014.s003.zip › SOFG triple section image/High1 SOFG.tif]

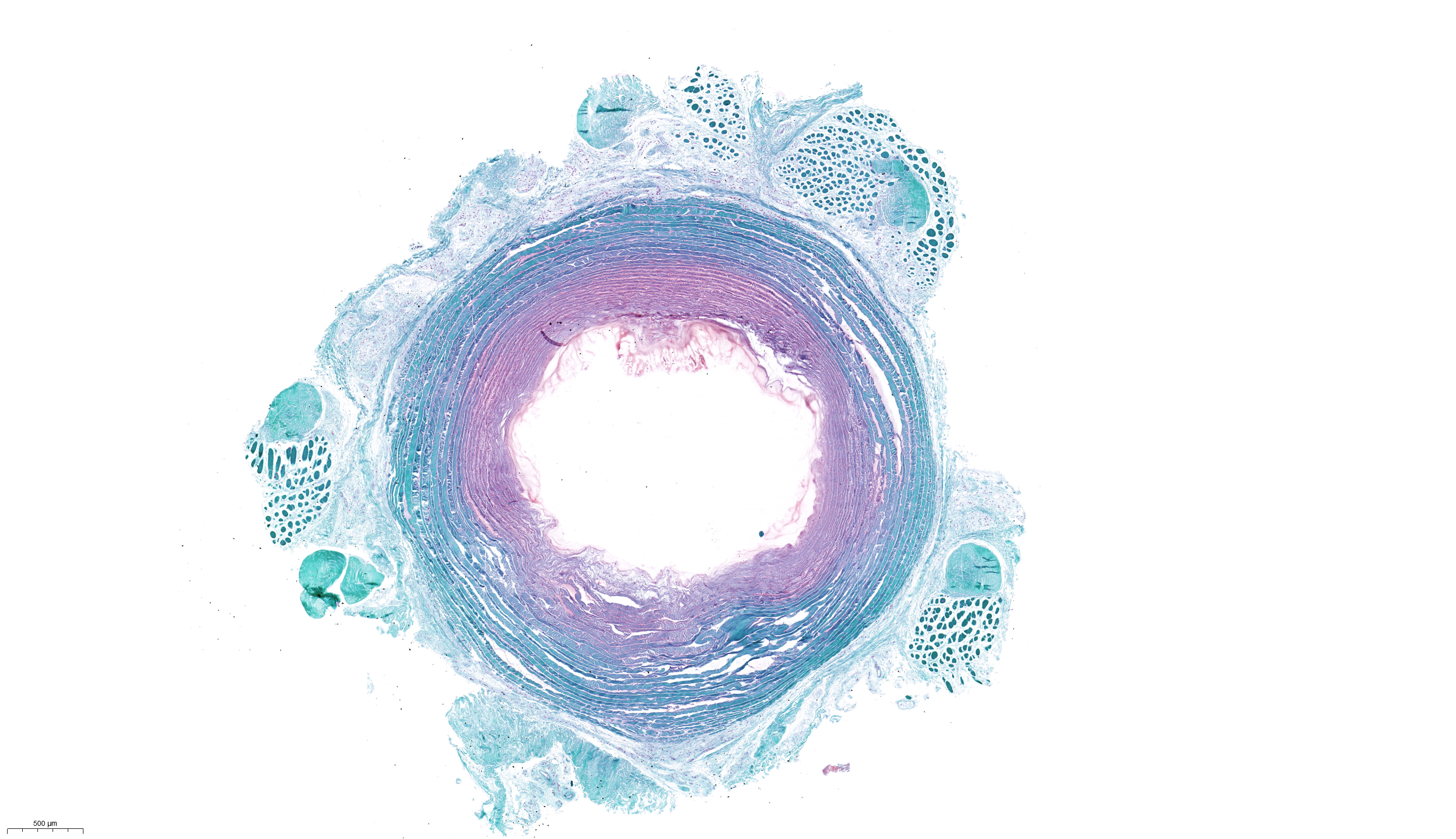

Supplement: S3 File — (ZIP) [file pone.0310014.s003.zip › SOFG triple section image/High2 SOFG.tif]

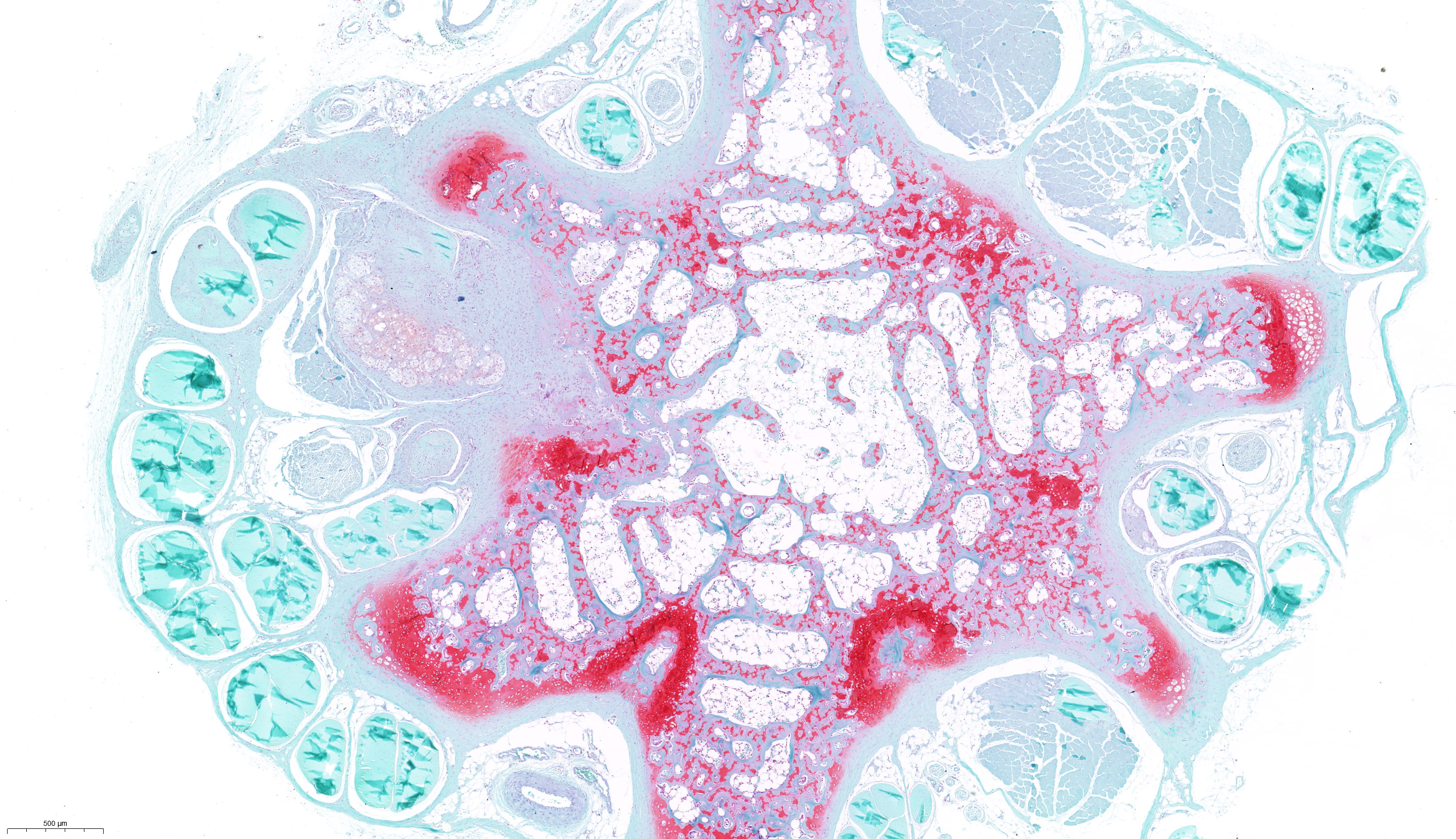

Supplement: S3 File — (ZIP) [file pone.0310014.s003.zip › SOFG triple section image/Low1 SOFG.tif]

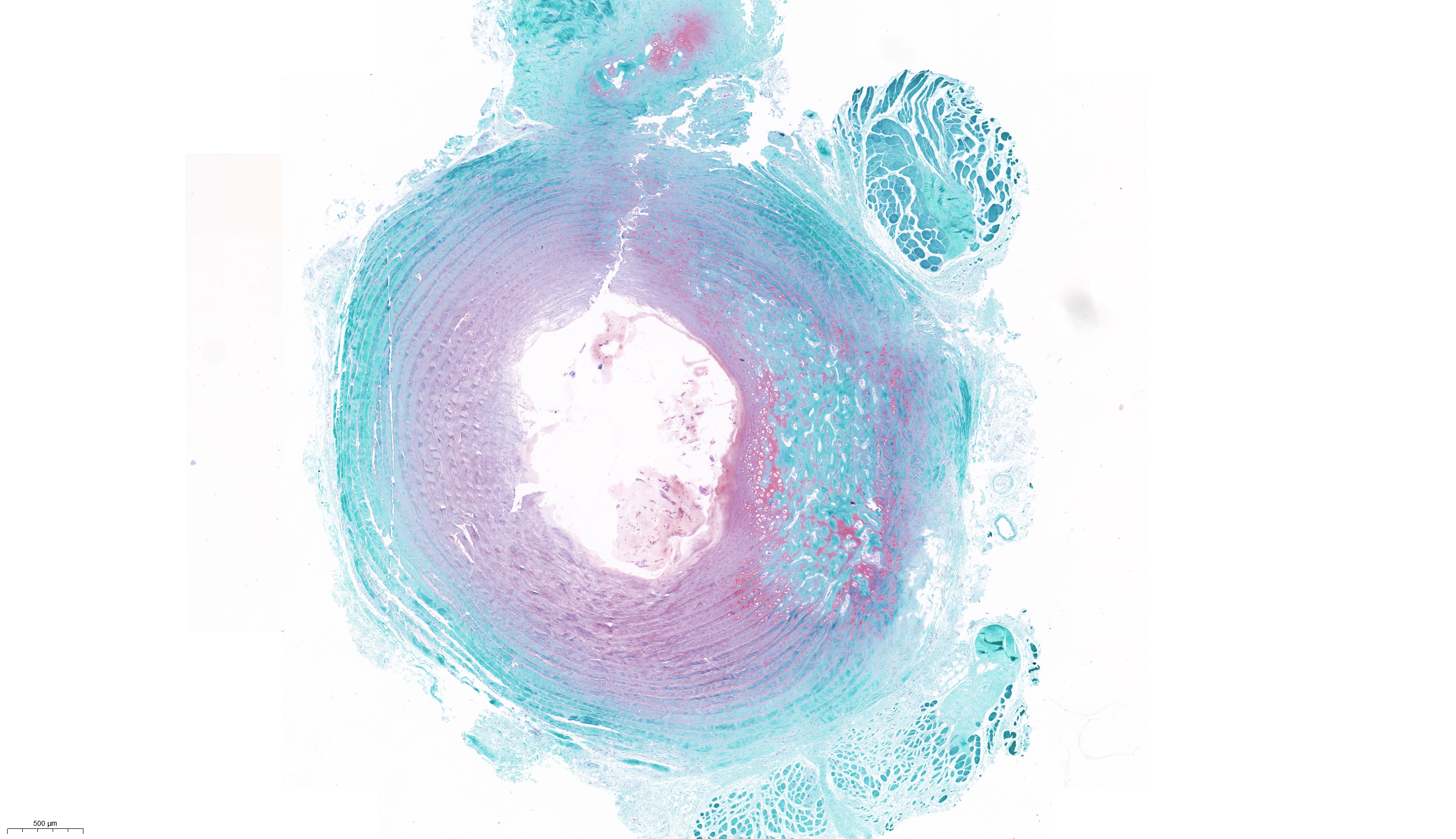

Supplement: S3 File — (ZIP) [file pone.0310014.s003.zip › SOFG triple section image/Low2 SOFG.tif]

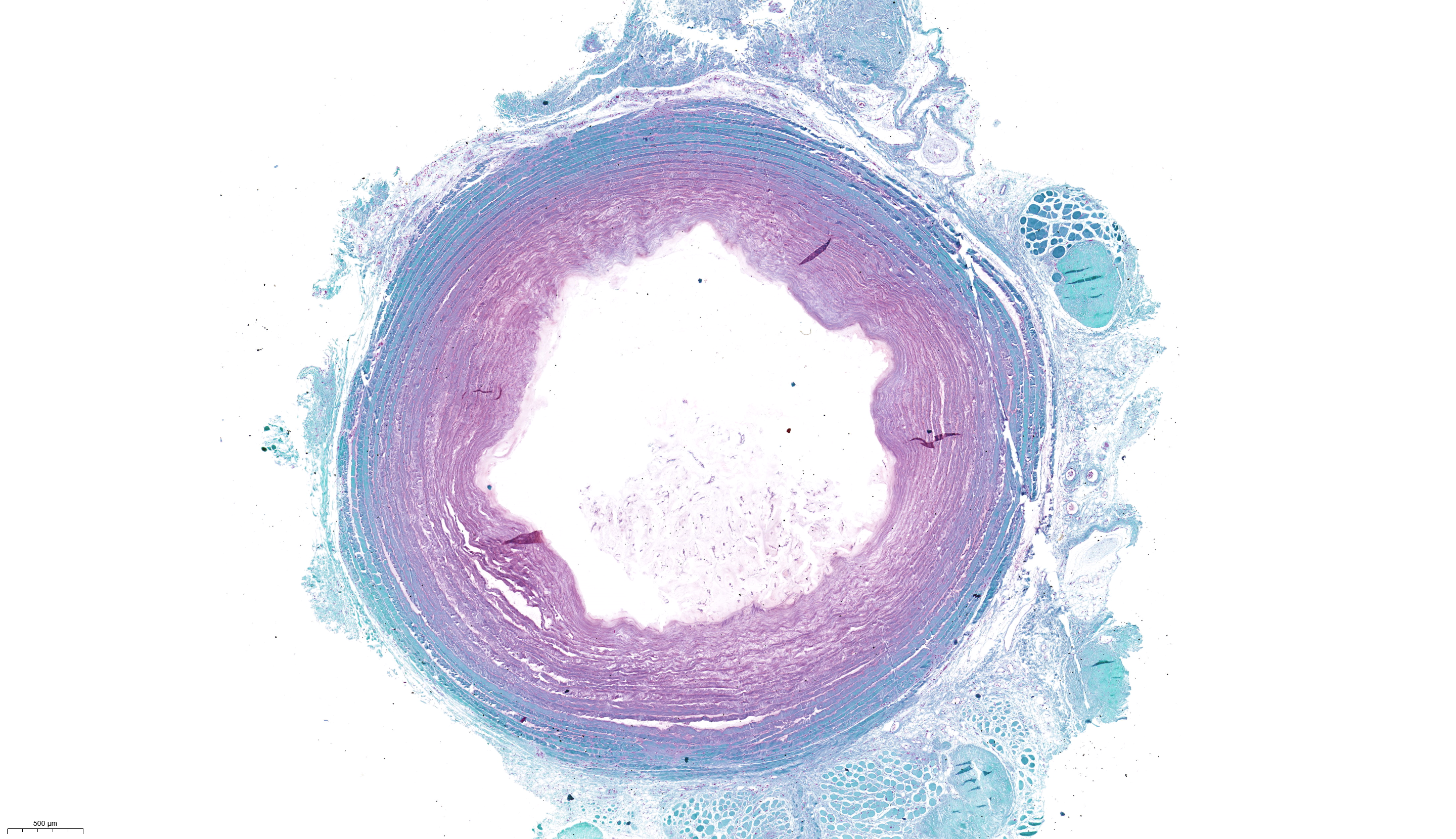

Supplement: S3 File — (ZIP) [file pone.0310014.s003.zip › SOFG triple section image/LOW3 SOFG.tif]

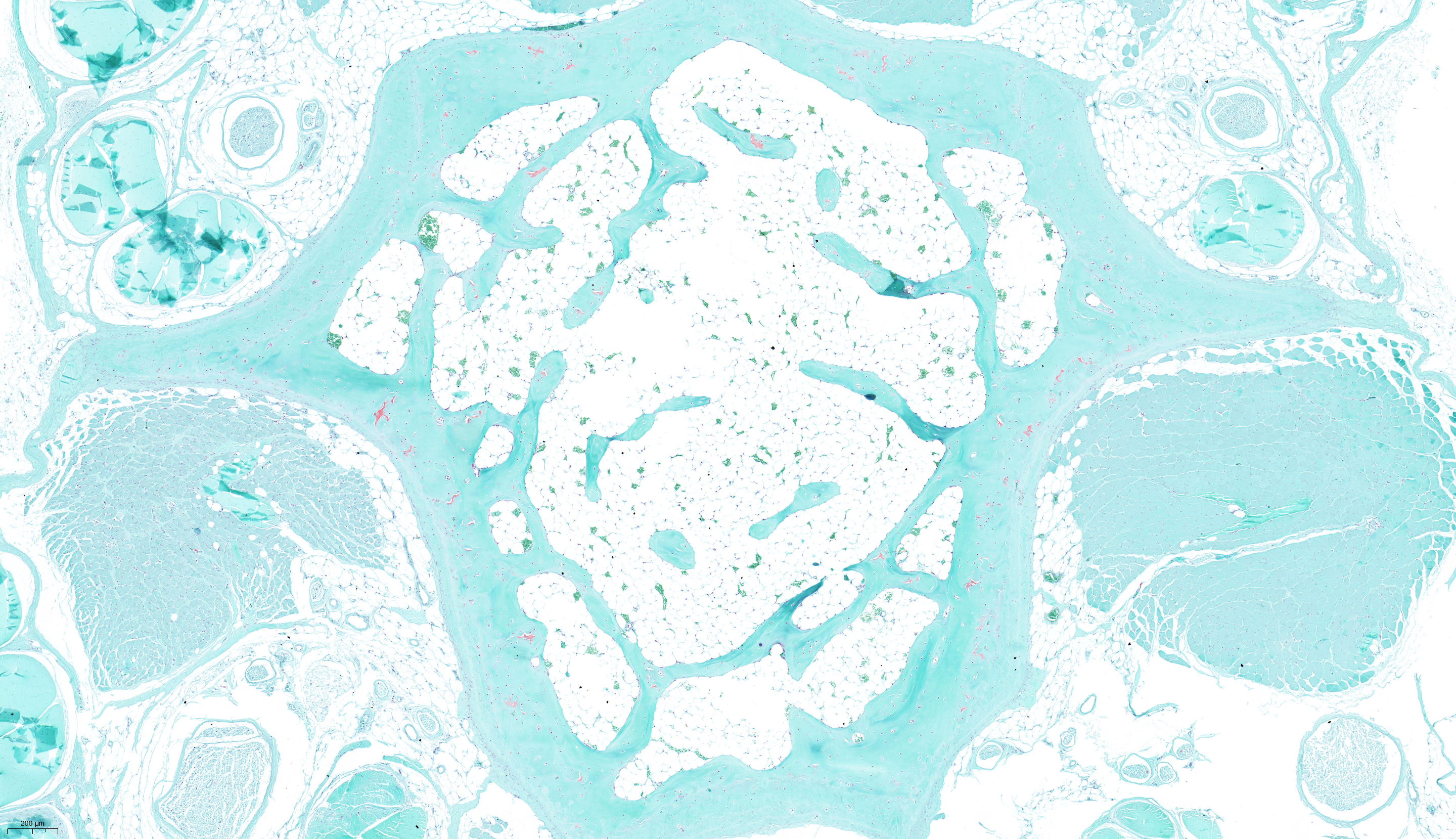

Supplement: S3 File — (ZIP) [file pone.0310014.s003.zip › SOFG triple section image/Medium 1 SOFG.tif]

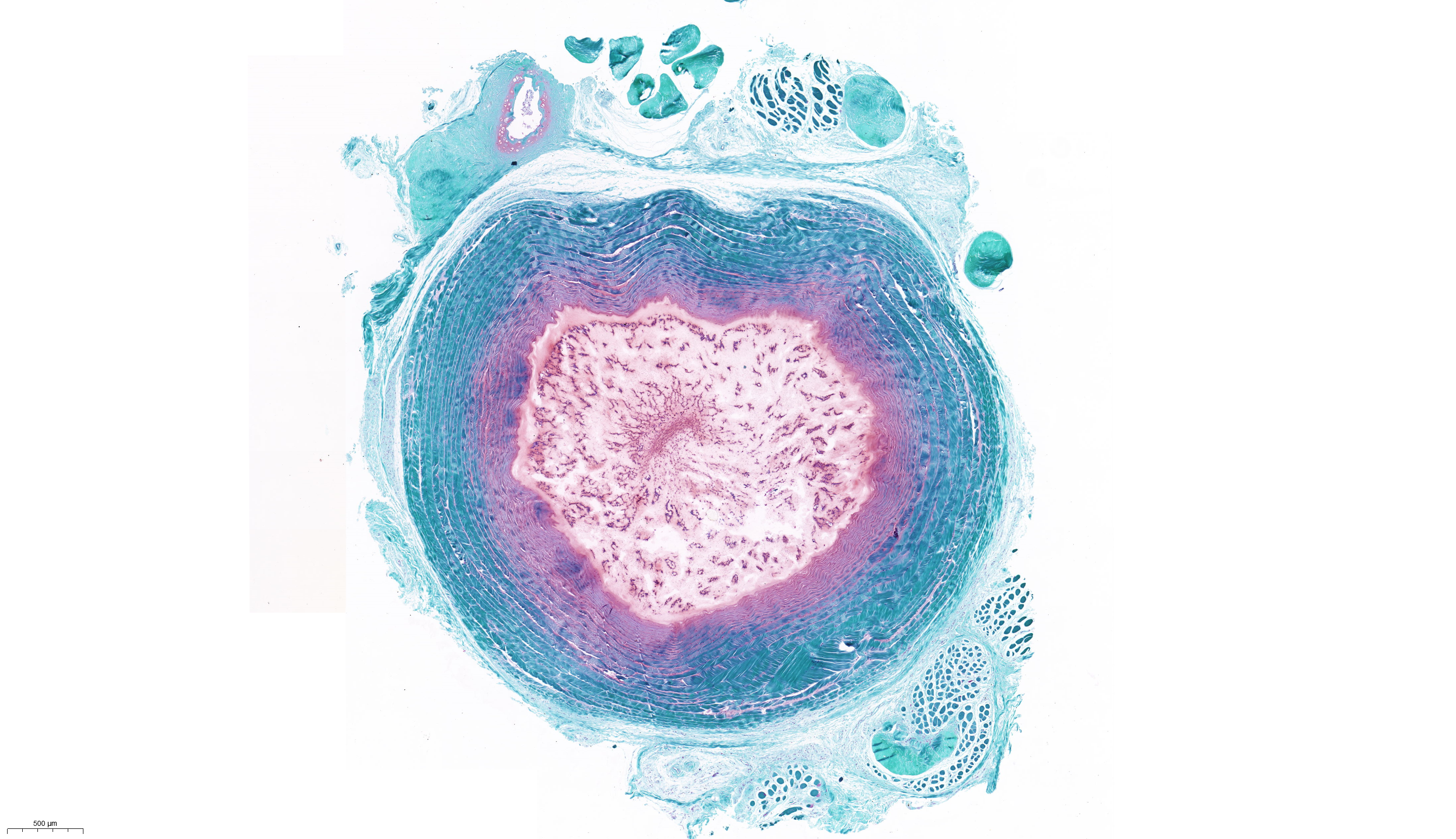

Supplement: S3 File — (ZIP) [file pone.0310014.s003.zip › SOFG triple section image/Medium 2 SOFG.tif]

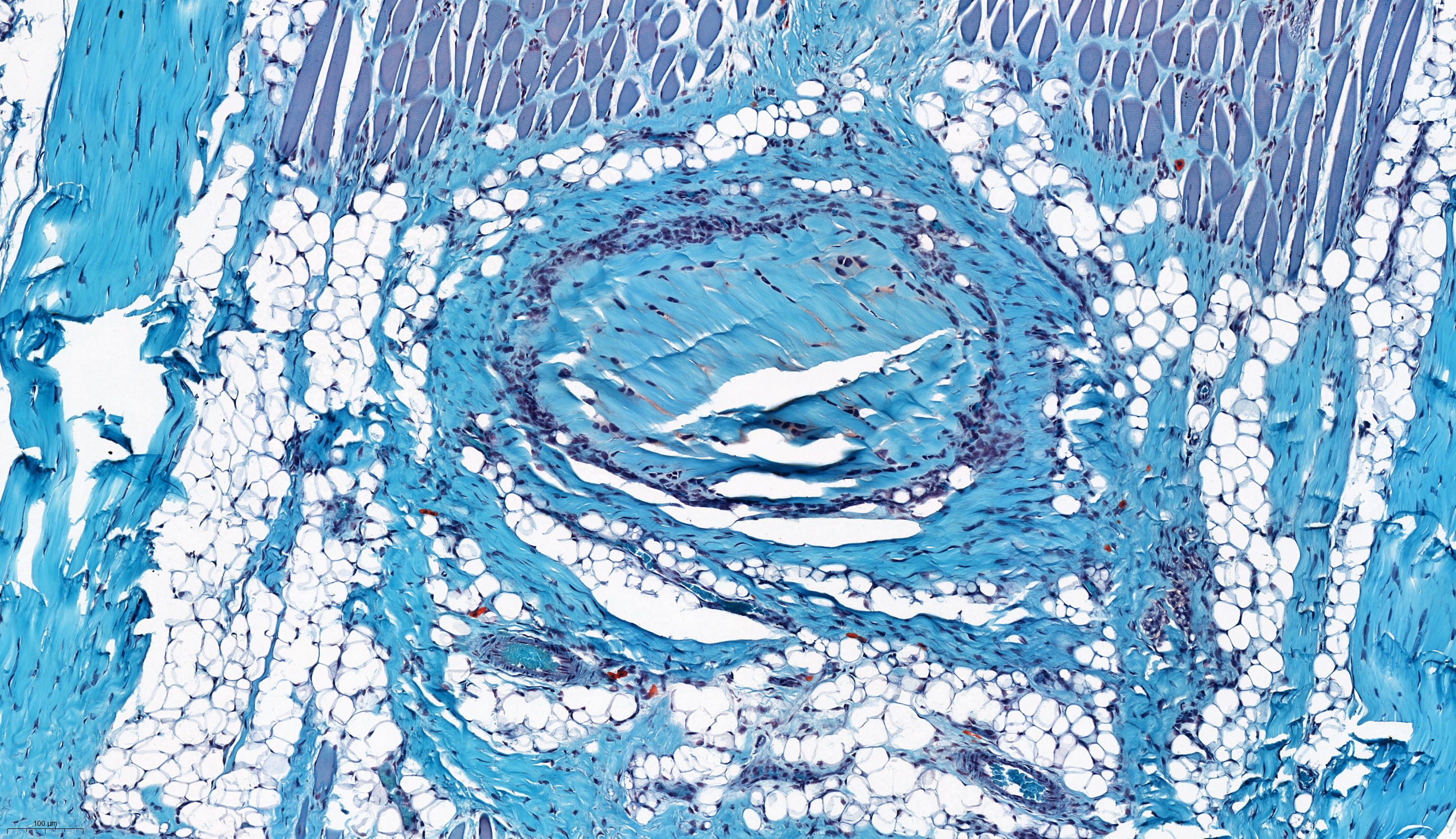

Supplement: S3 File — (ZIP) [file pone.0310014.s003.zip › SOFG triple section image/Model1 SOFG.tif]

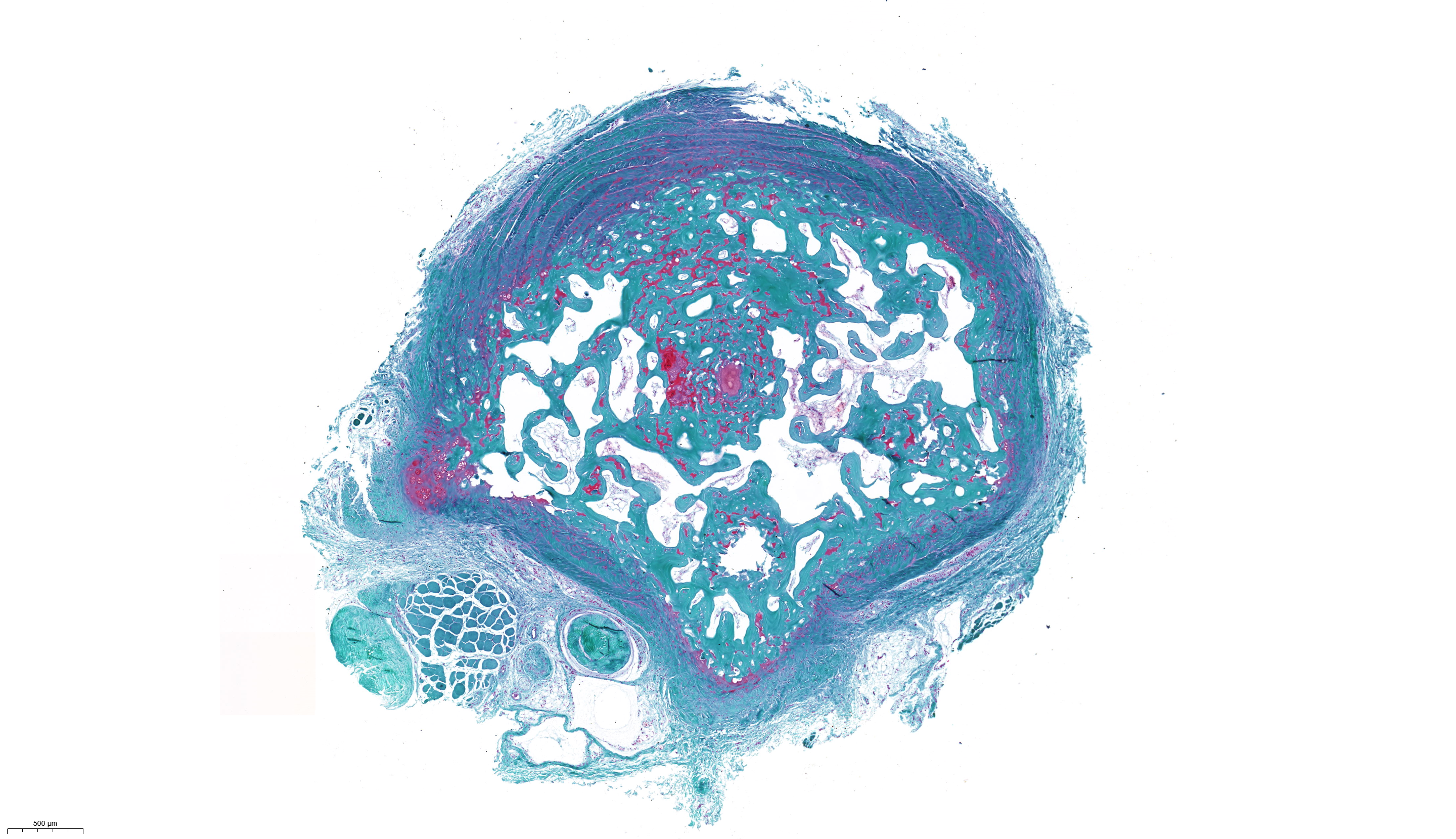

Supplement: S3 File — (ZIP) [file pone.0310014.s003.zip › SOFG triple section image/Model2 SOFG.tif]

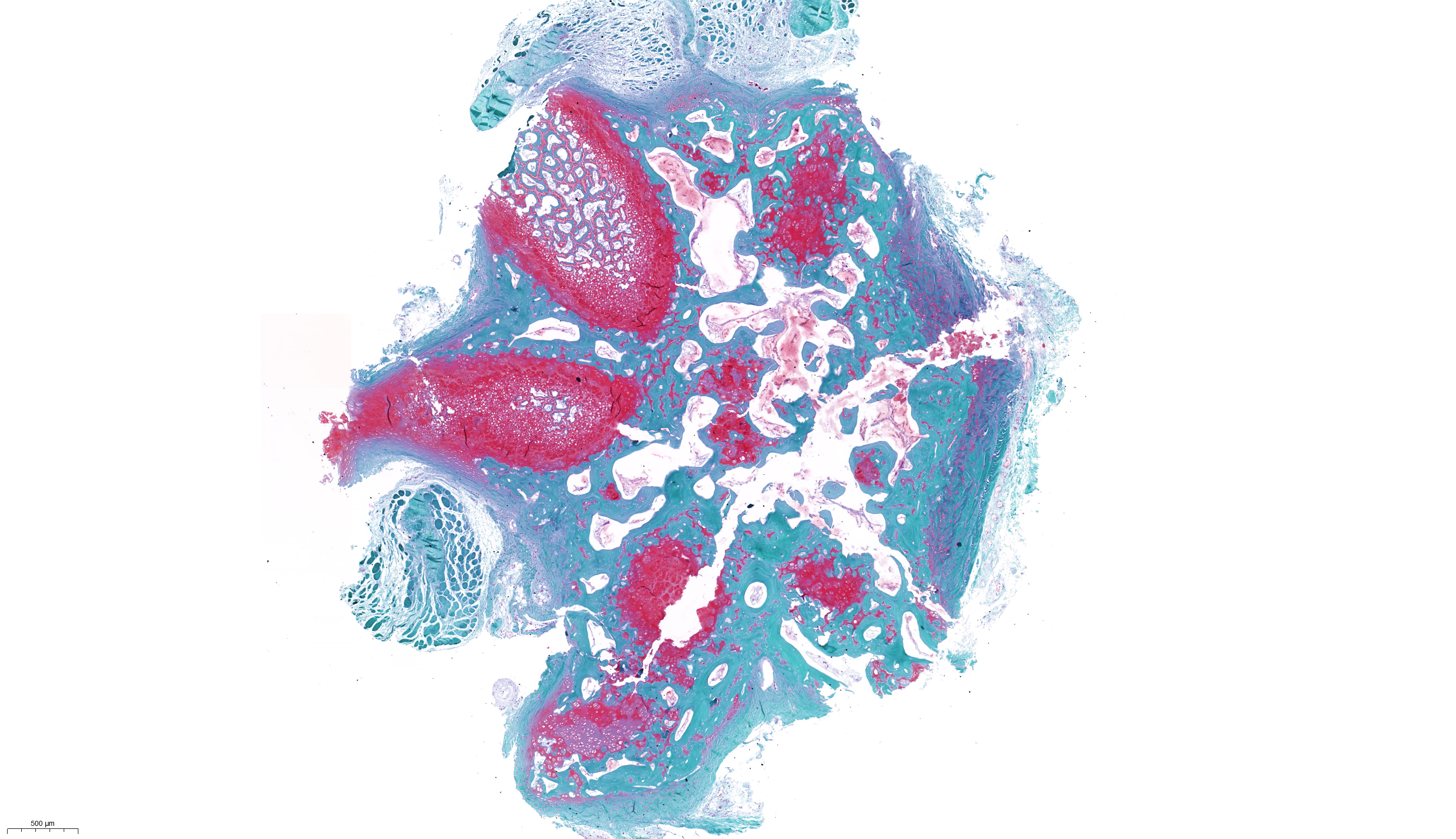

Supplement: S3 File — (ZIP) [file pone.0310014.s003.zip › SOFG triple section image/Model3 SOFG.tif]
